# Potential threats of porcine Getah virus outbreaks and its specific antagonism of host antiviral response through proteasome-dependent degradation of P46 by nsP2

**DOI:** 10.1371/journal.ppat.1014447

**Published:** 2026-08-26

**Authors:** Yang Wu, He Zhang, Mingwei Li, Shanshan Song, Qian Yang, Yaqin Dong, Yongrui Wang, Xuepeng Wang, Ju Zhang, Jin Tian, Feng Zhang, Longjun Guo, Li Feng

**Affiliations:** 1 State Key Laboratory of Animal Disease Control and Prevention, Chinese Academy of Agricultural Sciences Harbin Veterinary Research Institute, Harbin, China; 2 Exotic Herbivore Diseases Surveillance and Research Center, China Animal Health and Epidemiology Center, Qingdao, China; 3 Molecular biology, YEBIO Bio- Engineering Co., Ltd. of Qingdao, Qingdao, China; 4 Laboratory of Medical Genetics, Harbin Medical University, Harbin, China; Stony Brook University, UNITED STATES OF AMERICA

## Abstract

Alphaviruses are a genus of positive-strand RNA viruses classified in the *Togaviridae* family. It contains many important human and animal pathogens, such as chikungunya virus (CHIKV), Sindbis virus (SINV), Venezuelan equine encephalitis virus (VEEV), Semliki Forest virus (SFV) and Ross River virus (RRV), posing significant threats to public health and animal welfare. As a member of genus *Alphavirus*, porcine Getah virus (GETV) is re-emerging around in many parts of the world in recent years (2022–2025) and has caused small to large-scale outbreaks of diarrhea related diseases in pig industry. In this study, a virulent porcine GETV (JLy1 strain) was isolated from the intestinal contents of diarrheic piglets. Using AG129 mouse as an infection model, all the infected mice succumbed to JLy1 infection accompanied by the severe intestinal damages. Further studies indicated that GETV also caused diarrhea and damages to the intestine, lung and spleen in 2-day-old piglets, leading to 100% mortality. Meanwhile, a host protein P46 was screened to be obviously downregulated in GETV infected intestinal tissues, suggesting a potential regulatory relationship between GETV infection and endogenous P46 expression. Ectopic expression of P46 inhibits GETV infection, whereas P46 knockout by CRISPR-Cas9 editing significantly promotes GETV replication, indicating P46 as a novel antiviral regulator against GETV infection. Further studies demonstrated that GETV employs a unique mechanism to antagonizes P46 antiviral response via Lys48-linked proteasome-dependent degradation compared to other alphaviruses, including CHIKV, VEEV, SINV, SFV and RRV. Our findings provide insights into the outbreak risks and immune evasion mechanisms of re-emerging GETV, advancing our understanding of GETV pathogenesis.

## Author summary

Alphaviruses pose a constant threat to public health, and despite their potential for global emergence, there are currently no approved therapies or vaccines available against most of these viruses. In the past few years (2022–2025), the porcine Getah virus (GETV), a member of genus *Alphavirus*, has re-emerged in many parts of the world, triggering diarrhea-related disease outbreaks of varying scales within the pig industry. Understanding the pathogenesis of alphaviruses is essential for the developments of effective antiviral therapeutics. This study reports the successful isolation and characterization of a virulent GETV strain, JLY1, from diarrheic piglets in China. Then, we identified P46 as an antiviral host factor against GETV. Interestingly, unlike CHIKV, VEEV, SINV, SFV and RRV, GETV antagonizes P46 through a unique pathway involving Lys48-linked proteasome-dependent degradation. The present study describes a novel role of the GETV nonstructural protein 2 in targeting P46 to counteract host’s antiviral response, and reveals the mechanism by which GETV can antagonize against P46.

## Introduction

*Alphavirus*es are a genus of enveloped, single-strand positive-sense RNA viruses comprised of a number of medically significant species. Old World alphaviruses such as chikungunya virus (CHIKV), Ross River virus (RRV), and Sindbis virus (SINV) cause debilitating arthralgia whereas New World alphaviruses such as Eastern (EEEV), Western (WEEV), and Venezuelan equine encephalitis viruses (VEEV) cause encephalitis and death [1]. As a member of genus *Alphavirus*, Getah virus (GETV) was first discovered in *Culex gelidus* mosquitoes from Malaysia in 1955 [2]. The particle diameter of GETV is about 60–70 nm with the viral genomic size of 11–11.5 kb. The viral genome contains two open reading frames that encode the four nonstructural proteins (nsP1-nsP2-nsP3-nsP4) and the structural proteins (capsid [C]-E3-E2-(6K/TF)-E1) [1,3]. GETV infection has caused great threats to a variety of animal species, including reproductive disorders and fetal death in pigs, moderate illness in horses, lethal disease in foxes and fever in cattle [4]. Since its first report, GETV has spread throughout East Asia, South Asia, Southeast Asia, and Northern Australia [5]. To date, four major groups (GI-IV) of GETV have been widely reported based on complete genome phylogenetic analysis, and the group III (GIII) has been recognized as the dominant prevailing strains [6].

In recent years 2022–2025, GETV is re-emerging around in many pig-raising farms. Importantly, the prevailing strain evolves to be more susceptible to piglets and sows with increased outbreak frequency and severity compared to that observed approximately 5–10 years ago, posing a potential epidemic risk for the pig industry. A severe disease from an unknown causative agent was reported on a pig farm in Guangdong Province of China from May to July, 2023, which was characterized by abortion and abnormal estrus in sows and diarrhea, hypothermia, edema, ataxia, and death in newborn piglets. GETV named as GDHYLC23 was isolated from lung samples on swine testicle (ST) cells and proved to be the causative agent by metagenomic analysis [6]. Sequence analysis indicated that the epidemic strain GDHYLC23 belonged to the pandemic GIII with an insertion of unique 32-nucleotide repeat in the 3′ noncoding region [6]. The identification of GETV with new variations implies the continuous evolution of the virus, which highlights potential threats to the swine industry. In 2024, another new strain of GETV (GETV-QJ) was isolated from the spleen and intestinal tissues of aborted fetuses in a pig farm in Shanxi Province of China. Additionally, the pathogenicity of GETV-QJ was further evaluated in the different models, including piglets and pregnant sows. It was demonstrated that GETV-QJ infection caused severe diarrhea, fever, and intestinal and lung damage in 7-day-old piglets, resulting in 100% mortality. In contrast, only viremia and reduced survival rates were observed in GETV-QJ-infected pregnant sows, indicating differential pathogenicity of GETV-QJ across pig populations [7]. Furthermore, Lan et al. carried out an epidemiological survey of GETV on 46 pig farms in Jiangxi Province of China. It was shown that 44 out of the 46 pig farms (95.65%) and 197 out of the 411 collected samples (47.93%) were tested positive for GETV, respectively. When inoculated with the isolated GETV, the piglets began to display a series of symptoms, such as high fevers, systemic tremors, lethargy, anorexia and mild diarrhea [8].

Host antiviral proteins are vital in limiting viral infections, and the viruses have evolved redundant mechanisms to counteract the host’s innate immunity for optimal viral adaptation and replication. P46 was first identified as the molecular partner of the p50 subunit of DNA polymerase δ by the yeast two-hybrid assay [9]. And in the following studies, P46 was further screened as binding partners of p70 ribosomal protein S6 kinase 1 (S6K1) and the enhancer of rudimentary (ER), which was involved in the related processes of metabolism and transcription [10,11]. The presence of the RNA recognition motif (RRM, amino acids 277–348) was revealed in the P46 by sequence analysis. Subsequent investigations proves that S6K1 binds P46 within the RRM and can phosphorylate two serines at positions 383 and 385, respectively. Differently, both the RRM and the two phosphorylated serines by S6K1 were encompassed as the bipartite region of P46 (amino acids 274–421) to interact with ER. Based on the data that knockdown of P46 resulted in the smaller cell size, P46 seems to be involved in growth control as the previously reported role of S6K1 [11]. As known, eukaryotic gene expression is a complex stepwise process that begins with transcription initiation, elongation and termination. The production of mature mRNA requires that the nascent pre-mRNA is sufficiently stable to complete its synthesis, processing and export. Due to the feature of nuclear localization and high homology to the Aly⁄REF family of RNA binding proteins, P46 was proposed to be involved in the coupling of transcription, pre-mRNA splicing and transport of mRNA, ultimate governing the biogenesis of transcripts in response to target activation. However, no novel role of P46 is unraveled in the processes of gene expressions and microbial infections, and it needs to be further investigated in the future.

Since around 2022–2025, GETV has re-emerged across the pig industry and has caused small to large-scale outbreaks of diarrhea related diseases. In this study, a virulent porcine GETV (JLy1 strain) was isolated from the intestinal samples in the pig farm with re-emerged outbreaks of GETV. The present study explored the P46 antiviral function to protect the host from virus damage. Moreover, GETV also conversely degraded the host P46 protein through its nsP2 protein with the host proteasome pathway to facilitate GETV replication. These findings highlight the potential risks of GETV outbreaks and deepen our understanding of GETV pathogenesis.

## Results

### Identification of the pathogen responsible for the diarrhea outbreak

**Fig 1:**
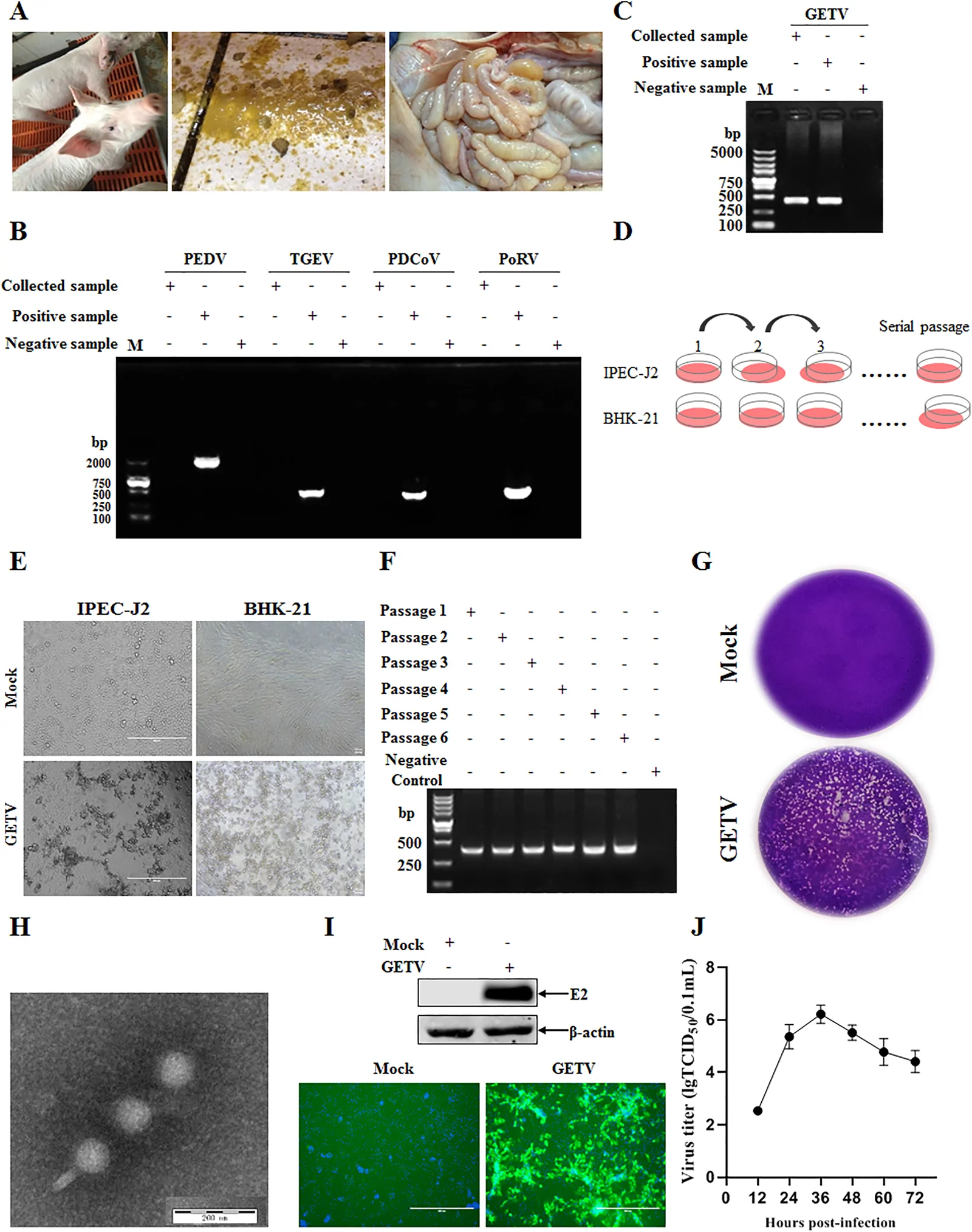
Identification of pathogens. **(A)** Clinical manifestations in piglets infected with GETV. From left to right: depression, diarrhea, and thinning of the intestinal wall. **(B)** RT-PCR detection of common diarrhea pathogens. Lane 1: small intestine tissue sample of diarrhea; Lane 2: small intestine tissue sample of piglets infected with PEDV/TGEV/PDCoV/PoRV as a positive control; Lane 3: non-template control (NTC). **(C)** Identification of GETV in small intestinal tissue samples. Lane 1: sample from piglet with diarrhea; Lane 2: small intestine tissue sample of piglets infected with GETV as a positive control; Lane 3: NTC. **(D)** Schematic diagram of serial passages. **(E)** Propagated blindly for several passages until CPE was observed. IPEC-J2 or BHK-21 cells were infected with GETV. Cytopathic changes were observed at 24 hpi. **(F)** Verification of GETV propagation in IPEC-J2 Cells. Lanes 1-6: propagated transmission of GETV cell culture from the 1^st^ to 6^th^ generation; Lane7: NTC. **(G)** Plaque morphology of GETV using the direct agarose overlay plaque assay. **(H)** Electron microscopy images of GETV-JLy1 particles. Bar = 200 nm. **(I)** Western blotting and immunofluorescence assay of GETV-infected IPEC-J2 cells. Western blotting and immunofluorescence staining were used to detect the presence of GETV-E2 protein in infected IPEC-J2 cells. Nuclei are stained blue with DAPI. **(J)** Growth kinetics of GETV-JLy1 in IPEC-J2 cultures.

In early 2023, an outbreak of epidemic diseases, characterized by severe diarrhea was experienced in a commercial pig farm in Jilin province (Fig 1A). When the necropsies were conducted, the thin-walled intestinal structures with water-like content were observed (Fig 1A). Subsequently, the intestinal tissue samples were collected from the dead pigs to take PCR detections of possible pathogens commonly involved in the viral diarrhea, including porcine epidemic diarrhea virus (PEDV), transmissible gastroenteritis virus (TGEV), porcine deltacoronavirus (PDCoV) and porcine rotavirus (PoRV). As shown in Fig 1B, the samples were tested negative for pathogens of PEDV, TGEV, PDCoV and PoRV by RT-PCR detections. Based on the increasing evidence, GETV might be recognized as the potential pathogen causing diarrhea, abortion and death in newborn piglets [6,12]. Therefore, we questioned that whether GETV infection was involved in the diarrhea disease. It was indicated that the collected intestinal sample was positive for GETV infection by RT-PCR detection (Fig 1C). Furthermore, the virus isolation was carried out by applying the filtered clinical samples (swine intestinal contents) to IPEC-J2 and BHK-21 cells as previously described [13], which are the intestinal target cells or susceptible cell line for GETV replication, respectively. After third serial generations of blind passages (Fig 1D), an obvious cytopathic effect (CPE) was produced characterized by rounding, clumping, and detachment of cells (Fig 1E). When evaluated by RT-PCR detection, the existence of virus replication was confirmed in serial-passaged IPEC-J2 cells with GETV infection (Fig 1F). Given that IPEC-J2 cells are target cells susceptible to GETV replication, they were used for subsequent experiments.

### Biological characterization of the isolated GETV strain

To get the pure GETV particles, a plaque assay was performed. As shown in Fig 1G, plaques with small size were clearly developed at 4–5 days post the inoculation of GETV. Of the plaques, a single clone, designated as GETV-JLy1 was isolated for expansion *in vitro* and used for the following experiments. A negatively stained image showed that the GETV-JLy1 particles are enveloped with a diameter of approximately 70 nm (Fig 1H). When the IPEC-J2 cells were inoculated with the isolated GETV, infection was corroborated by IFA and Western blotting analysis (Fig 1I). Furthermore, the efficient replication of GETV was confirmed in IPEC-J2 cells by the growth kinetic analysis, companying the highest titer up to the 10^6.22^ 50% tissue culture infective doses (TCID_50_)/0.1 mL at 36 h post infection (hpi) (Fig 1J). The whole genome sequence of GETV-JLy1 strain obtained by gene sequencing was 11689 nucleotides (nt) in length. The sequence data have been deposited in the GenBank and the accession number is PP236766.1. When aligned with the genome-wide sequences of 9 representative strains, JLy1 shows the highest and lowest similarity of nucleotide sequences with GETV strain GD202202 (99.4%) and MM2021 (95.3%), respectively (S1 Fig). Phylogenetic analysis indicates that JLY1 is classified to the cluster of Group III, which is the dominant prevailing strain in recent years (S2 Fig).

**Fig 2:**
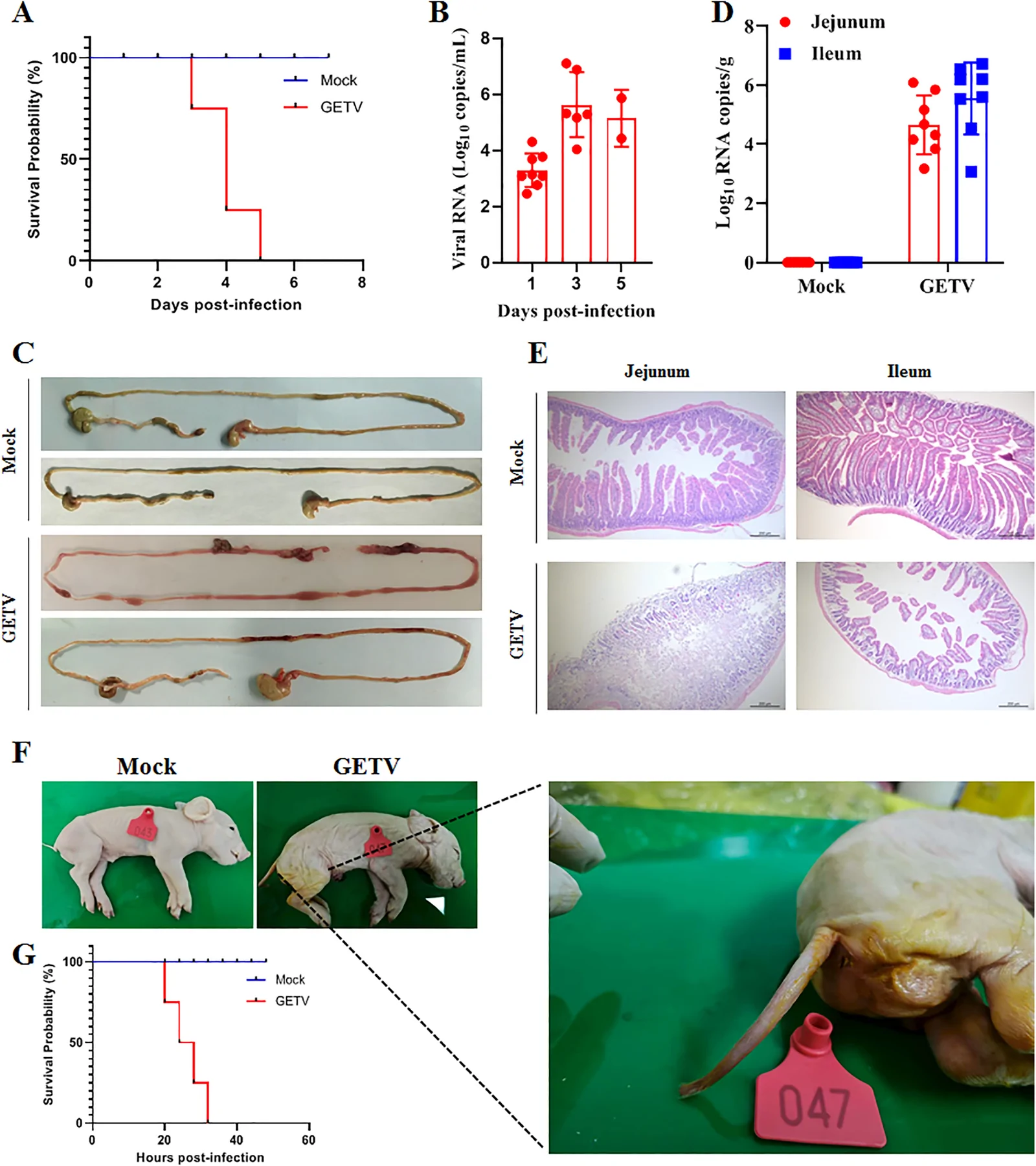
Pathogenicity of GETV-JLy1 in AG129 mice and piglets. **(A)** Survival curves of AG129 mice with GETV. **(B)** Viremia changes in AG129 mice infected by GETV. **(C)** Representative lesions of stomach, small intestine, large intestine in GETV-infected mice. **(D)** QRT-PCR detection of GETV loads in jejunum and ileum tissues of GETV-infected mice. **(E)** H&E staining of lesions in jejunum and ileum tissues of GETV-infected mice. **(F and G)** Pathogenicity of GETV-JLy1 in piglets. **(F)** Presence of clinical diarrhea post GETV infection. **(G)** Survival curves of piglets in each group during GETV challenge study.

It has been reported that the AG129 mouse, a double-knockout model lacking receptors for both type I (α, β) and type II (γ) interferon (IFNAR^−/−^ IFNGR^−/−^DKO) is susceptible to analyze arthropod-borne virus infection from *Flaviviridae* and *Togaviridae* families [14–16]. To determine the pathogenicity of the isolated GETV, the infection studies were performed by using the AG129 model. Here, we inoculated AG129 mice with the isolated GETV through subcutaneous injection and analyzed the survival rates for eight days. An early and high mortality rate was observed for GETV infection in this study, showing 100% mortality only in five days post infection (dpi) (Fig 2A). To investigate the kinetics of viremia, the blood was collected to estimate the levels of viral loads by qRT-PCR. A great abundance of viral RNA was observed in the blood from 1 dpi to death (Fig 2B). As shown in the autopsy, severe intestinal damages were observed, showing thin and transparent intestinal walls, luminal accumulation of large amounts of watery liquid and hemorrhage in GETV infected intestinal tissues (Fig 2C). In addition, high levels of GETV RNA were detected in the jejunum and ileum (average: 10^5^ copies equivalents/g tissue) (Fig 2D). Histological analysis showed that desquamations of epithelial cells of the intestinal villi were obviously observed from the lamina propria of GETV infected intestinal mucosa, no significant pathological changes were observed in the control group (Fig 2E).

The pathogenicity of GETV-JLy1 was further evaluated in neonatal piglets. The first piglet appeared obvious diarrhea (Fig 2F) and began to die at 19 hours post infection (hpi), and all the piglets died within 29 hpi. Conversely, no piglets died in the mock group (Fig 2G). Meanwhile, tissue autopsy illustrated that GETV infection resulted in obvious thinned intestine, mesenteric hemorrhage and lesions in lungs and spleens (S3A Fig). Histological analysis showed that necrosis and desquamations of epithelial cells of the intestinal villi were obviously observed from the lamina propria of GETV infected intestinal tissues. Besides, white pulp atrophy along with the elevated accumulations of red blood cells in red pulp was present in GETV infected spleen tissues (S3B Fig). No significant pathological changes were present in the control group (S3B Fig).

### P46 overexpression restricts GETV replication

**Fig 3:**
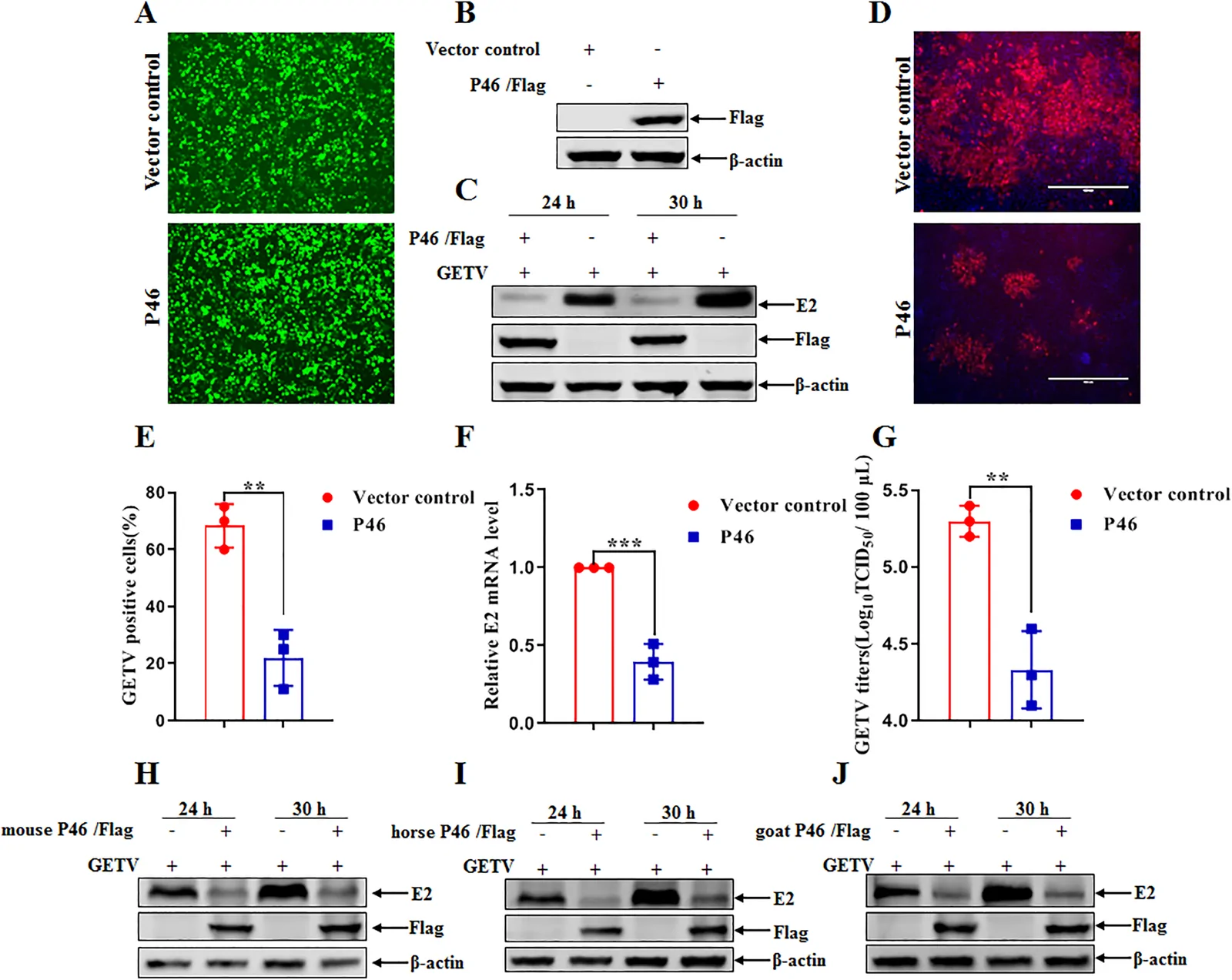
P46 overexpression restricts GETV replication. **(A)** IPEC-J2 cells were transduced with a bicistronic lentivirus vector designed to express either ZsGreen or P46. **(B)** Western blotting analysis of P46 expression. **(C)** Western blotting analysis demonstrated that P46 negatively regulated the proliferation of GETV. IPEC-J2 cells stably expressing ZsGreen or P46 were infected with GETV and harvested at 24 and 30 hpi, followed by immunoblotting with indicated antibodies. **(D)** IFA revealed that overexpression of P46 restrained GETV infection. IPEC-J2 cells stably expressing ZsGreen or P46 were infected with GETV at an MOI of 1 for 24 **h.** The cells were then fixed and stained with GETV E2 antibodies and the nuclear marker DAPI. **(E)** The percentage of GETV-infected cells was determined by IFA. **(F)** Ectopic expression of P46 suppressed GETV infection, as measured by qRT-PCR. Quantification of viral E2 mRNA by qRT-PCR, and the data are expressed as fold changes relative to the control group. **(G)** P46 overexpression restricted GETV replication, as indicated by TCID_50_. The viral titer of GETV was determined using a TCID_50_ assay. **(H-J)** Western blotting analysis demonstrated that P46 from various species negatively regulated the proliferation of GETV. Cells expressing P46 from various species were infected with GETV and harvested at 24 and 30 hpi. Protein expression was then analyzed by immunoblotting using the indicated antibodies. The results represent three independent experiments (the means ± SD). **, *P* < 0.01, ***, *P* < 0.001. The *P* value was calculated using Student’s t-tests.

In order to investigate whether GETV infection can influence various pathogen-associated molecular pattern receptors and host protein expression. The intestinal tissues were collected for the examination of pattern receptors and host protein expression. Western blotting demonstrated that a host protein P46 was screened to be obviously downregulated in GETV infected intestinal tissues compared with that in mock-infected mice (S4 Fig). Besides P46, several other proteins are also greatly down- (e.g., MDA5, IRF3, TRIM24) or upregulated (e.g., RINT1, TRIM21, OAS1), which there are direct link with the immune response of the host cells, and we will investigate interactive regulatory mechanisms in the future. To evaluate the role of P46 in the regulation of GETV infection, IPEC-J2 cells stably expressing P46 were constructed by lentivirus transduction. The ectopic expression of P46 was validated by IFA and Western blotting (Fig 3A and 3B). As shown in Fig 3C, ectopic expression of P46 led to a significant reduction in GETV replication, as demonstrated by Western blotting analysis. Similar results were obtained in BHK-21 cells (S5A Fig). Consistent with the results from Western blotting data, the level of GETV replication was also limited in IPEC-J2 cells overexpressing P46 when determined by IFA (Fig 3D and 3E), quantitative real-time reverse transcription PCR (qRT-PCR) (Fig 3F) and TCID_50_ assay (Fig 3G), respectively. Because GETV as a multi-species veterinary pathogen, we analyzed the protein sequences of P46 from different species and the results showed that P46 is relatively conserved among different species, with a sequence identity of 89.1% to 96.7% (S6 Fig). Then we examined the inhibitory effect of P46 from different species on GETV replications. The Western blotting analysis demonstrated that the overexpression of P46 from different species led to a significant reduction in GETV replication (Fig 3H-3J). Given that GETV belongs to the *Togaviridae* family, we hypothesized that P46 might serve as a restriction factor for other members in this family. The recombinant SINV expressing the GFP (SINV-GFP) was selected as a representative, and the results showed that the replication of SINV-GFP was reduced following P46 overexpression (S7A- S7D Fig). Altogether, these findings indicate that the GETV replication was restricted by P46 overexpression in IPEC-J2 cells.

### Knockout of P46 enhances GETV infection

**Fig 4:**
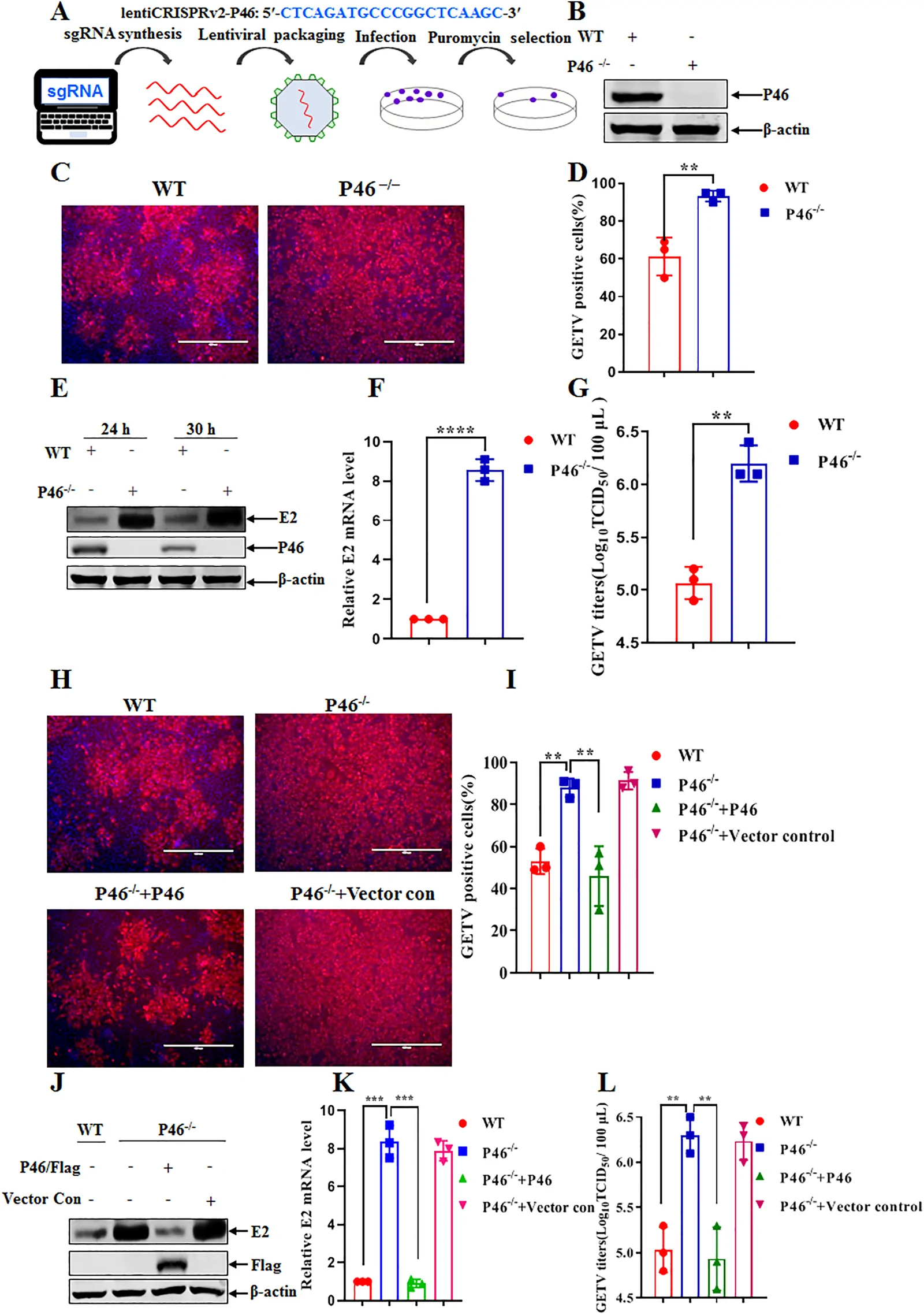
Loss of P46 enhances GETV replication. **(A)** CRISPR-Cas9-mediated P46 knockout. CRISPR-Cas9 technology was used to generate a P46 knockout (P46^-/-^) in IPEC-J2 cells. **(B)** Western blotting confirmation of P46 knockout. **(C to G)** P46 knockout promoted GETV replication. WT and P46^-/-^ cells were infected with GETV at an MOI of 1 and harvested. GETV replication was evaluated by IFA **(C and D)**, Western blotting **(E)**, qRT-PCR **(F)**, and TCID_50_ **(G)**, respectively. **(H to L)** Restoration of P46 expression negatively regulated the proliferation of GETV. P46^-/-^ cells were stably reconstituted with either a vector control or P46 through lentivirus transduction system. These cells were then infected with GETV at an MOI of 1 for 24 **h. (H)** Fixed cells were stained with specific antibodies. **(I)** The percentage of GETV-infected cells was determined by IFA. **(J)** Protein levels of P46, viral E2, and β-actin were analyzed by Western blotting. **(K)** Quantification of viral E2 mRNA by qRT-PCR. **(L)** Quantification of GETV viral titers. The results represent three independent experiments (the means ± SD). **, *P* < 0.01, ***, *P* < 0.001, ****, *P* < 0.0001. The *P* value was calculated using Student’s t-tests.

To further evaluate the inhibitory role of P46 in GETV replication, an IPEC-J2 cell line with knockout (KO) of *p46* gene (P46^-/-^) was constructed by using the CRISPR-Cas9 technique followed by the single clone screening with puromycin treatment (Fig 4A). Three P46 KO clones were obtained with an insert of adenine in P46 opening reading frame by sequencing analysis, resulting in the disruption of P46 translation (S8 Fig). The knockout of P46 expression was further confirmed by Western blotting analysis (Fig 4B). Next, the wild type (WT) or P46^-/-^ cells were infected with GETV, and the level of virus infection was assessed by IFA, Western blotting, qRT-PCR, and TCID_50_ assay, respectively. IFA results demonstrated that GETV infection was apparently enhanced in P46^-/-^ cells compared to that in WT cells (Fig 4C and 4D). In line with the IFA data, the level of GETV replication was markedly increased in the P46^-/-^ cells when determined by Western blotting (Fig 4E), qRT-PCR (Fig 4F), and TCID_50_ assay (Fig 4G), respectively. Collectively, P46 knockout facilitates the replication of GETV in IPEC-J2 cells.

To further validate the antiviral activity of P46 against GETV, the restoration of P46 expression was established in P46^-/-^ cells. After 24 h transduction with lentiviruses to express the P46 protein or vector control, the cells were inoculated with GETV for another 24 h. Compared to P46^-/-^ cells with transduction of lentivirus control, GETV replication was obviously inhibited in P46^-/-^ cells with lentivirus-mediated trans-complementation of P46, corroborating the antiviral activity of P46 against GETV infection (Fig 4H and 4I). The replication of GETV was disrupted following the ectopic expression of P46 in P46^-/-^ cells, as evaluated by Western blotting (Fig 4J). Furthermore, when determined by qRT-PCR, the ectopic complementation of P46 resulted in a substantial decrease in GETV E2 mRNA amount in P46^-/-^ cells (Fig 4K). Consistent with the previous results, a clear decrease in progeny virus was further confirmed by TCID_50_ assay in the GETV-infected P46^-/-^ cells with P46 restoration (Fig 4L). In summary, the ectopic expression of P46 compromised the elevated replication of GETV in the P46^-/-^ cells, confirming the antiviral function of P46 against GETV infection.

Next, we checked whether the P46 knockout affected the attachment and internalization of GETV infection. The results showed that there was no significant difference in the attachment and internalization efficiency between WT cells and P46^-/-^ cells (S9A and S9B Fig). Furthermore, we explored whether P46 might regulate GETV RNA synthesis. The subgenomic GETV-Luc replicon (GETV-Luc) was constructed by replacing the viral structural genes (capsid, E3, E2, 6K, and E1) with a luciferase reporter gene under the subgenomic promoter. The schematic diagram of GETV subgenomic replicon was indicated in S9C Fig. GETV replicon RNA-Luc was synthesized by using SP6 In Vitro Transcription Kit, and then co-transfected along with pRL-TK into WT and P46^-/-^ cells, respectively. As shown in S9D Fig, the luciferase activity was obviously increased in P46^-/-^ cells, indicating that the P46 expression inhibited GETV RNA synthesis.

### GETV infection induces P46 degradation via the ubiquitin-proteasome system

**Fig 5:**
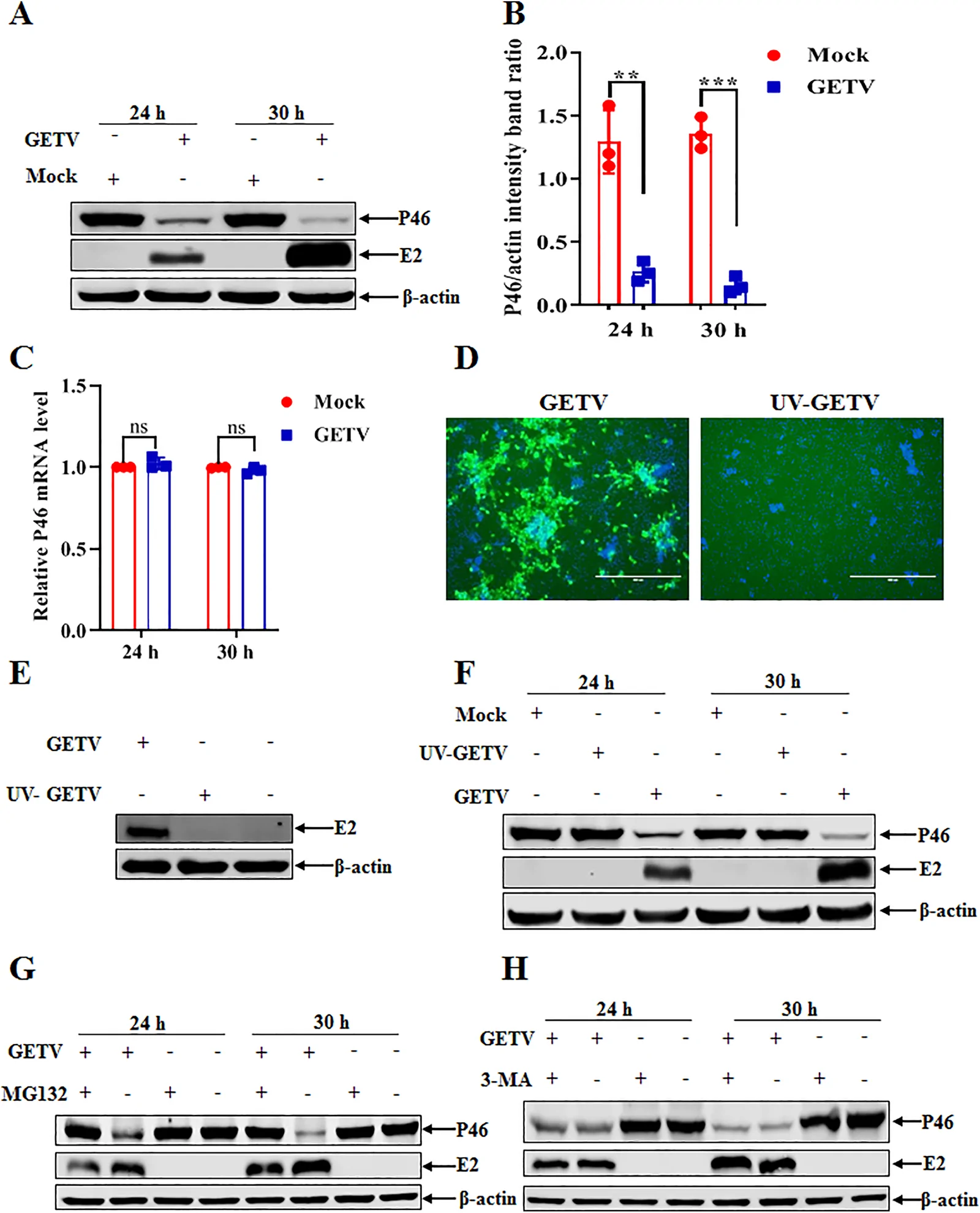
Proteasome-mediated degradation, rather than autophagy, plays a pivotal role in the reduction of P46 triggered by GETV infection. **(A and B)** P46 expression was downregulated upon GETV infection, as detected by Western blotting. IPEC-J2 cells were infected with GETV at an MOI of 1 and harvested at 24 and 30 hpi. **(A)** Protein levels of P46, viral E2, and β-actin were analyzed by Western blotting. **(B)** Relative intensities of P46 were normalized to β-actin. **(C)** Quantification of P46 mRNA by qRT-PCR in GETV-infected IPEC-J2 cells. **(D and E)** IPEC-J2 cells were infected with either active GETV or UV-inactivated GETV at indicated times. **(D)** Cells were fixed and stained with the indicated antibodies. **(E)** Protein levels of viral E2, and β-actin were analyzed by Western blotting. **(F)** The target cells were performed as in panel **(A)**, and protein levels of P46, viral E2, and β-actin were analyzed by Western blotting. **(G)** Treatment with MG132, a proteasome inhibitor, effectively blocked the degradation of P46 induced by GETV. IPEC-J2 cells were pretreated with either MG132 for 1 hour. Subsequently, the cells were infected with GETV or left uninfected. Following infection, the cells were further cultured in the presence or absence of MG132 for varying time intervals. Cell lysates were prepared using detergent and subjected to SDS-PAGE under reducing conditions to separate the proteins. Immunoblotting was performed using antibodies specific to P46, GETV E2 protein, and β-actin. **(H)** Autophagy did not contribute to the reduction of P46 observed during GETV infection. IPEC-J2 cells were pretreated with 3-MA for 4 hours prior to GETV infection. Subsequently, the levels of P46 were assessed at specified time point post-infection using Western blotting analysis. The results represent three independent experiments (the means ± SD). ns, no significant, **, *P* < 0.01, ***, *P* < 0.001. The *P* value was calculated using Student’s t-tests.

Although we have demonstrated that P46 negatively regulates the replication of GETV, it remains unclear about the regulation of P46 expression in the context of GETV infection. We carried out the infection experiment to examine the endogenous expression of P46 in GETV-infected IPEC-J2 cells. As shown in Fig 5A and 5B, the expression of P46 was dramatically downregulated after GETV infection compared with that in mock-infected cells. Consistent results were observed in BHK-21 cells (S5B Fig). Likewise, endogenous P46 levels were downregulated following SINV-GFP infection (S10 Fig). Then, we questioned whether the reduction of endogenous P46 was resulted from the decreased transcription of P46 gene. When evaluated by qRT-PCR, the mRNA transcription level of P46 was not changed in GETV-infected IPEC-J2 cells (Fig 5C), suggesting that the reduced protein was not due to the inhibition of P46 transcription but might be attributed to post-transcriptional modifications.

To explore whether the P46 reduction was dependent on viral replication, we inactivated GETV virions through UV illumination followed by verification with IFA and Western blotting (Fig 5D and 5E). When the cells were inoculated with UV-inactivated virus, the endogenous expressions of P46 remained unaffected and were similar to those in mock-infected cells (Fig 5F), indicating that active virus replication is required for GETV-mediated P46 decrease. Next, we speculate that protein degradation might be involved in GETV-mediated P46 reduction. As well known, the ubiquitin-proteasome system and autophagy are recognized as two major degradation pathways in the eukaryotic cells [17]. Subsequently, IPEC-J2 cells were infected with GETV, followed by treatment with proteasome inhibitor MG132, autophagy inhibitor 3-MA or dimethyl sulfoxide (DMSO) carrier control. The results demonstrate that P46 downregulation was blocked by proteasome inhibitor (Fig 5G) but not by autophagy inhibitor (Fig 5H), strongly implicating the involvement of ubiquitin-proteasome degradation system in GETV induced P46 reduction. These results suggest that GETV infection induces P46 degradation in a ubiquitin-proteasome mediated manner.

### GETV nsP2 mediated K48-linked ubiquitination and degradation of P46

**Fig 6:**
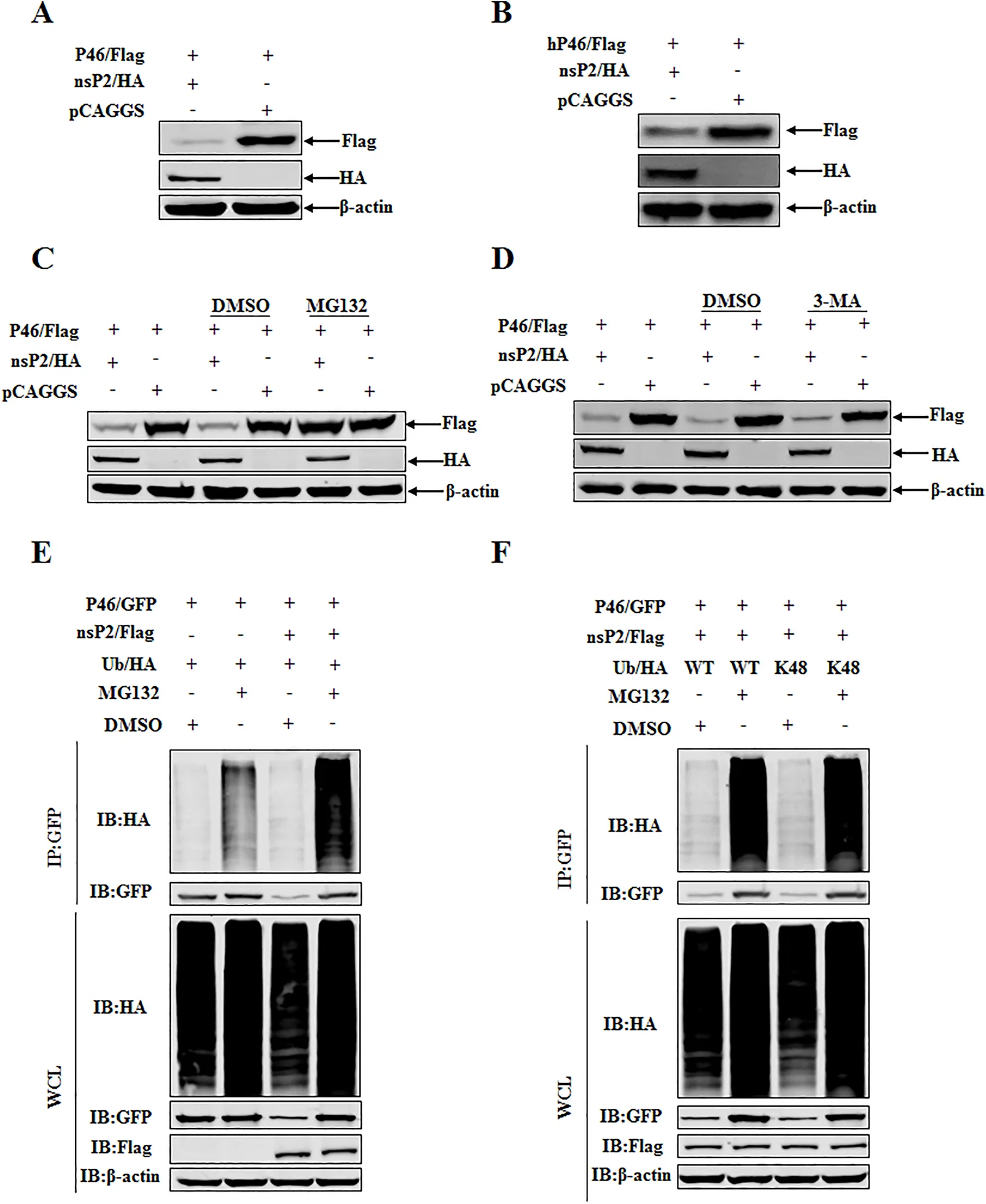
GETV nsP2 promotes K48-ubiquitination and proteasome-dependent degradation of P46. **(A and B)** NsP2 triggered the reduction of P46. Cells were co-transfected with plasmids encoding porcine Flag-tagged-P46 (P46/Flag) (A) or human P46/Flag (hP46/Flag) (B) and HA-tagged GETV nsP2 (nsP2/HA) or vector control. After 24 hours of transfection, immunoblotting was performed using antibodies against the HA tag, Flag tag, or β-actin. **(C and D)** NsP2 promoted the degradation of P46 in a proteasome way. HEK293T cells were transfected with plasmids encoding P46/Flag and nsP2/HA. Subsequently, cells were treated with either MG132 or 3-MA. Immunoblotting was performed using antibodies specific to HA tag, Flag tag, or β-actin. **(E and F)** NsP2 promotes K48-ubiquitination and proteasome-dependent degradation of P46. HEK293T cells were transfected with HA-tagged ubiquitin (Ub/HA) or HA-tagged Ub mutant (K48/HA), GFP-tagged P46 (P46/GFP) and Flag-tagged GETV nsP2 (nsP2/Flag), cells were treated with MG132. After 36 hours of culture, cells were harvested, and IP was performed using antibodies against GFP. Both whole-cell lysates (WCLs) and IP complexes were analyzed by immunoblotting with antibodies specific to GFP tag, HA tag, Flag tag, or β-actin.

The established results have demonstrated that GETV infection induced P46 degradation via ubiquitin-proteasome pathway. However, it remains unknown about what virus-encoded proteins involved in P46 degradation. To dissect the viral protein responsible for P46 degradation, HEK293T cells were co-transfected with Flag-tagged P46 and each viral encoded protein followed by P46 detection by Western blotting analysis (S11 Fig). As shown in Fig 6A, the GETV-encoded non-structural protein 2 (nsP2) was verified to participate in the P46 reduction.

GETV has broad range of host tropism and can infect humans [18]. To investigate whether the antagonism of nsP2-mediated P46 degradation is conserved across species, HEK293T cells were co-transfected with plasmids expressing human P46 (94.8% identity with porcine P46) and nsP2. As expected, similar reduction of human P46 was also induced by nsP2 (Fig 6B). We then questioned that whether nsP2 mediated human P46 reduction was also mediated through ubiquitin-proteasome pathway. Plasmids expressing human P46 along with nsP2 were co-transfected into HEK293T cells followed by treatment with proteasome inhibitor MG132 or autophagy inhibitor 3-MA. Similar to data from GETV infection (Fig 5G), the transfection of nsP2 alone resulted in degradation of human P46. Furthermore, the degradation was restored by MG132 treatment (Fig 6C) but not by 3-MA treatment (Fig 6D). Similar results were also observed in BHK-21 cells (S5C Fig).

To further investigate the mechanism of ubiquitin-proteasome-mediated P46 degradation by nsP2, HEK293T cells were co-transfected with Flag-tagged nsP2, GFP-tagged P46 and HA-tagged Ub plasmids to evaluate the ubiquitination level of P46. Cells were also immunoprecipated for P46 with treatment of MG132 or DMSO followed by immunoblotting analysis for ubiquitin. We observed that the expression of nsP2 enhanced the levels of ubiquitinated P46 (Fig 6E). It has also been well established that K48-linked ubiquitination chains play critical roles in substrate degradation by proteasome-mediated pathway [19]. To determine the involvement of K48-linked ubiquitination in P46 degradation, HEK293T cells were co-transfected with Flag-tagged nsP2, GFP-tagged P46 and HA-tagged the wild-type Ub (Ub-WT) or HA-tagged Ub-K48 (Ub-K48) plasmids, accompanied by treatment of MG132 or DMSO. As expected, GETV nsP2 mediated K48-linked ubiquitination and degradation of P46 (Fig 6F). Collectively, these results confirmed that GETV nsP2 induced the degradation of P46 through proteasome-mediated pathways via the K48-linked ubiquitination.

### Specific antagonism of P46 by GETV nsP2 among alphavirus family

**Fig 7:**
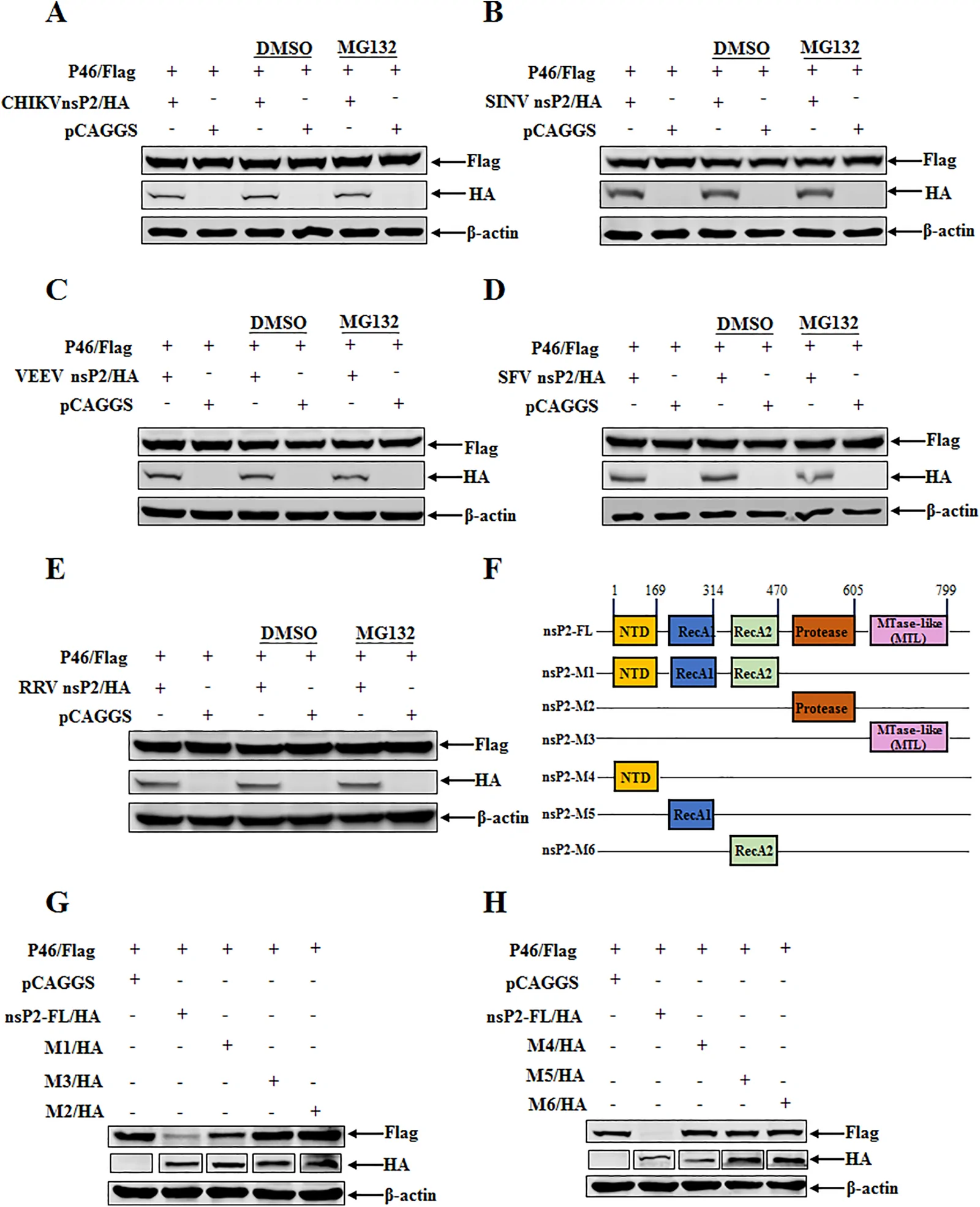
Unique antagonism of P46 antiviral activity by GETV nsP2 compared to other alphaviruses of CHIKV, VEEV, SINV, SFV and RRV. **(A to E)** HEK293T cells were transfected with P46/Flag along with plasmids encoding HA-tagged nsP2s from CHIKV **(A)**, SINV **(B)**, VEEV **(C)**, SFV (D) and RRV **(E)**, respectively. At 24 h post-transfection, cells were lysed and detected by immunoblotting. **(F)** The schematic diagram of the structure of nsP2 and nsP2 N-terminal. **(G)** The N-terminal region was mainly responsible for the degradation of P46 induced by nsP2. HEK293T cells were co-transfected with plasmids encoding P46/Flag and nsP2/HA or different mutants. After 24 hours of transfection, immunoblotting was performed using antibodies against the HA tag, Flag tag, or β-actin. **(H)** HEK293T cells were co-transfected with P46/Flag and nsP2/HA or different mutants plasmids. After 24 hours of transfection, immunoblotting was performed using antibodies against the HA tag, Flag tag, or β-actin.

The established data has demonstrated that the antiviral activity of P46 was antagonized by GETV nsP2. We then questioned that whether the similar antagonistic activity was present in other members in Alphavirus, such as CHIKV, SINV, VEEV, SFV and RRV. The corresponding nsP2 gene fused with a HA tag at the C-terminus was co-expressed with P46 in HEK293T cells to analyze the levels of P46 protein. Surprisingly, no obvious decrease in P46 was observed in HEK293T cells with expression of nsP2 from CHIKV, SINV, VEEV, SFV, and RRV (Fig 7A-7E). Subsequently, the addition of the proteasome inhibitor MG132 did not alter these nsP2 mediated expression of P46 (Fig 7A-7E). To investigate whether other viral proteins may have similar functions to GETV nsP2, we chose SFV and SINV as representatives to evaluate P46 degradation in the presence of their individual viral proteins. Compared with GETV nsP2 mediated obvious reduction of P46, there was no other viral proteins screened to significantly decrease the expression of P46 (S13A and S13B Fig), although some viral encoded proteins were not expressed, which need to be further investigated in the future.

The alphavirus nsP2 protein contains the viral helicase, protease, and a putative C-terminal methyltransferase domain, and it interacts with numerous host proteins [20]. Next, to map the domain(s) of nsP2 associated with P46 degradation, GETV nsP2 was truncated into three mutant fragments with deletions of major motifs as mentioned in Fig 7F. HEK293T cells were transfected with a range of plasmids encoding full-length or truncated nsP2. The N-terminal region aa 1–470 of nsP2 obviously induced P46 degradation, but the extent of P46 degradation was comparably less than that induced by the full-length nsP2 (Fig 7G).

Besides, the N-terminal region aa 1–470 of nsP2 encompassed an N-terminal domain (NTD) and two RecA-like domains (Fig 7F). Subsequently, plasmids expressing each of these three nsP2 domains and P46 protein were co-transfected into HEK293T cells to analyze the expression of P46. The results indicated that these individual domains no longer induced P46 degradation, speculating the importance of spatial configuration integrity in aa 1–470 induced P46 reduction (Fig 7H). Collectively, nsP2-induced proteasomal degradation of P46 protein is specific to GETV compared to other alphaviruses of CHIKV, VEEV, SINV, SFV and RRV, and the aa 1–470 of GETV nsP2 is identified as the crucial determinant responsible for the degradation of P46.

## Discussion

GETV is an emerging and widespread zoonotic arbovirus capable of infecting humans and a broad range of mammals [4,21]. Outbreaks of GETV infection in domestic animals have been reported in Asia, Australia, and Europe [5,12,22,23]. In China, GETV was first isolated from wild Culex mosquitoes in Hainan Province in 1964 [24]. Subsequently, GETV has been detected in different hosts in 21 provinces and cities across China, including Yunnan, Sichuan, Guizhou, Gansu, Jilin, Hebei, Guangdong, and Shanxi [6,25,26]. During the period from 2022 to 2025, GETV became a recurring epidemic pathogen in pig farms [12]. Infected newborn piglets exhibit symptoms such as diarrhea, hind limb paralysis, depression, and high mortality rates. Pregnant sows may experience abortion and vertical transmission to their offspring [27]. The prevalence, range, incidence rate, and mortality due to GETV have increased significantly in pig farms worldwide, causing huge economic losses to the pig industry [28]. The virus poses a growing threat to animal health and agricultural productivity, prompting deep research into its pathogenesis, transmission dynamics, and vaccine development. In this study, GETV was isolated from pigs that exhibited severe disease and died in a commercial pig farm in Jilin province. GETV-JLy1 was identified as a member of GIII. The homology of GETV-JLy1 was 97.7%–99.4% and 95.3%–97.3% within the same and different subtypes, respectively.

Host innate immune plays a crucial role in antiviral defense against invading pathogens [29–31]. However, alphaviruses are reported to suppress the induction of the innate immune response during infection through different mechanisms. It has been documented that nsP2 of GETV and CHIKV can impair IFN-β induction and the subsequent JAK/STAT signaling by blocking the phosphorylation and nuclear translocation of IRF3 and/or STAT1 [32,33]. In addition, inhibition of STAT phosphorylation and translocation to the nucleus were also observed in VEEV and SINV-infected cells, resulting in synthetic suppressions of IFN-α/β and ISGs [34]. Besides, host shut-off was induced to restrict the protein synthesis of cells by a couple of viruses, including alphaviruses [35–37]. Within the first few hours post infection, alphaviruses induced transcriptional and translational shutoffs, which blocked the formation of stress granules [38]. Rpb1 is a catalytic subunit of polymerase II (RNAPII) that catalyzes the polymerase reaction during RNA transcription and plays an indispensable role in transcriptional activation of cellular genes [37]. Akhrymuk et.al. delineated that alphavirus evolves another novel mechanism to evade the innate immune response by nsP2 induced rapid degradation of Rpb1, blocking the cellular antiviral response post virus infection [37]. In this study, a couple of other proteins with obvious downregulation (e.g., MDA5, IRF3, TRIM24) or upregulation (e.g., RINT1, TRIM21, OAS1) are also observed in the intestinal tissues post GETV infection (S4 Fig). The mentioned proteins are involved in diverse regulations of host immune response. Frolova et al. have reported that cellular pattern recognition receptor MDA5 was found to play an important role in the induction response of anti-alphavirus. Together with RIG-I, MDA5 can sense the replicating alphaviruses and determine activation of the antiviral defense [39]. As for the detailed functions of other identified proteins in the context of alphavirus infection, however, the regulatory mechanisms of the virus-host interactions remain unrevealed, and we will carry out the subsequent investigations in the following studies.

P46, a key protein that interacts with DNA polymerase delta, has been recognized for its pivotal roles in various biological processes [40]. However, the intersection between P46 expression and virus infection remains a relatively unexplored area. Firstly, our investigation demonstrates that overexpression P46 effectively suppresses GETV and SINV infection, whereas CRISPR-Cas9-mediated knockout of P46 significantly enhances GETV replication, implying the antiviral effects of P46 on GETV and SINV infection. Notably, the decrease of P46 was mediated by the nsP2 protein of GETV. Unlike the specific antagonistic strategy by GETV nsP2, P46 degradation was not shared as the common mechanism for all nsP2 of alphaviruses, such as CHIKV, SINV, VEEV, SFV and RRV, indicating the complexity between virus infection and host response. When the amino acid sequences of nsP2 from GETV, CHIKV, SINV, VEEV, SFV and RRV were aligned (S12 Fig), it seemed difficult and complicated to unveil the specific difference involved in P46 degradation by GETV nsP2. It is well-known that the structure and function of proteins are closely linked, and even if subtle differences in spatial structure can lead to significant disruption of function activity. Next, we tried to analyze the difference in nsP2 mediated degradation from alphavirus, by dissecting the structural characteristics of the diverse nsP2. When predicting structural features of the nsP2 proteins, we observed that while there are conserved regions among them, there are also significant differences. These differences may account for the distinct functional outcomes observed in our experiments. In conclusion, our results suggest that the P46 degradation by nsP2 is a specific effect of GETV and is not shared by other tested alphaviruses. This specificity may be due to structural or functional differences in the nsP2 proteins of these viruses.

**Fig 8:**
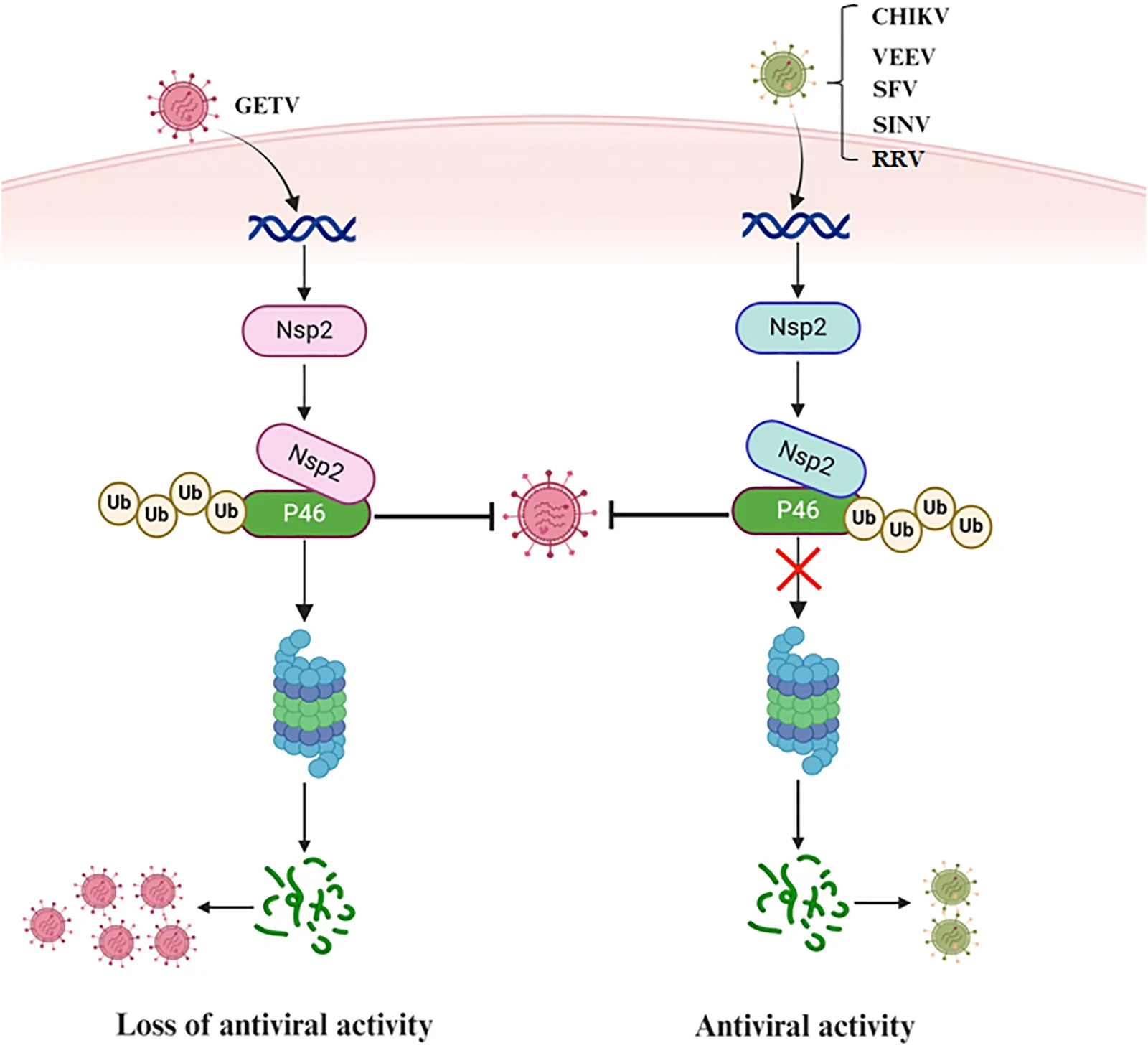
Schematic depiction of the specific antagonistic role of P46-mediated antiviral activity by GETV nsP2. GETV, not CHIKV, SINV, VEEV, SFV, and RRV, nsP2-mediated K48-linked polyubiquitination and proteasome-dependent degradation of P46 to evade host immune response.

In summary, we demonstrated that P46 serves as a novel antiviral regulator capable of negatively modulating alphavirus infection. Furthermore, nsP2 protein facilitates Lys48-linked polyubiquitination of P46, resulting in its proteasome-dependent degradation and subsequently antagonizing the antiviral function of P46 (Fig 8). These findings offer valuable insights into the antiviral immune evasion mechanisms employed by alphaviruses, enhancing our comprehension of their pathogenesis.

## Materials and methods

### Ethics statement

The procedure for animal experiment was reviewed and approved by the Animal Ethics Committee of the School of Harbin Veterinary Research Institute of the Chinese Academy of Agricultural Sciences. The Animal Ethics Committee approval number was 250826–03-GR and 250109–04-GR. All animals were performed in accordance with animal ethics guidelines and approved protocols.

### Cell culture and viruses

IPEC-J2 cells (a porcine small intestinal epithelial cell line), BHK-21 cells (ATCC CCL-10) and HEK293T cells (ATCC CRL-3216) were cultured in Dulbecco’s minimum essential medium (DMEM) (Life Technologies, USA) supplemented with 10% heat-inactivated fetal bovine serum (FBS) (Gibco, USA) at 37 °C with 5% CO_2_. Intestinal tissue samples were collected from piglets with diarrhea from a medium-scale pig farm in Jilin, China. GETV-JLy1 strain was isolated in this study and the sequence data has been deposited in the GenBank with the accession number of PP236766.1. The recombinant SINV-GFP as another representative from Togaviridae family was kindly provided by Dr. Xin Yin from Harbin Veterinary Research Institute.

### RNA extraction and RT-PCR analysis

The intestinal tissue samples were initially processed by cutting and grinding them in liquid nitrogen to create a fine powder. Subsequently, sterile phosphate-buffered saline (PBS) buffer solution was added to the powdered tissue samples. The resulting mixture was then filtered using a 0.22 μm filter (Millipore, USA) to sterilize the supernatant. For RNA extraction, the Fast 200 Total RNA Extremely Fast Extraction Kit (Feijie, Shanghai, China) was applied, following the manufacturer’s instructions. Next, reverse transcription was performed using a Superscript Reverse Transcriptase Reagent Kit (Takara). Then, PCR was performed with the detection primers. The PCR was performed by using KOD FX Neo (Takara) and 10 μM specific primers (S1 Table), with the following cycling program: 5 min at 94°C, 35 cycles of 10 s at 98°C, 20 s at 55°C and adjusted extension at 68°C.

### Virus isolation

The samples were first processed under aseptic conditions using steel balls to ensure thorough homogenization. Subsequently, the homogenized tissues were centrifuged at 12,000 rpm for 10 min at 4°C. The clarified supernatant was then filtered through a 0.22 μm filter to remove any remaining particulate matter and ensure sterility. IPEC-J2 cells were washed twice with PBS and inoculated with the viral suspension. After incubating at 37°C with 5% CO_2_ for 1 hour, the inoculum was removed, and fresh DMEM media containing 2% FBS and 1% penicillin-streptomycin was added. The inoculated cells were then incubated at 37°C for 72 hours and blindly propagated through several passages until cytopathic effects (CPE) were observed.

For virus purification, the supernatant was inoculated into IPEC-J2 cells cultivated in 6-well plates. After 1 hour incubation, the inoculum was removed, and the cells were overlaid with a mixture of 2 × DMEM and 3% low melting agarose. The plates were then incubated at 37 °C in 5% CO_2_. Once CPE was observed after 48 hours, the cells were overlaid with a toluidine blue-containing covering solution to enhance plaque visibility. When plaques were clearly visible, a single plaque was selected and placed into DMEM medium for further culture.

### Electron microscopy analysis

The supernatants of the GETV-infected IPEC-J2 cultures were centrifuged at 3000 × g for 30 min, and the remaining supernatant was then subjected to a second centrifugation at a higher speed of 13,000 × g for another 30 min. After centrifugation, the viral particles were resuspended in the clarified supernatant. To visualize the virus particles, negative staining was performed with 2% phosphotungstic acid (pH 7.0). Finally, the stained samples were examined using transmission electron microscopy (Hitachi, Tokyo, Japan).

### GETV challenge in AG129 mice and neonatal piglets

Sixteen 8-week-old AG129 female mice were purchased from National Institutes for Food and Drug Control (NIFDC, China) and randomly divided into two groups: Group A (subcutaneous injection route, n = 8) and Group B (negative control, n = 8). Physical isolation was implemented between the groups, and strict biosafety measures were taken to prevent cross-infection. Food and water were freely available to the mice throughout the experimental period, without the use of medicinal additives or vaccines. Group A was inoculated with 1 × 10^3^ TCID_50_ each. Group B was the blank group without any treatment. After the challenge, the mice were monitored daily for morbidity or mortality. The infection was considered lethal when the animals reached humane end-points and needed to be euthanized. Surviving mice were euthanized 8 days post challenge. Blood was collected during infection experiment, and a portion of their organs were collected in 4% buffered formalin for histopathological examination. Another portion of the organs was stored in liquid nitrogen for GETV detection by qRT-PCR. To assess the pathogenicity of the isolated GETV in neonatal piglets, seven 2-day-old piglets (negative for PEDV, TGEV, PDCoV, PoRV and GETV) were randomly divided into two groups. Four piglets were intramuscularly injected with 2 mL of GETV-JLy1 (1 × 10^6.5^TCID_50_), and three piglets were inoculated with DMEM as negative-control. The clinical signs and the survival of the piglets were monitored in each group. The infection was considered lethal when the animals reached humane end-points and some tissues were collected for further evaluations by histopathological examination. The animal experiments were carried out in facilities of ABSL-2 level.

### Plasmids and antibodies

The full-length sequence of P46 (P46) and human P46 (hP46) were amplified from the target cells, and then cloned into a pCAGGS or pAcGFP1-C1 or pLVX-IRES-ZsGreen1 vector. Primers used for plasmid construction are listed in S1 Table. Individual GETV gene fused with a HA or Flag tag at the C-terminus was cloned into pCAGGS plasmid vectors. The nsP2 plasmids from different members of Alphavirus (HA-tagged CHIKV nsP2, HA-tagged SINV nsP2, HA-tagged VEEV nsP2, HA-tagged SFV nsP2, and HA-tagged RRV nsP2) and the P46 plasmids from different species (Flag-tagged mouse/horse/goat) were synthesized by SevenBiotech Co., Ltd. Several truncated mutants of nsP2, including HA-tagged nsP2-M1, HA-tagged nsP2-M2, HA-tagged nsP2-M3, HA-tagged nsP2-M4, HA-tagged nsP2-M5, HA-tagged nsP2-M6 were designed and cloned into pCAGGS vectors using the primers in S1 Table. GETV replicon-Luc plasmids and the plasmids of SINV were kindly provided by Prof. Xi Zhou. The subgenomic GETV-Luc replicon (GETV-Luc) was constructed by replacing the viral structural genes (capsid, E3, E2, 6K, and E1) with a luciferase reporter gene under the subgenomic promoter. Scheme of the plasmid used to construct a GETV subgenomic replicon was indicated in S9C Fig. The listed antibodies were used in this study, including the P46 rabbit polyclonal antibody (pAb) from Abcam, Mouse anti-Flag mAb, Mouse anti-HA mAb, Rabbit anti-GFP mAb, and Mouse anti-β-actin mAb were purchased from Sigma, IRDye-conjugated secondary antibody was purchased from Li-Cor Biosciences. The antibodies targeting endogenous host-associated proteins in mice intestinal tissues were trial-size antibodies provided by the companies. The mouse monoclonal antibody against GETV E2 was prepared and preserved in our laboratory.

### Virus infection and cell transfection

IPEC-J2 cells were infected with GETV at the indicated multiplicity of infection (MOI). After 1 h incubation at 37 °C, cells were washed with PBS and then overlaid with complete medium. At specific time points after infection, samples were collected for further analysis. To generate cell-lines stably expression specific constructs, HEK293T cells were co-transfected with the respective pLVX-IRES-ZsGreen1 derived construct, along with pMD2.G and psPAX2. The resulting lentiviral particles were then used to transduce the target cells. Cells were transfected with plasmids using the X-tremeGENE transfection reagent according to the manufacturer’s instructions (Roche, Germany) as previously described [41].

### Generation of P46 knockout IPEC-J2 cells

The target sequence for the P46 gene was inserted into plasmid lentiCRISPRv2 (lentiCRISPRv2-P46). The constructed lentiCRISPRv2-P46 plasmid and lentiCRISPRv2 plasmid were transfected into IPEC-J2 cells. After transfection, the cells are selected under puromycin (1.5 μg/mL) for five days. To obtain clonal cell lines, the selected cells are diluted to 10 cells/mL and then inoculated into 96-well plates. Once colonies have formed, each colony is separately transferred into 48-well plates. Sequencing and Western blotting are conducted to confirm the knockout of the P46 gene (Fig 4A). Genomic DNA is extracted using the TIANamp Genomic DNA Kit (TIANGEN, Beijing). The DNA is then used as a template to amplify an approximate 200 ~ 300 bp region flanking the Protospacer Adjacent Motif (PAM) site. The amplified DNA is gel-extracted, and Sanger sequencing is performed to check for mutations at the target site. The amplification was performed by the primers in S1 Table.

### Immunofluorescence assay (IFA)

IFA was conducted with minor modifications to the previously described protocol [17]. Wild-type (WT) or P46 knockout (P46^-/-^) cells were infected with GETV for a duration of 24 hours. Subsequently, the cells were fixed and immunostained with antibodies targeting the GETV E2 protein for a period of 1 h [42]. Unbound antibodies were then removed, and the cells were further stained with fluorescein isothiocyanate (FITC) or Alexa Fluor 594-labeled goat anti-mouse IgG (Jackson ImmunoResearch) for an additional hour. Nuclei were stained with DAPI (4,6-diamidino-2-phenylindole, Sigma). After thorough washing, the fluorescence signals were visualized using an Olympus inverted fluorescence microscope equipped with a camera.

### Western blotting assay

Western blotting analysis was conducted as previously described [41]. The transfected or virus-infected samples were lysed using RIPA supplemented with a protease inhibitor cocktail (Sigma, USA). Equal amounts of extracts were separated with SDS-PAGE and transferred to methanol-activated polyvinylidene difluoride (PVDF) membranes (Merck Millipore, USA). After blocking, the membranes were incubated with the indicated primary antibodies, and then were incubated with the appropriate secondary antibodies (Li-Cor Biosciences, Lincoln, NE). Finally, the membranes were scanned using an Odyssey instrument (Li-Cor Biosciences) according to the manufacturer’s instructions.

### Co-immunoprecipitation (Co-IP)

The experiment was carried out as previously described [43]. HEK293T cells were co-transfected with the designated plasmids. Subsequently, the cells were harvested and lysed using NP40 lysis buffer containing a protease inhibitor cocktail for 4 h at 4°C. The cellular lysates were centrifuged at 12,000 × g for 10 min to pellet insoluble debris. The supernatants were then incubated with the indicated antibody for 4 h, followed by the addition of protein A/G beads for another 4 h at 4°C to capture the antigen-antibody complex. After centrifugation at 1000 × g for 5 min at 4°C, the supernatants were discarded, and the beads containing the immunocomplexes were washed five times with NP40 wash buffer. Finally, the beads were lysed in lysis buffer to release the bound proteins, and the resulting lysates were used for Western blotting analysis.

### Quantitative reverse transcription–PCR (qRT-PCR)

The qRT-PCR analysis was performed as described previously [44]. Total RNA was extracted from cells and subjected to qRT-PCR. The qRT-PCR reactions were set up using TB Green Premix Ex Taq II (Takara) and 10 μM specific primers (S1 Table), with the following cycling program: 5 min at 94°C, 40 cycles of 10 s at 94°C, 20 s at 55°C and 30 s at 72°C. Relative gene quantification was performed by the method of the cycle threshold (∆∆CT) method [45].

### TCID_50_ assay

The titers of the virus in the cell supernatants were determined using the TCID_50_ assay. Briefly, monolayers of IPEC-J2 cells (for GETV) or HEK293T cells (for SINV) grown in 96-well tissue culture plates (Corning, USA) were incubated with 10-fold serially diluted virus suspensions for 96 h. During this time, CPE were recorded. The TCID_50_ value was then calculated using the Reed–Muench method [43].

### In vitro transcription and transfection

RNA transcription was carried out using the SP6 In Vitro Transcription Kit (Promega, USA) according to the manufactory’s instructions. The in vitro-synthesized RNA transcripts were then transfected into IPEC-J2 cells using Lipofectamine 2000 reagent (Invitrogen, USA).

### Dual-luciferase reporter assay

Cells were co-transfected with RNA transcripts and pRL-TK using Lipofectamine 2000 reagent and incubated for 30 h. According to the manufacturer’s instructions, firefly and Renilla luciferase activities were analyzed using a dual-luciferase reporter assay system (Promega, USA).

### Statistical Analysis

The data are expressed as the mean ± standard deviation (SD) from three independent experiments. All statistical analyses were performed using GraphPad Prism 9. The significance of variability between groups was determined by Student’s t-test. Comparison of survival curves was performed by log-rank test. P value <0.05 was considered significant.

## Supporting information

S1 Fig Percentage identity of the genome-wide sequences among different GETV strains. The genome-wide sequences originated from representative strains were selected and the divergence of nucleotide sequences was analyzed by the DNASTAR software.
(TIF)

S2 Fig Phylogenetic analyses based on the genome sequences of GETV. (TIF)

S3 Fig Pathogenicity of GETV-JLy1 in 2-day-old neonatal piglets. (A) Representative lesions of infected intestinal tract, lung, and spleen. (B) H&E staining of representative lesions in jejunum, ileum and spleen tissues.
(TIF)

S4 Fig Evaluation of endogenous protein expressions in intestinal tissues from GETV- and mock-infected mice by Western blotting analysis. (TIF)

S5 Fig GETV infection downregulates P46 expression and P46 overexpression restricts GETV replication in BHK-21 cells. (A) Overexpression of P46 restricted GETV infection. BHK-21 cells were transfected with plasmids expression P46/Flag or the empty vector for 24 hours, and the cells were infected with GETV and harvested at 24 and 30 hpi. Protein expression was then analyzed by immunoblotting using the indicated antibodies. (B) P46 expression was downregulated upon GETV infection. BHK-21 cells were infected with GETV and harvested at 24 and 30 hpi. The levels of P46 expressions were analyzed by Western blotting. (C) BHK-21 cells were transfected with plasmids encoding P46/Flag and nsP2/HA followed by treatments with MG132 or DMSO. Immunoblotting was performed using antibodies specific to HA tag, Flag tag, or β-actin.
(TIF)

S6 Fig Percentage identity of *P46* encoded protein sequences among various species. P46 genes were selected from various species and the divergence of amino acids was analyzed by the DNASTAR software.
(TIF)

S7 Fig P46 overexpression inhibits SINV replication. (A) Overexpression of P46 restricted SINV infection. HEK293T cells were transiently transfected with plasmids expression P46/Flag or the empty vector for 24 hours, and the cells were infected with SINV-GFP. At 24 and 30 hpi, the cells were collected for Western blotting (A). Cells were transfected with plasmids expression P46/Flag followed by SINV infection for another 24 hours. At 24 hpi, the cells were collected for immunofluorescence staining (B and C), and TCID_50_ assay (D). *, *P* < 0.05, **, *P* < 0.01. The *P* value was calculated using Student’s t-tests.
(TIF)

S8 Fig Construction of P46 KO cell line. Sequences analysis of P46 KO cell lines using DNA sequencing. The insert location was indicated by the red box.
(TIF)

S9 Fig P46 mediates GETV RNA synthesis. (A and B) Effect of P46 knockout on GETV entry. WT and P46^-/-^ cells were infected with GETV for 2 h at 4°C. Unbound viral particles were removed by washing with ice-cold PBS for five times. For further internalization assay, the cells were transferred to 37°C to facilitate viral internalization. At 1 h after infection, the cells were washed with ice-cold PBS to remove noninternalized viruses. Total RNA was extracted, and was then analyzed by qRT-PCR. (C) Schematic diagram of the subgenomic GETV-Luc replicon. (D) GETV replicon RNA-Luc was synthesized by using SP6 *In Vitro* Transcription Kit, and then transfected along with pRL-TK into WT and P46^-/-^ cells, respectively. After 30 hours of transfection, samples were harvested for dual-luciferase assay. The results represent three independent experiments (the means ± SD). ns, no significant, **, *P* < 0.01. The *P* value was calculated using Student’s t-tests.
(TIF)

S10 Fig SINV infection downregulated P46 expression. HEK293T cells were infected with SINV-GFP and harvested at 24 and 30 hpi. Protein levels of P46, GFP, and β-actin were analyzed by Western blotting.
(TIF)

S11 Fig Screening GETV-encoded proteins inhibiting P46 expression. HEK293T cells were co-transfected with plasmids encoding P46/Flag and expression plasmids encoding HA-tagged GETV proteins. After 24 hours of transfection, immunoblotting was performed using antibodies against the HA tag, Flag tag, or β-actin.
(TIF)

S12 Fig Percentage identity of the nsP2 amino acid sequences among different alphaviruses. The sequences originated from GETV, CHIKV, RRV, SFV, SINV and VEEV were selected and the divergence of amino acids was analyzed by the DNASTAR software.
(TIF)

S13 Fig Screening SFV and SINV-encoded proteins inhibiting P46 expression. HEK293T cells were co-transfected with plasmids encoding P46/HA and expression plasmids encoding Flag-tagged individual SFV (A) and SINV (B) gene. After 24 hours of transfection, immunoblotting was performed using antibodies against the HA tag, Flag tag, or β-actin.
(TIF)

S1 Table Primers used in this study. (DOCX)

S1 Raw Image Raw data from all the images in the study. The raw data follow the same order as presented in figures and supporting information.
(PDF)
