# Supplementary material for: Potential threats of porcine Getah virus outbreaks and its specific antagonism of host antiviral response through proteasome-dependent degradation of P46 by nsP2
Source: PLoS Pathog. 2026 Aug 26;22(8):e1014447. doi: 10.1371/journal.ppat.1014447 (PMC13515021; doi:10.1371/journal.ppat.1014447)
Supplement: Supplementary file 1 [file ppat.1014447.s015.pdf]

Fig. 1

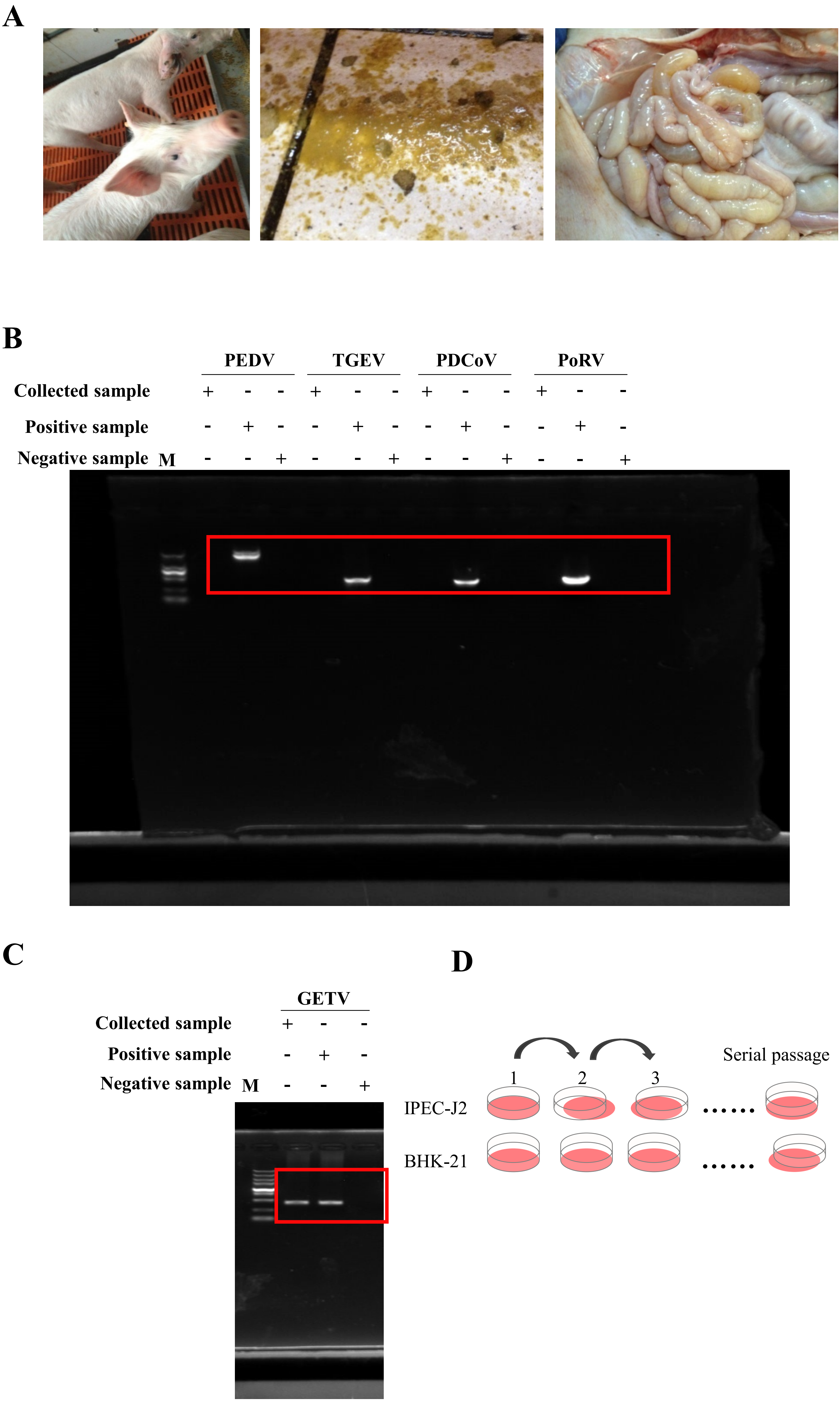
A
B
PEDV
TGEV
PDCoV
PoRV
Collected sample
Positive sample
Negative sample
M
C
GETV
Collected sample
Positive sample
Negative sample
M
D
Serial passage
1
2
3
IPEC-J2
BHK-21

E

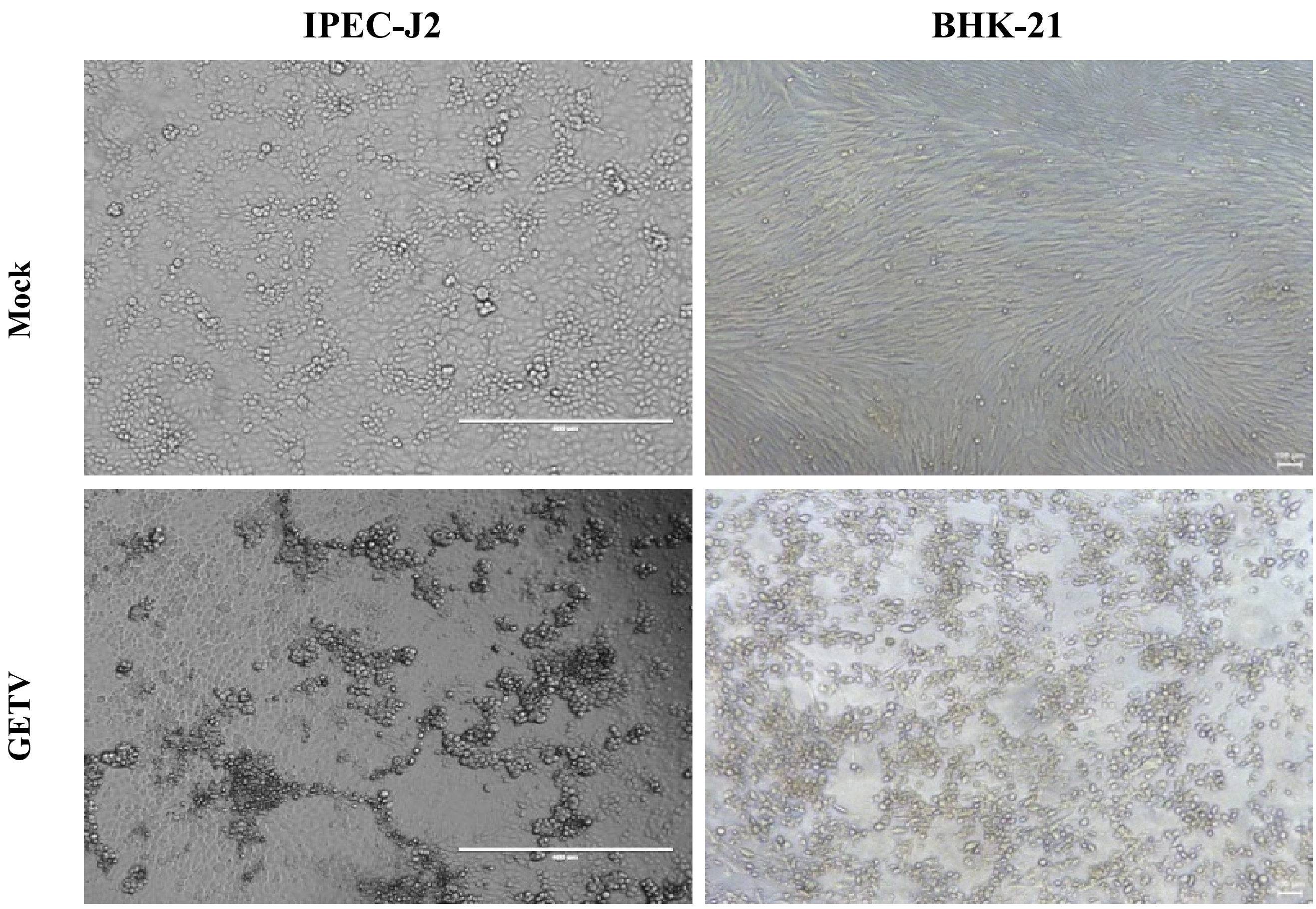

F

Passage 1	+	-	-	-	-	-	-
Passage 2	-	+	-	-	-	-	-
Passage 3	-	-	+	-	-	-	-
Passage 4	-	-	-	+	-	-	-
Passage 5	-	-	-	-	+	-	-
Passage 6	-	-	-	-	-	+	-
Negative Control	-	-	-	-	-	-	+

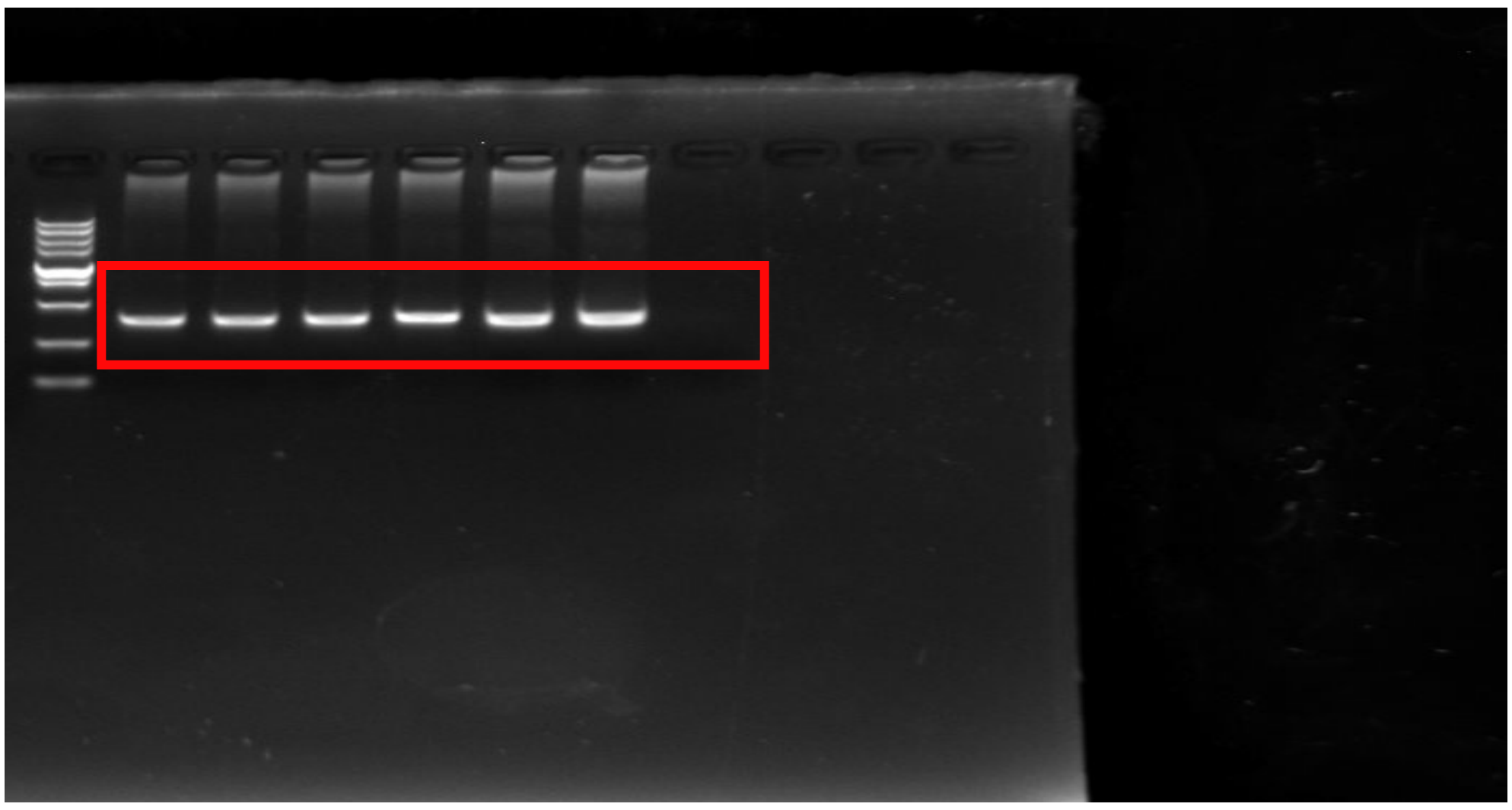

G

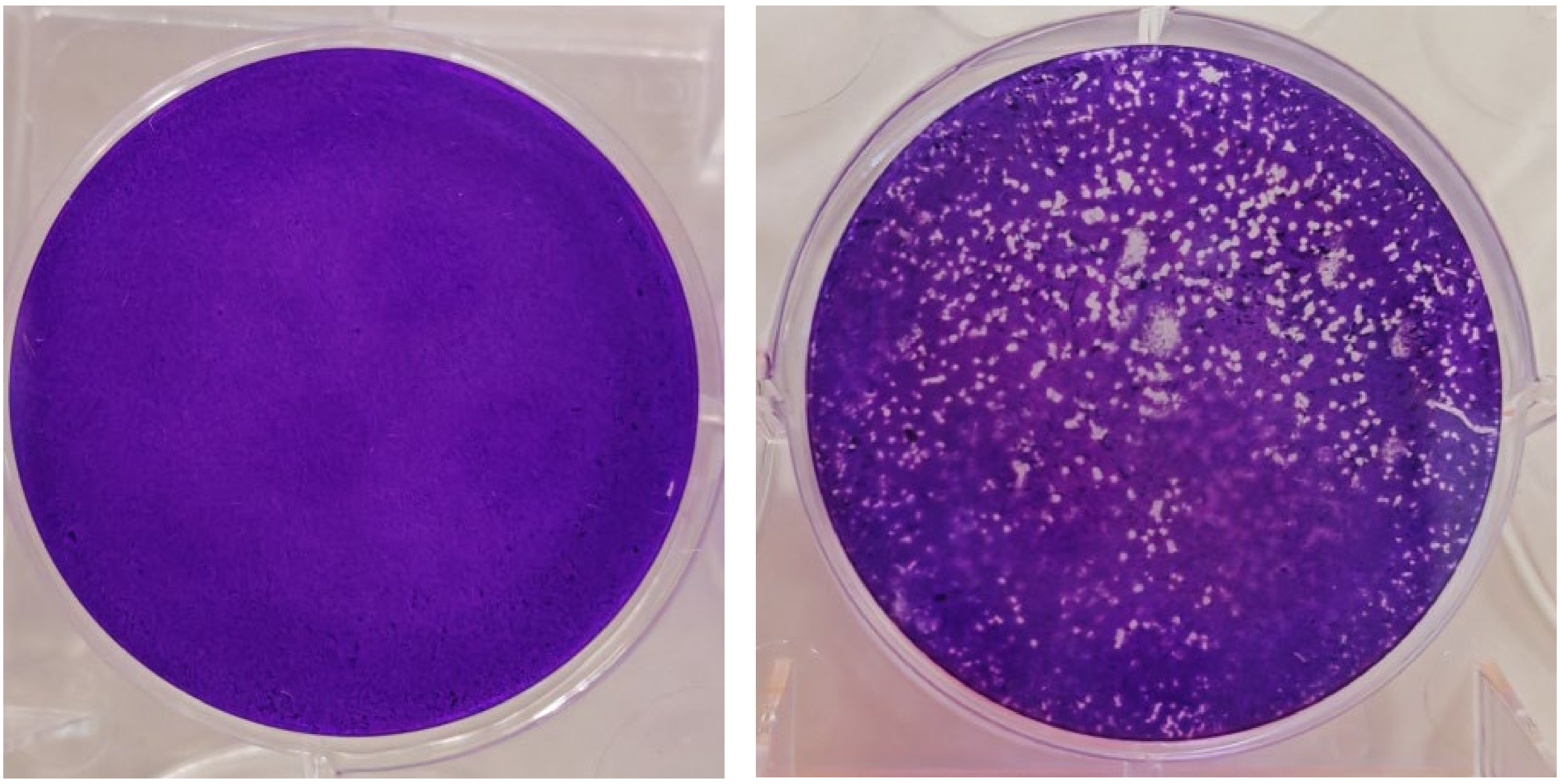

H

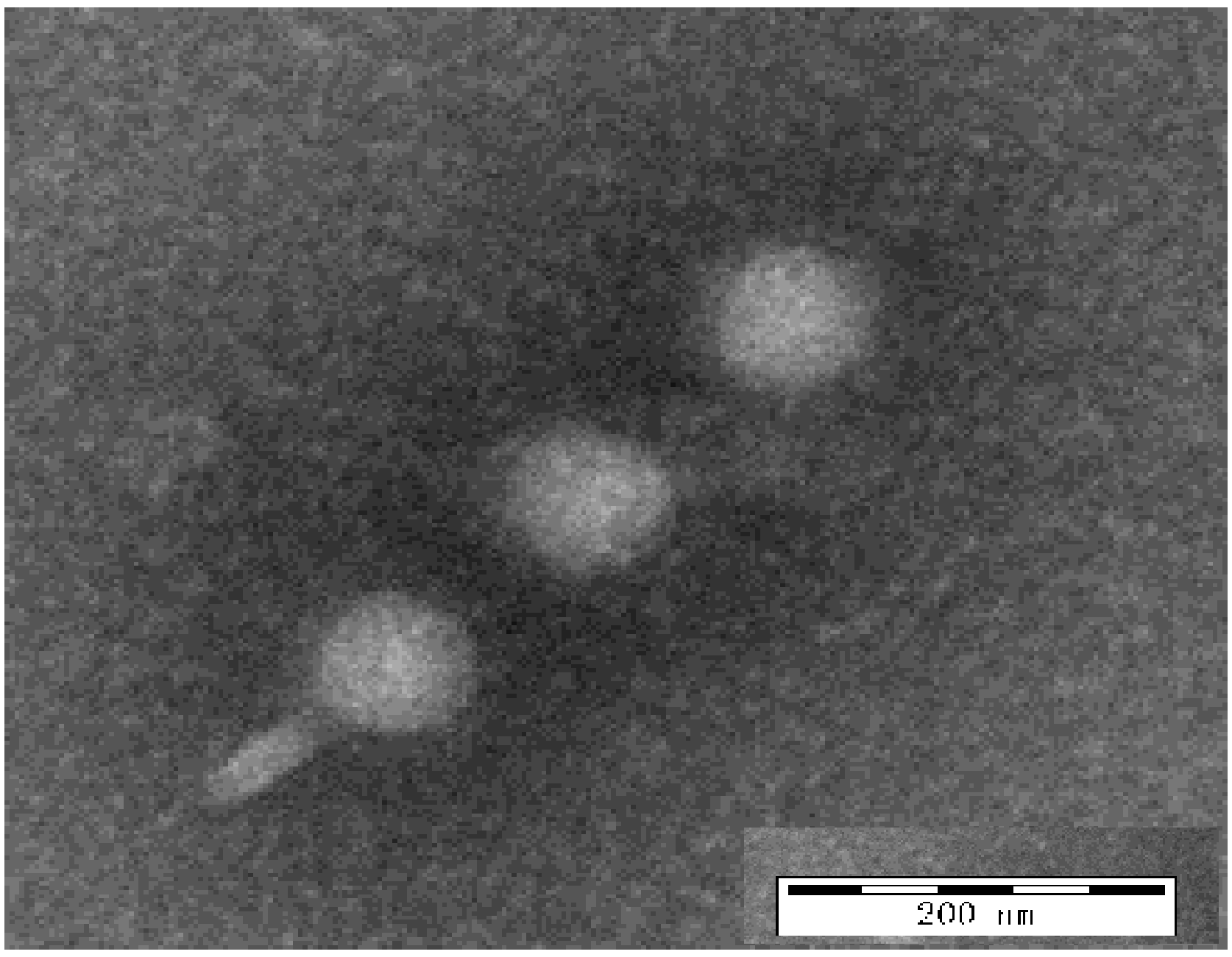

I

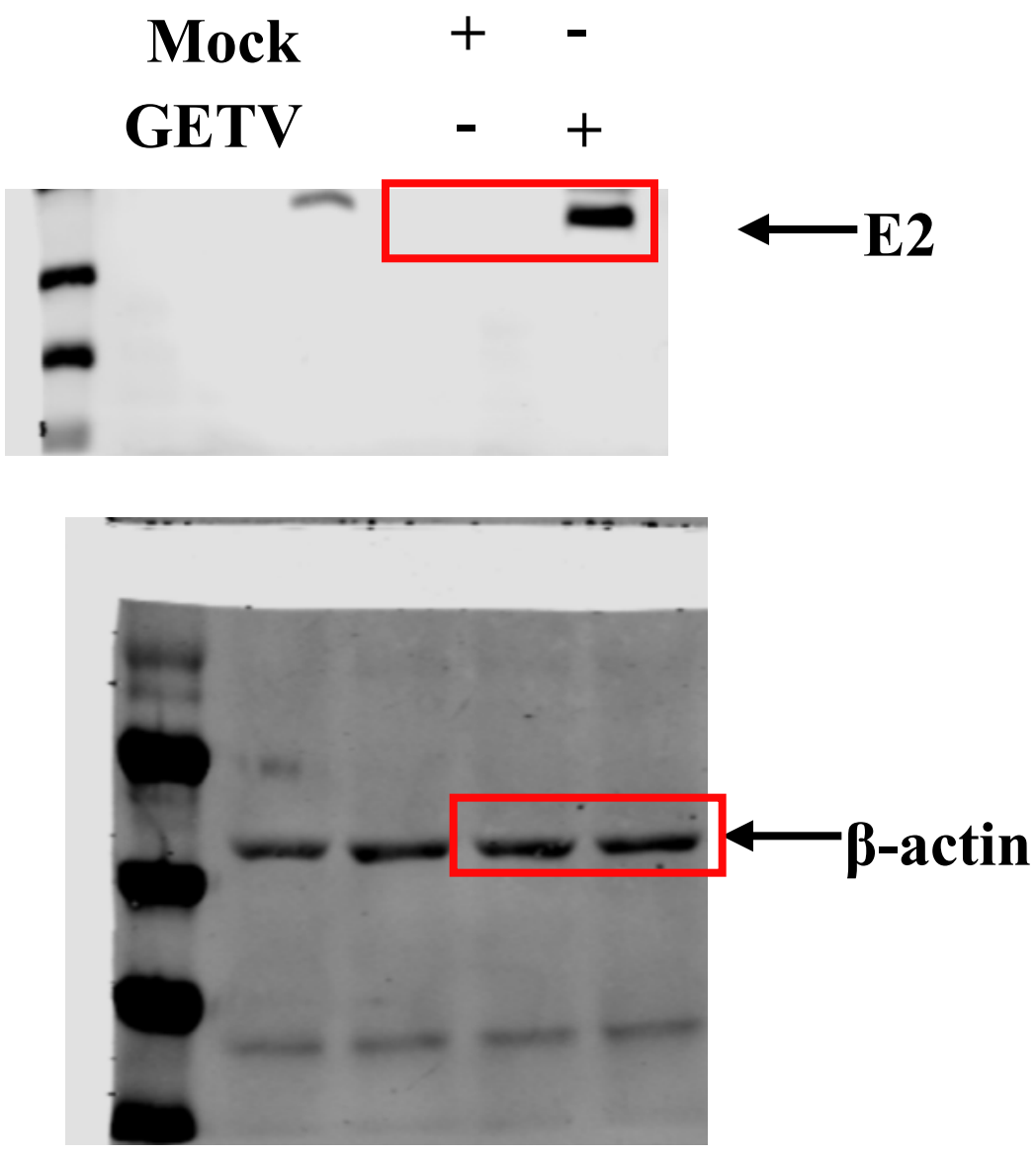

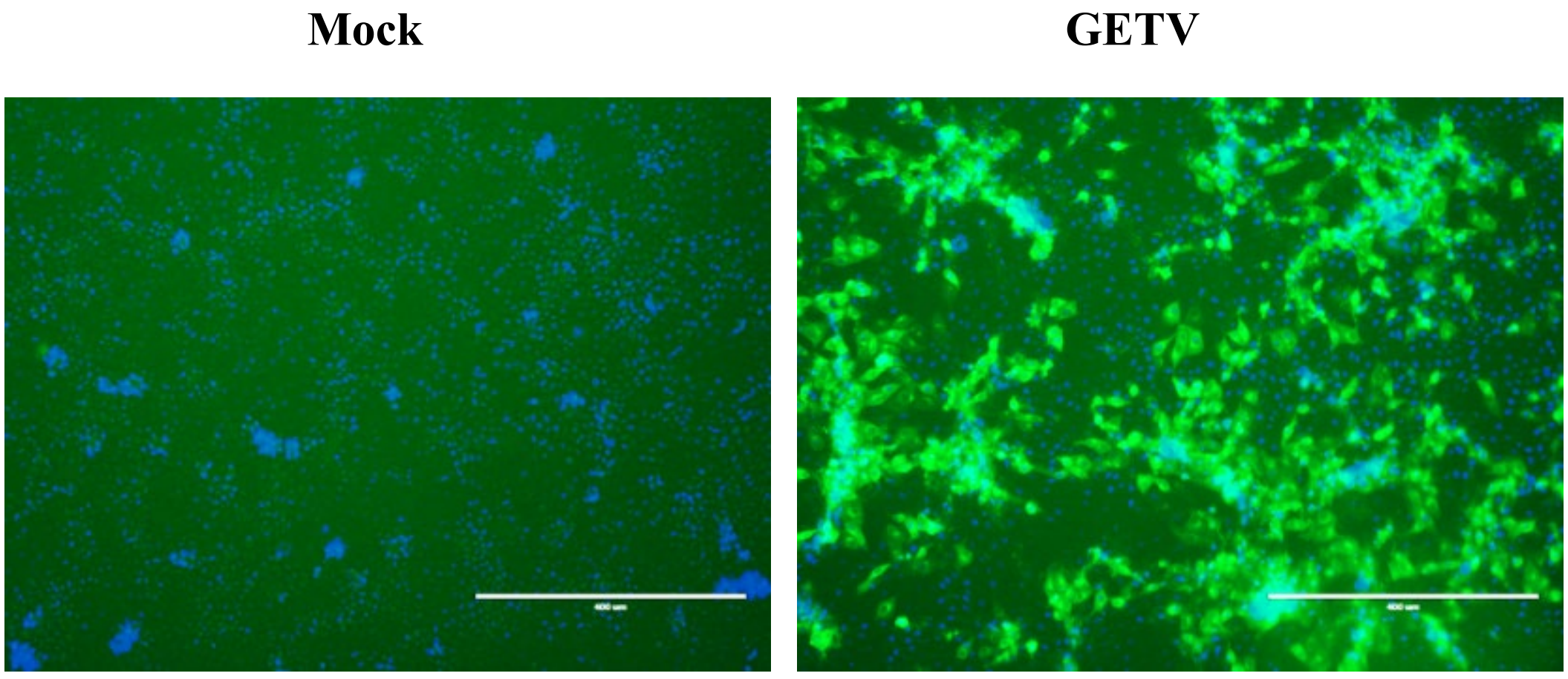

J

	repeat 1#	repeat 2#	repeat 3#	mean	SD
12h	2.43	2.67	2.51	2.5366667	0.122202
24h	4.83	5.67	5.57	5.3566667	0.4588391
36h	5.91	6.15	6.59	6.2166667	0.3448671
48h	5.79	5.54	5.21	5.5133333	0.2909181
60h	4.3	4.7	5.32	4.7733333	0.513939
72h	4.23	4.11	4.89	4.41	0.42

Fig. 2

A

	survival(No)	death (No)
1 day	0	0
2 day	0	0
3 day	0	1
3 day	0	1
4 day	0	1
4 day	0	1
4 day	0	1
4 day	0	1
5 day	0	1
5 day	0	1
6 day	0	
7 day	0	

B

	repeat 1#	repeat 2#	repeat 3#	repeat 4#	repeat 5#	repeat 6#	repeat 7#	repeat 8#	mean	SD
1 day	3.14	3.78	3.09	2.77	4.31	3.09	2.46	3.69	3.3366667	0.384751
3 day	4.04	5.17	6.89	5.29	7.11	5.32			5.3666667	1.4351423
5 day	5.87	4.43							3.4333333	1.0182338

C

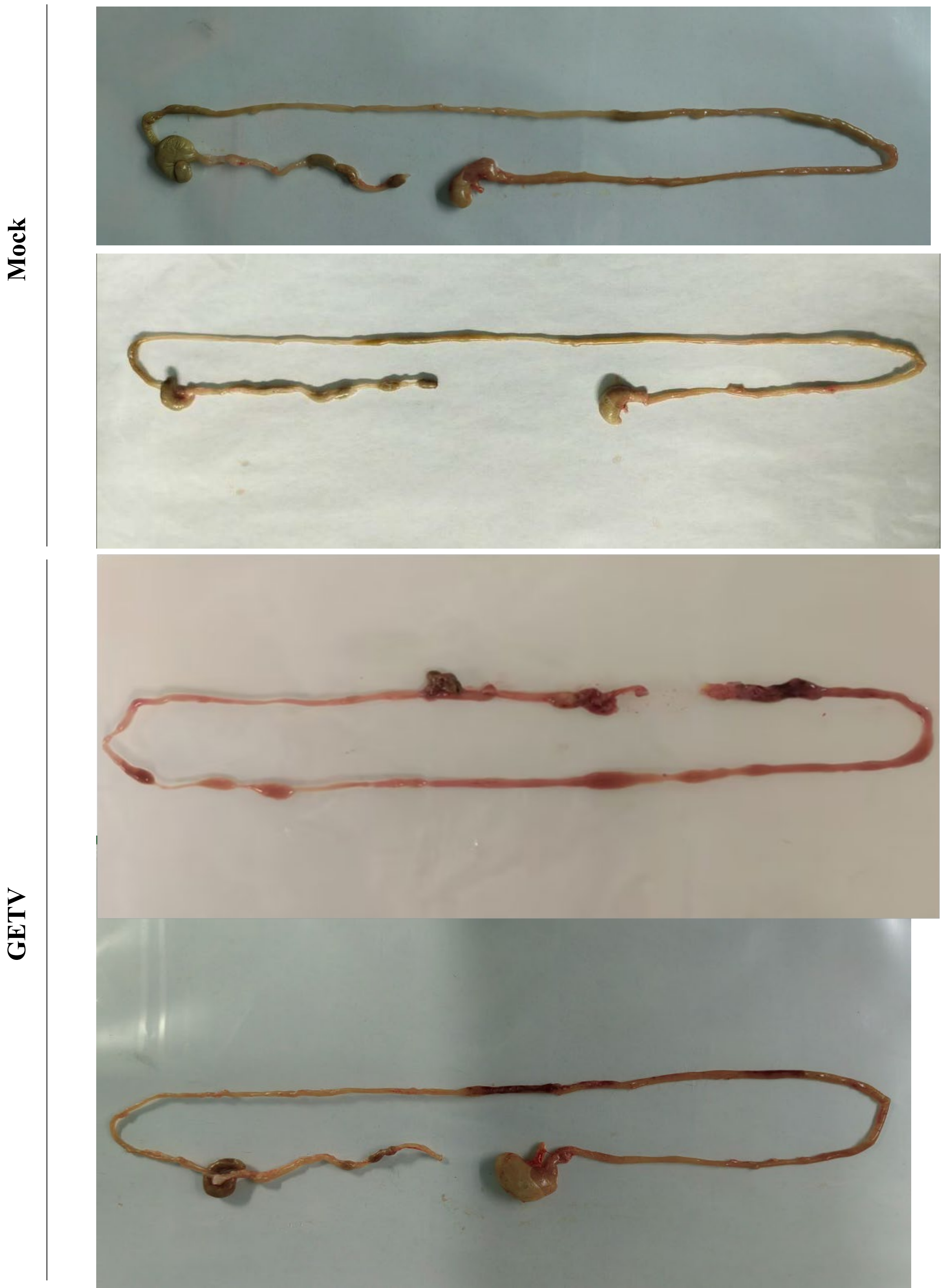

D

	repeat 1#	repeat 2#	repeat 3#	repeat 4#	repeat 5#	repeat 6#	repeat 7#	repeat 8#	mean	SD
Mock-Jejunum	0	0	0	0	0	0	0	0	0	0
GETV-Jejunum	6.091	4.163	5.173	3.175	4.318	5.853	3.853	4.679	5.1423333	0.9643658

	repeat 1#	repeat 2#	repeat 3#	repeat 4#	repeat 5#	repeat 6#	repeat 7#	repeat 8#	mean	SD
Mock-Ileum	0	0	0	0	0	0	0	0		0
GETV-Ileum	3.073	6.213	6.199	5.596	6.723	6.541	4.538	5.537	5.1616667	1.8088519

E

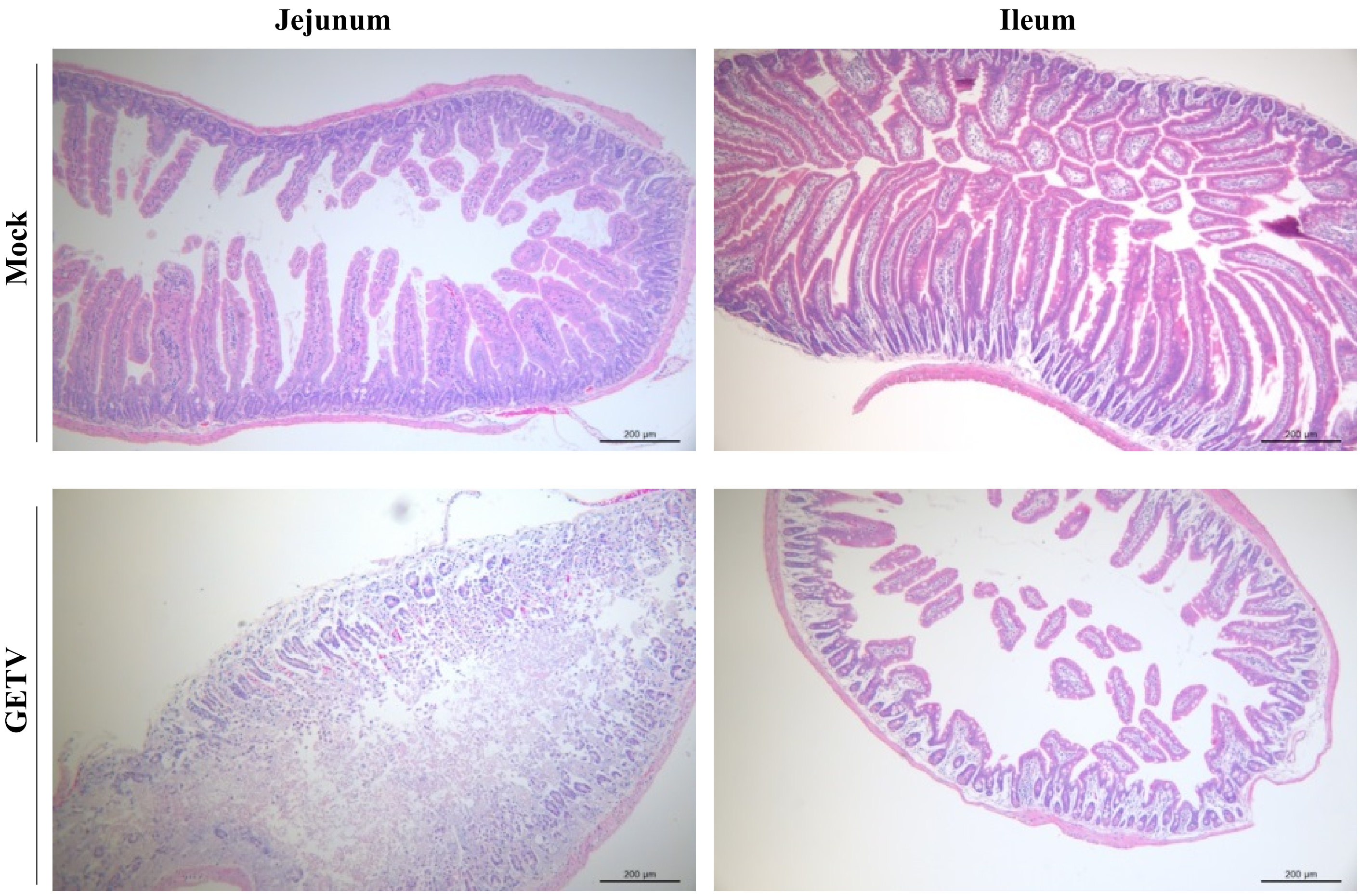

F

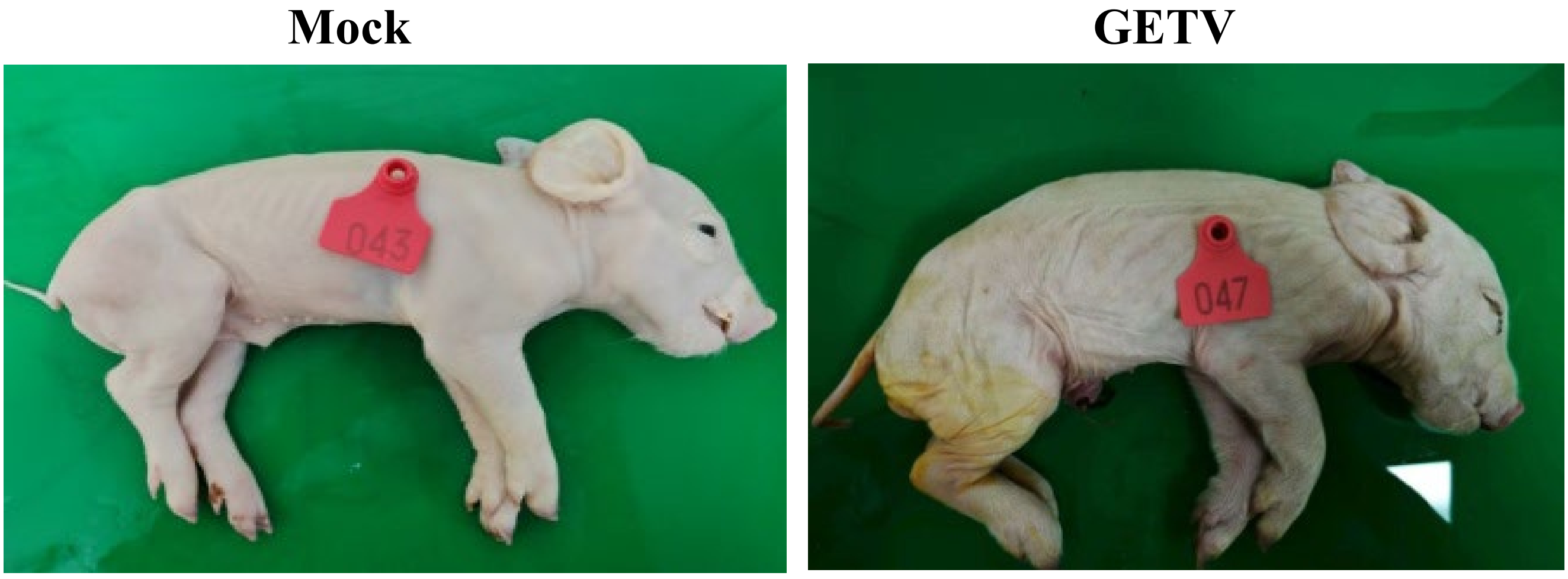

G

	survival(No)	death (No)
20 h	0	1
24 h	0	1
28 h	0	1
32 h	0	1
36 h	0	
40 h	0	
44 h	0	
48 h	0	

Fig. 3

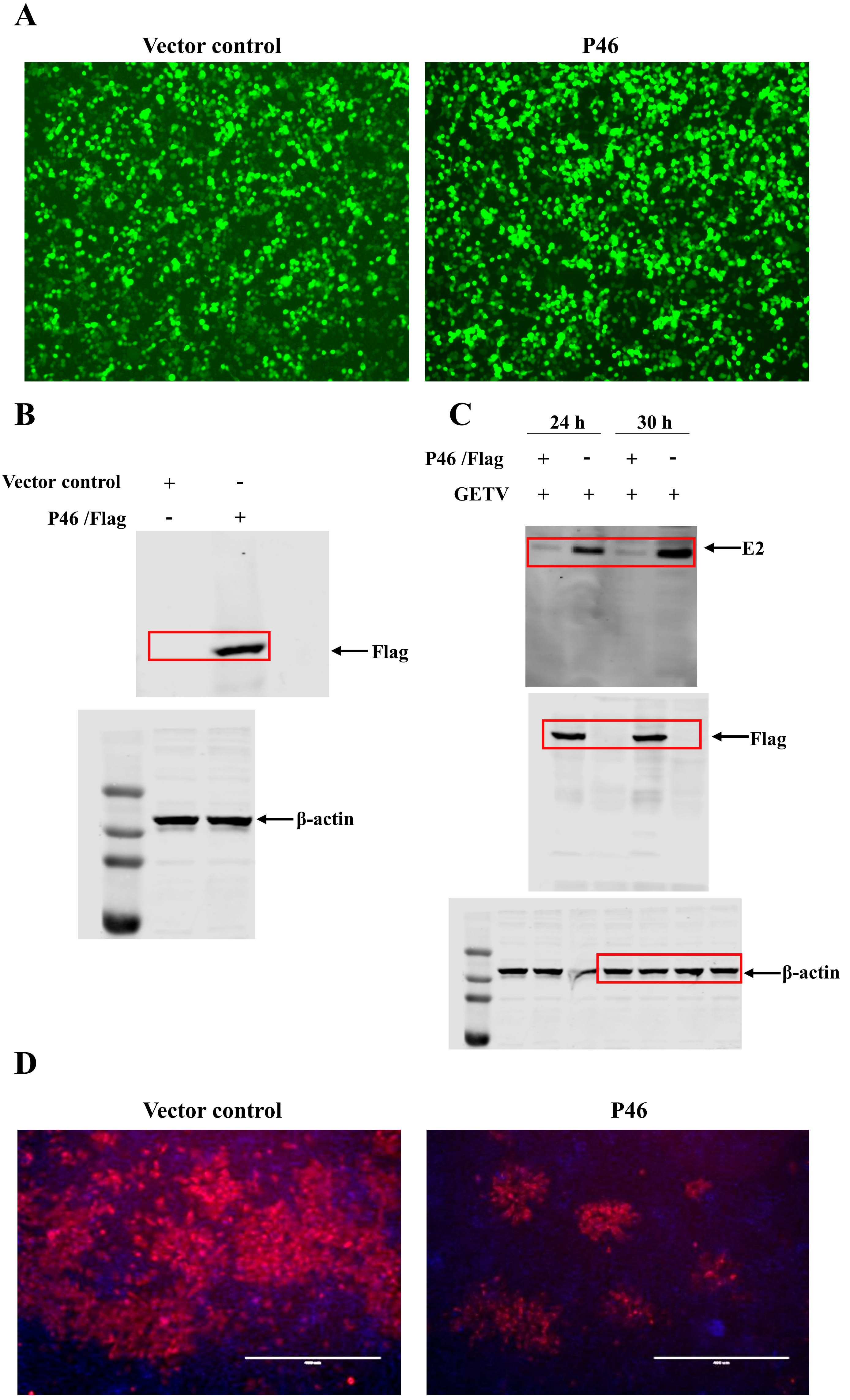
A
Vector control
P46
B
Vector control + -
P46 /Flag - +
Flag
β-actin
C
24 h
30 h
P46 /Flag + - + -
GETV + + + +
E2
Flag
β-actin
D
Vector control
P46

E

	repeat 1#	repeat 2#	repeat 3#	mean	SD
Vector control	75	60	70	68.333333	7.6376262
P46	25	30	11	22	9.8488578

F

	repeat 1#	repeat 2#	repeat 3#	mean	SD
Vector control	1	1	1	1	0
P46	0.282901	0.394378	0.510903	0.3960607	0.1140103

G

	repeat 1#	repeat 2#	repeat 3#	mean	SD
Vector control	5.3	5.2	5.4	5.3	0.1
P46	4.1	4.6	4.3	4.3333333	0.2516611

H

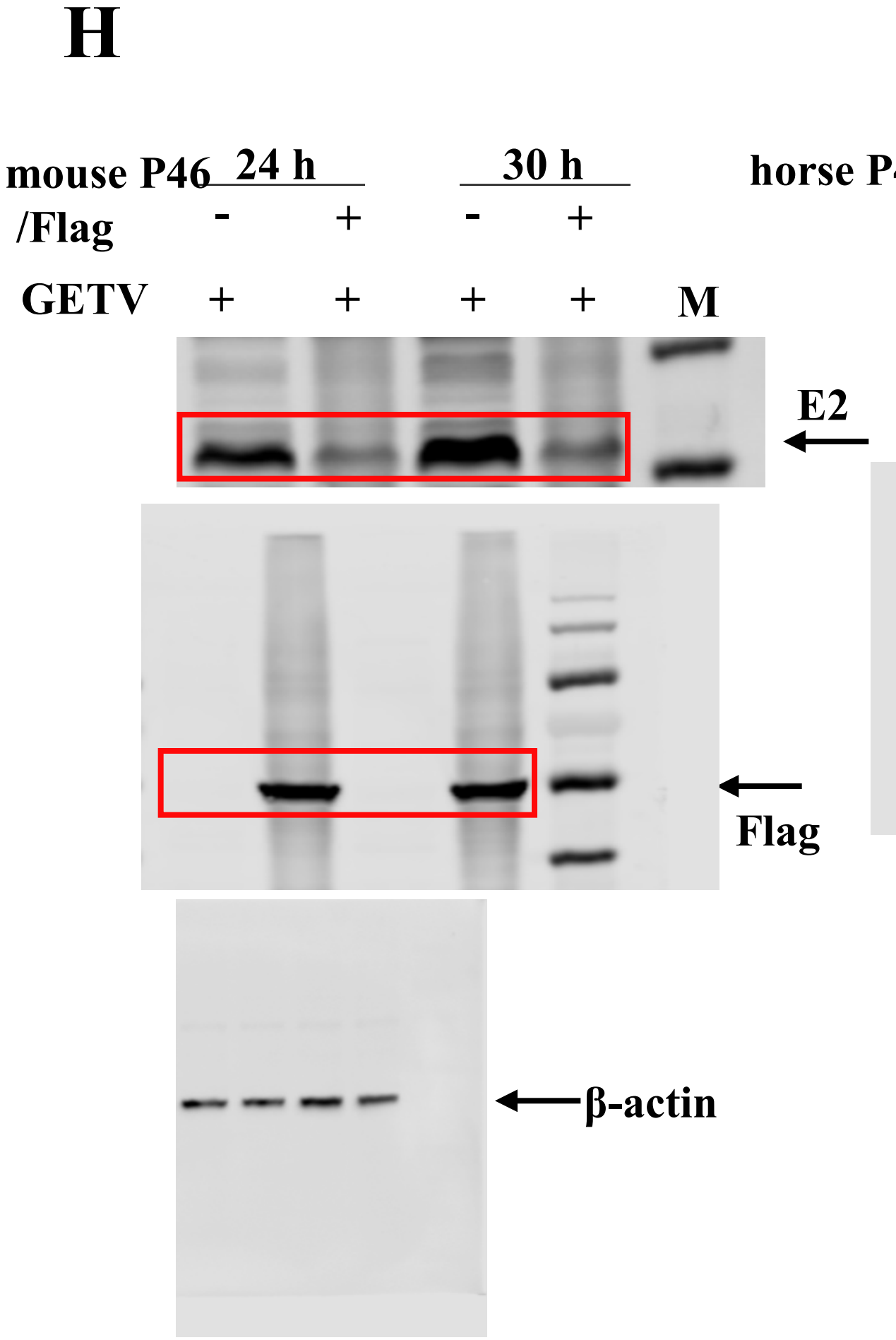

I

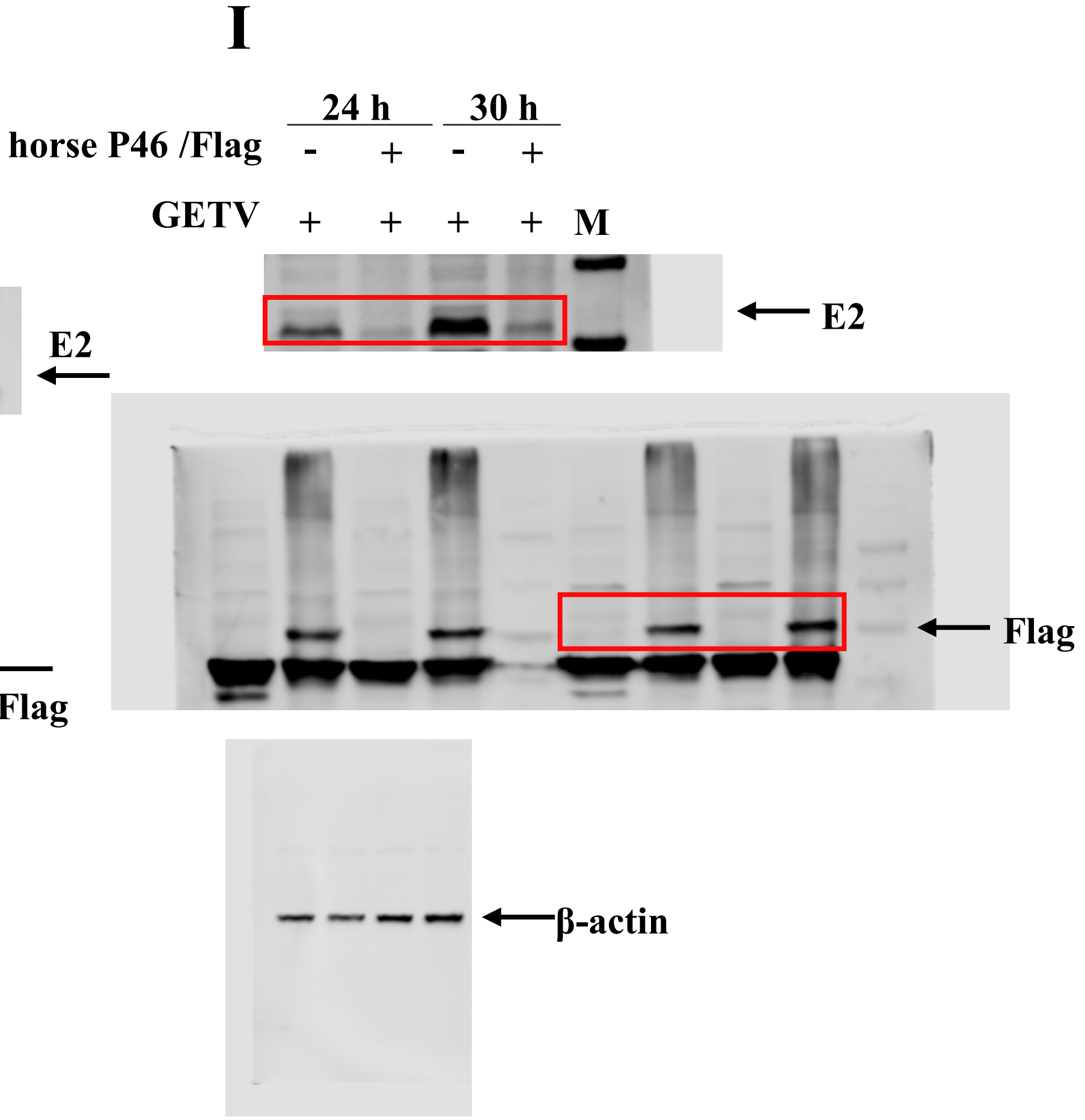

J

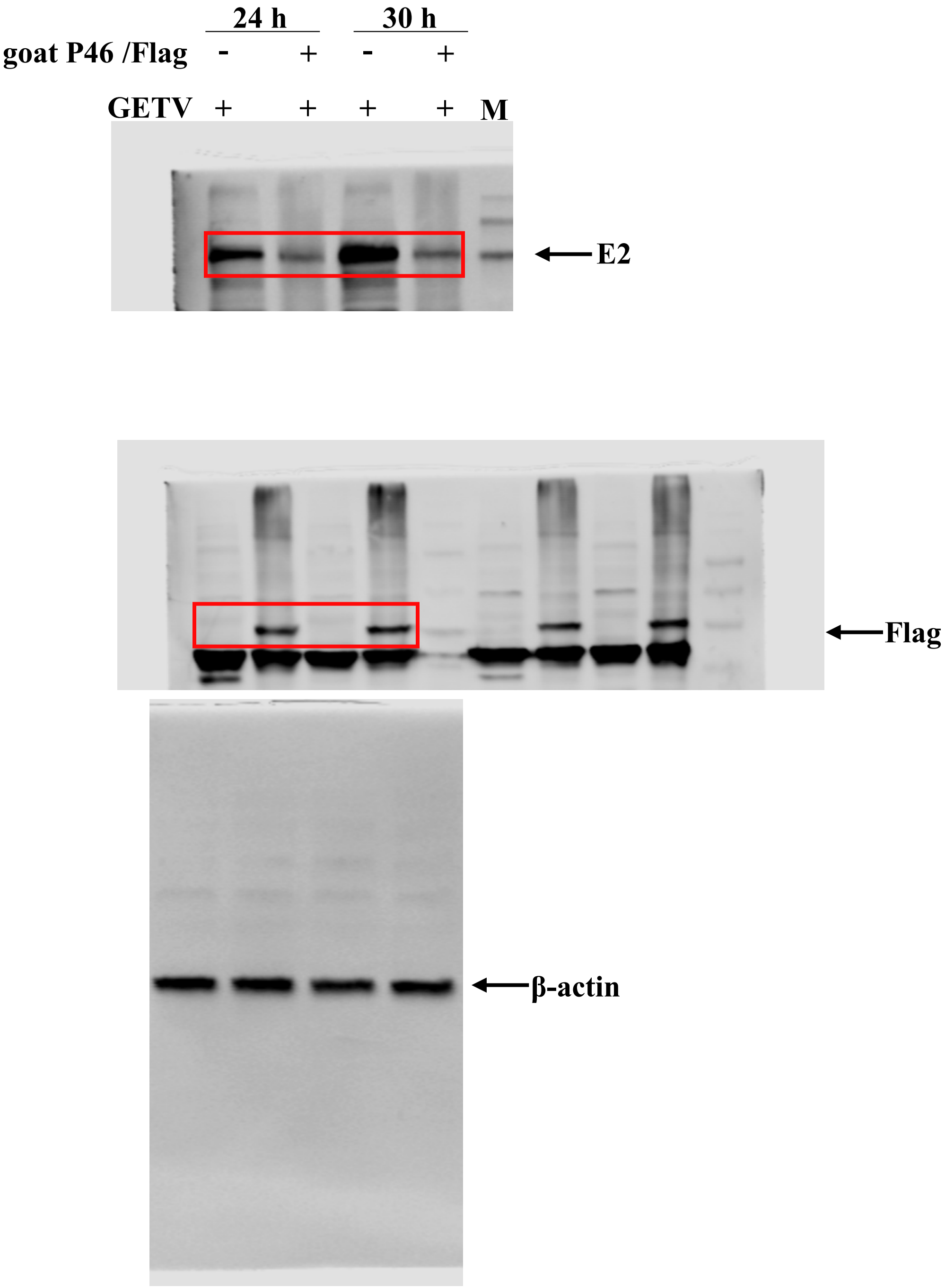

Fig. 4

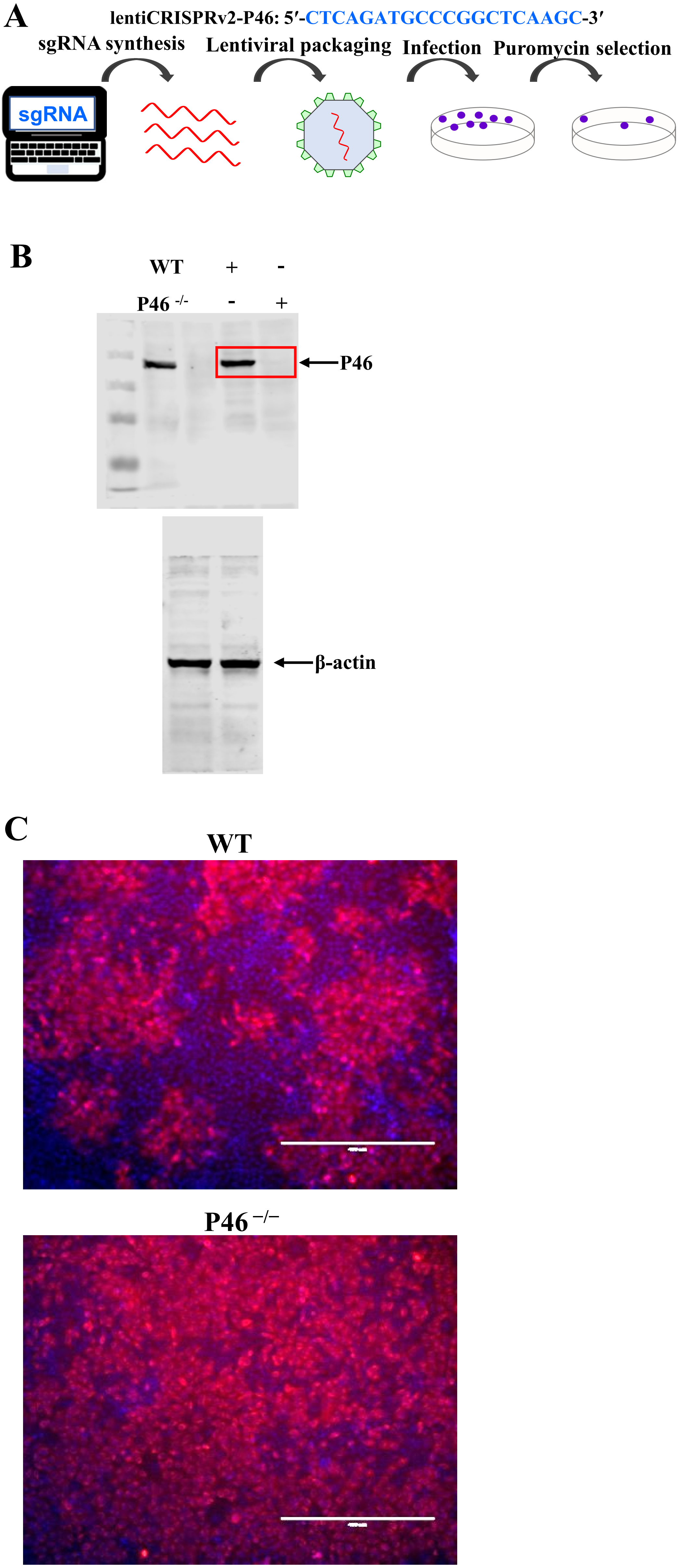
A
lentiCRISPRv2-P46: 5′-CTCAGATGCCCGGCTCAAGC-3′
sgRNA synthesis
Lentiviral packaging
Infection
Puromycin selection
sgRNA
B
WT + -
P46 -/- - +
P46
β-actin
C
WT
P46 −/−

D

	repeat 1#	repeat 2#	repeat 3#	mean	SD
WT	69	65	50	61.333333	10.016653
P46-/-	95	95	90	93.333333	2.8867513

E

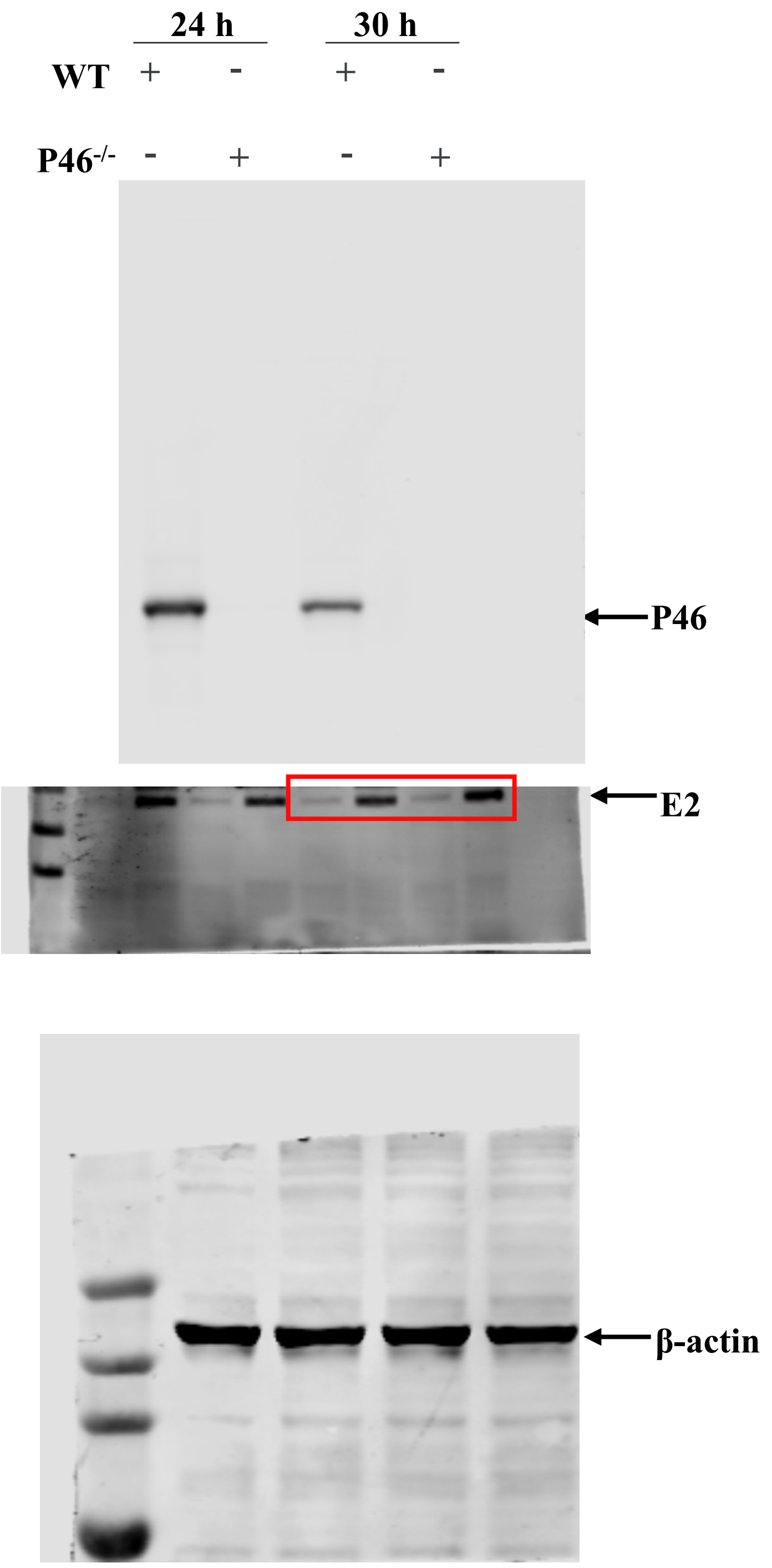

F

	repeat 1#	repeat 2#	repeat 3#	mean	SD
WT	1	1	1	1	0
P46-/-	9.092156	8.590322	7.990334	8.557604	0.5516392

G

	repeat 1#	repeat 2#	repeat 3#	mean	SD
WT	5.2	5.1	4.9	5.0666667	0.1527525
P46-/-	6.1	6.1	6.4	6.2	0.1732051

H

WT

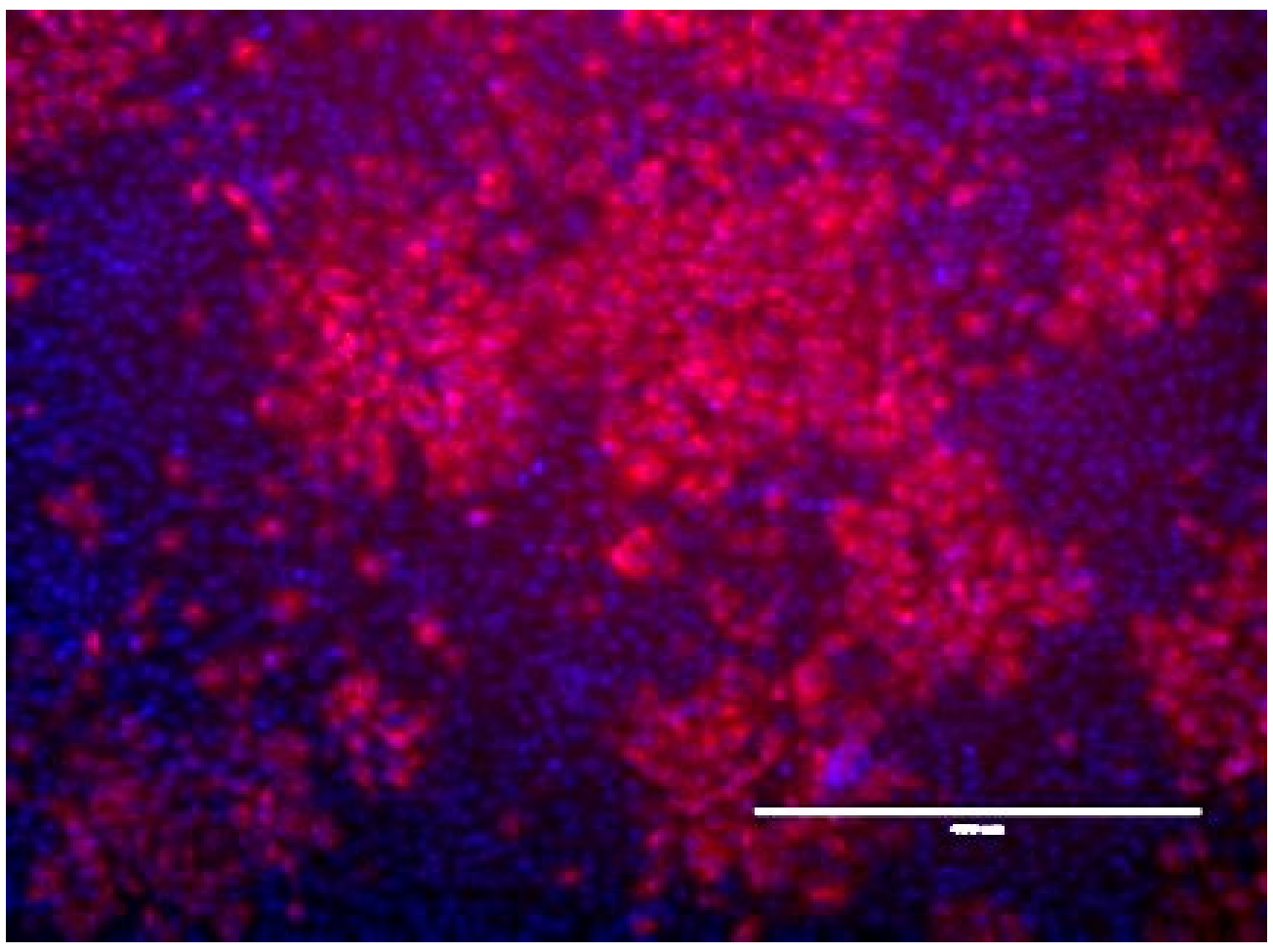

P46$^{-/-}$

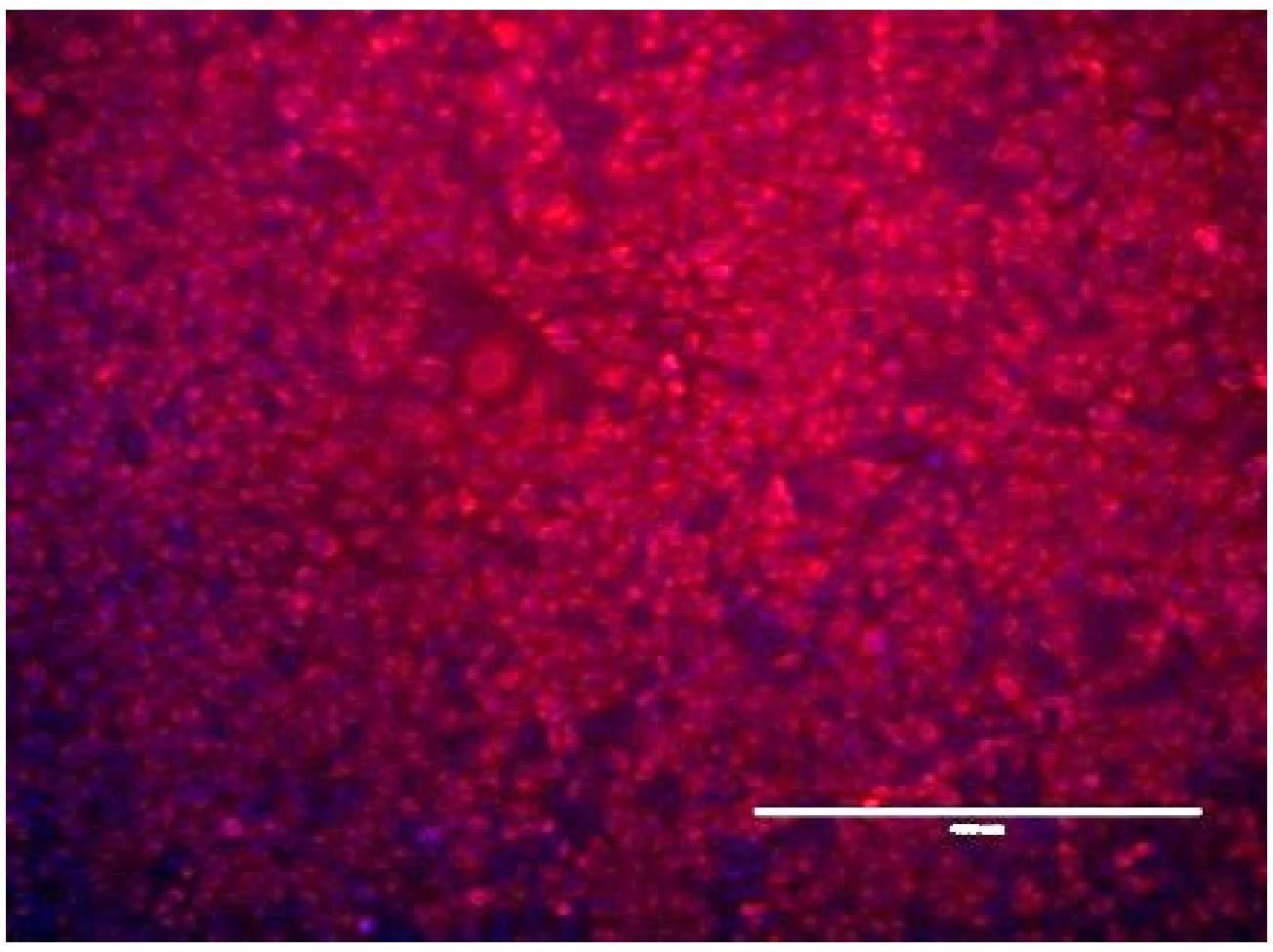

P46$^{-/-}$+P46

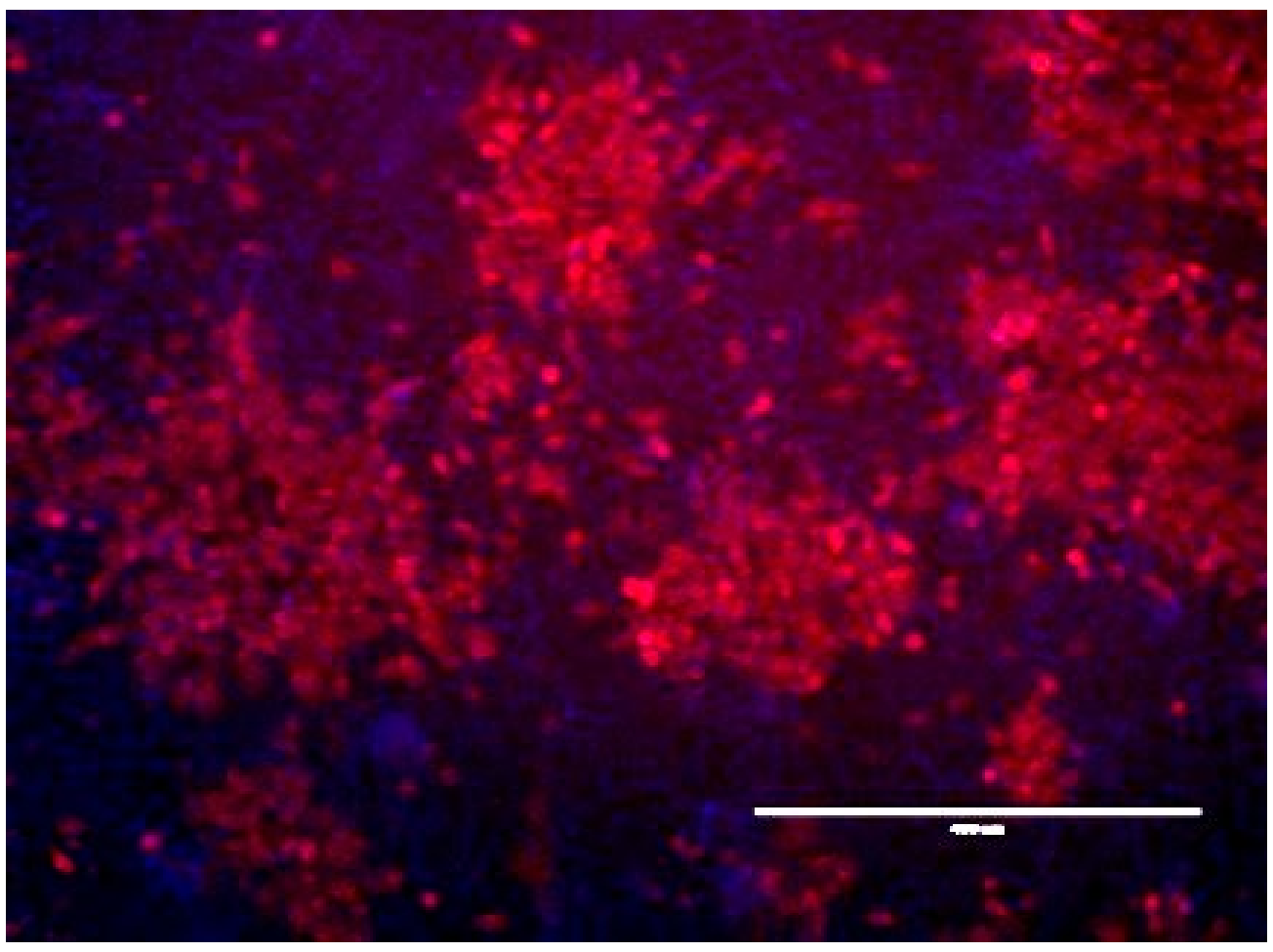

P46$^{-/-}$+Vector con

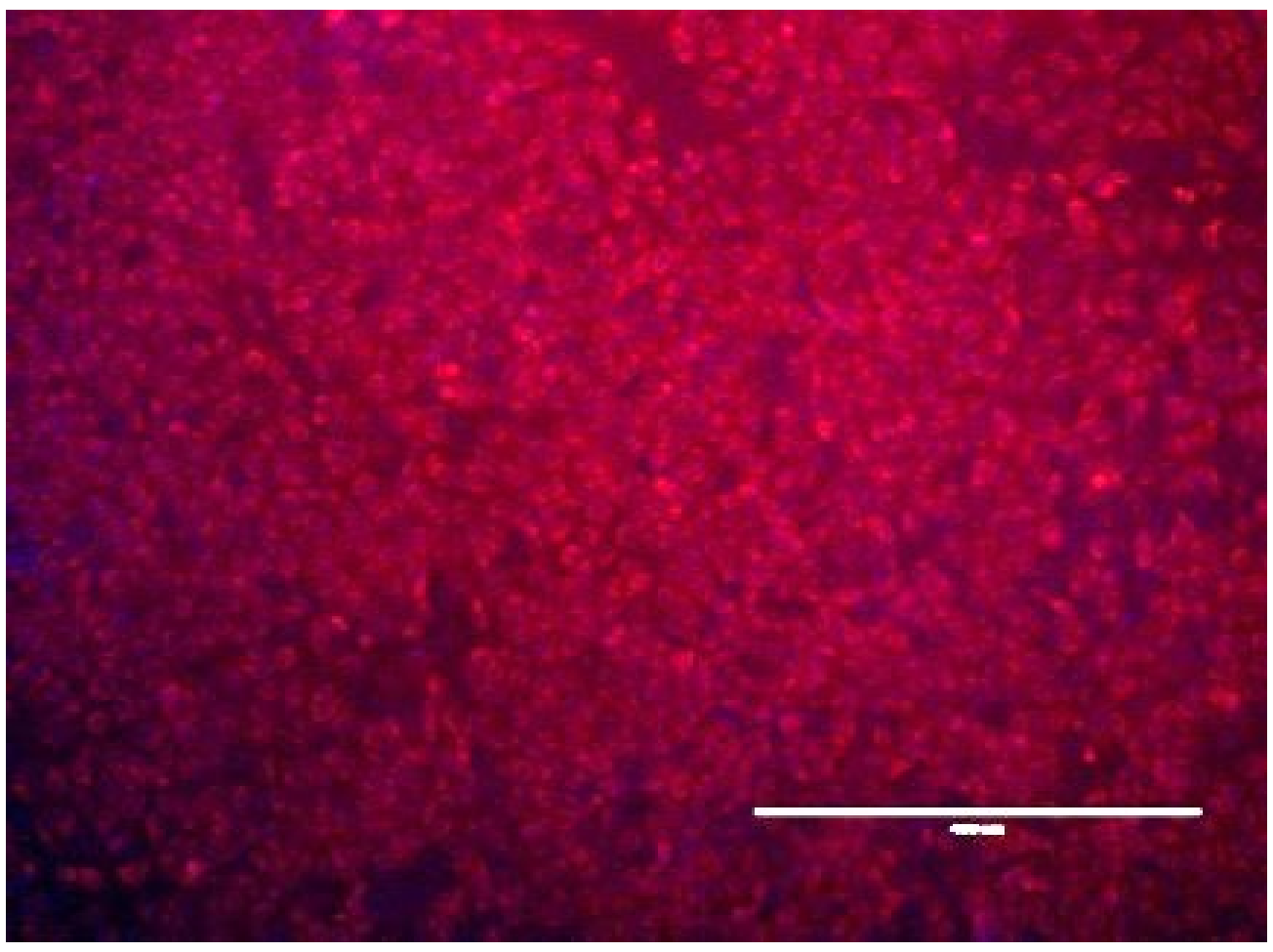

I

	repeat 1#	repeat 2#	repeat 3#	mean	SD
WT	50	49	60	53	6.0827625
P46-/-	83	90	91	88	4.3588989
P46-/-+P46	51	57	30	46	14.177447
P46-/-+Vector control	96	90	88	91.333333	4.163332

J

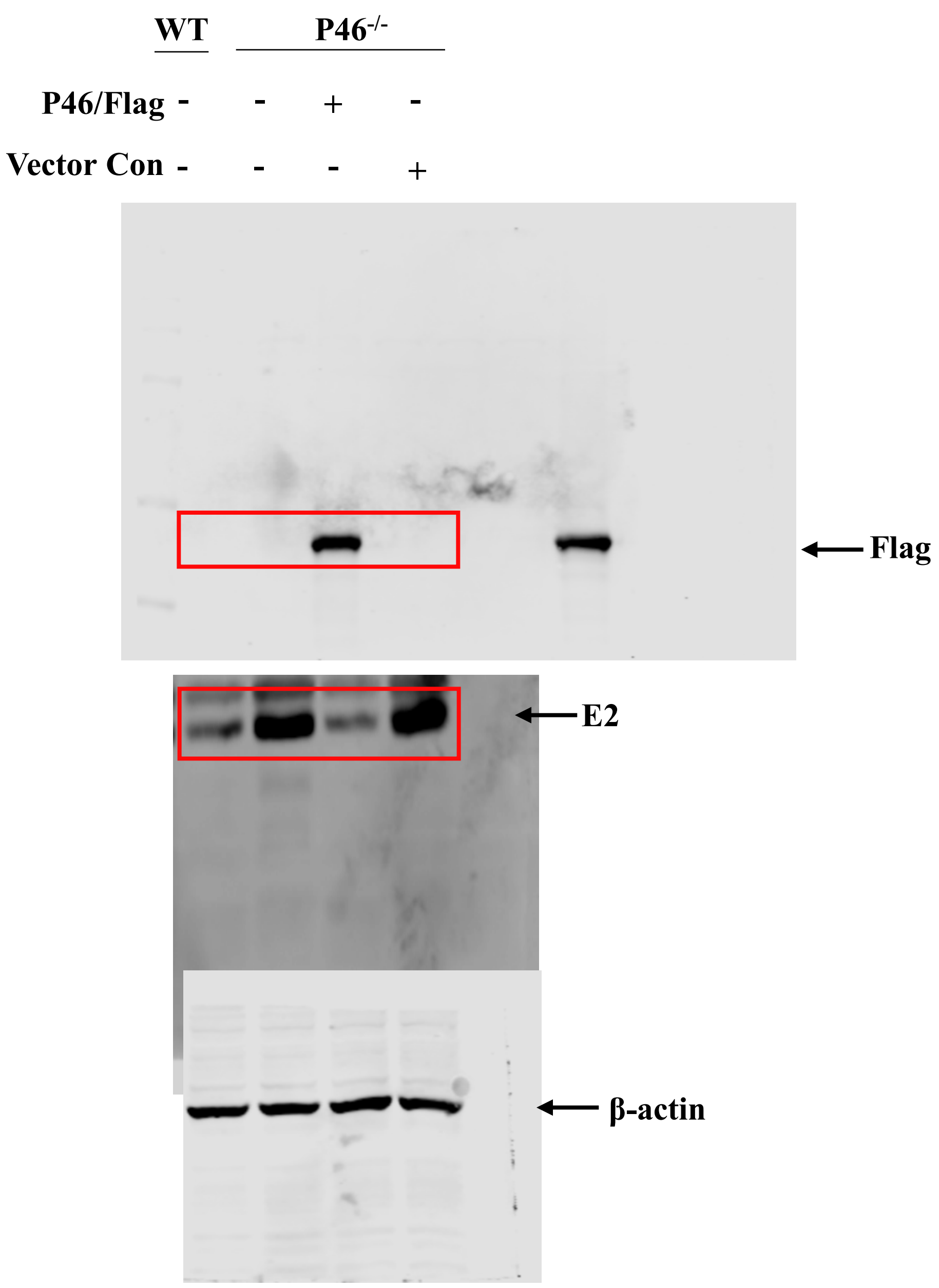

K

	repeat 1#	repeat 2#	repeat 3#	mean	SD
WT	1	1	1	1	0
P46-/-	8.309675	9.240115	7.512001	8.3539303	0.8649066
P46-/-+P46	1.129526	0.698145	0.889431	0.9057007	0.2161502
P46-/-+Vector control	7.319959	7.998976	8.349962	7.8896323	0.523635

L

	repeat 1#	repeat 2#	repeat 3#	mean	SD
WT	5	5.3	4.8	5.0333333	0.2516611
P46-/-	6.3	6.1	6.5	6.3	0.2
P46-/-+P46	4.9	4.6	5.3	4.9333333	0.3511885
P46-/-+Vector control	6.3	6.4	6	6.2333333	0.2081666

Fig. 5

A

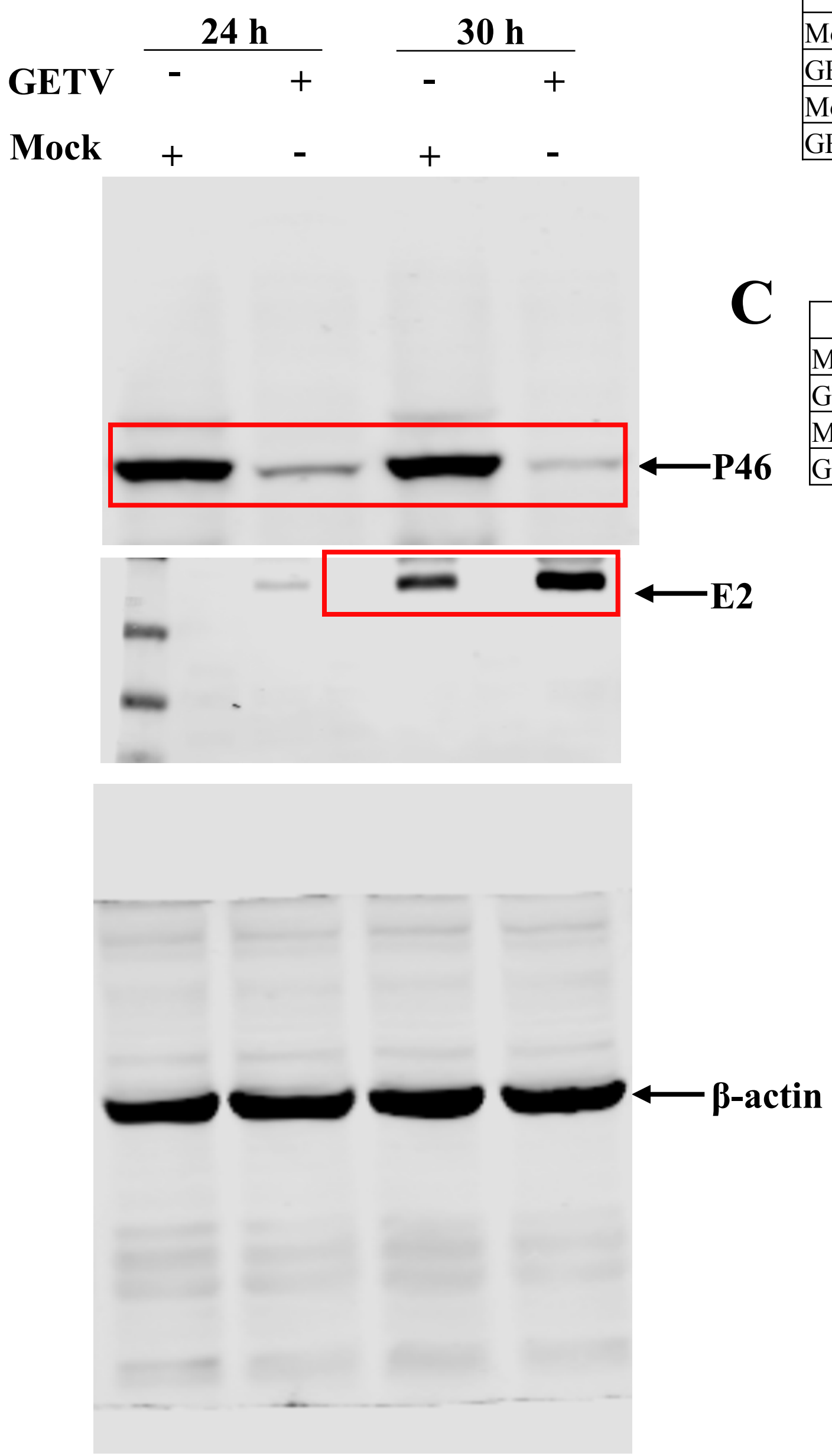

B

	repeat 1#	repeat 2#	repeat 3#	mean	SD
Mock-24h	1.58	1.2	1.1	1.2933333	0.2532456
GETV-24h	0.35	0.19	0.25	0.2633333	0.080829
Mock-30h	1.24	1.34	1.49	1.3566667	0.1258306
GETV-30h	0.14	0.23	0.1	0.1566667	0.0665833

C

	repeat 1#	repeat 2#	repeat 3#	mean	SD
Mock-24h	1	1	1	1	0
GETV-24h	0.999264	1.006956	1.064734	1.0236513	0.0357859
Mock-30h	1	1	1	1	0
GETV-30h	0.980078	0.958791	0.998791	0.97922	0.0200138

D

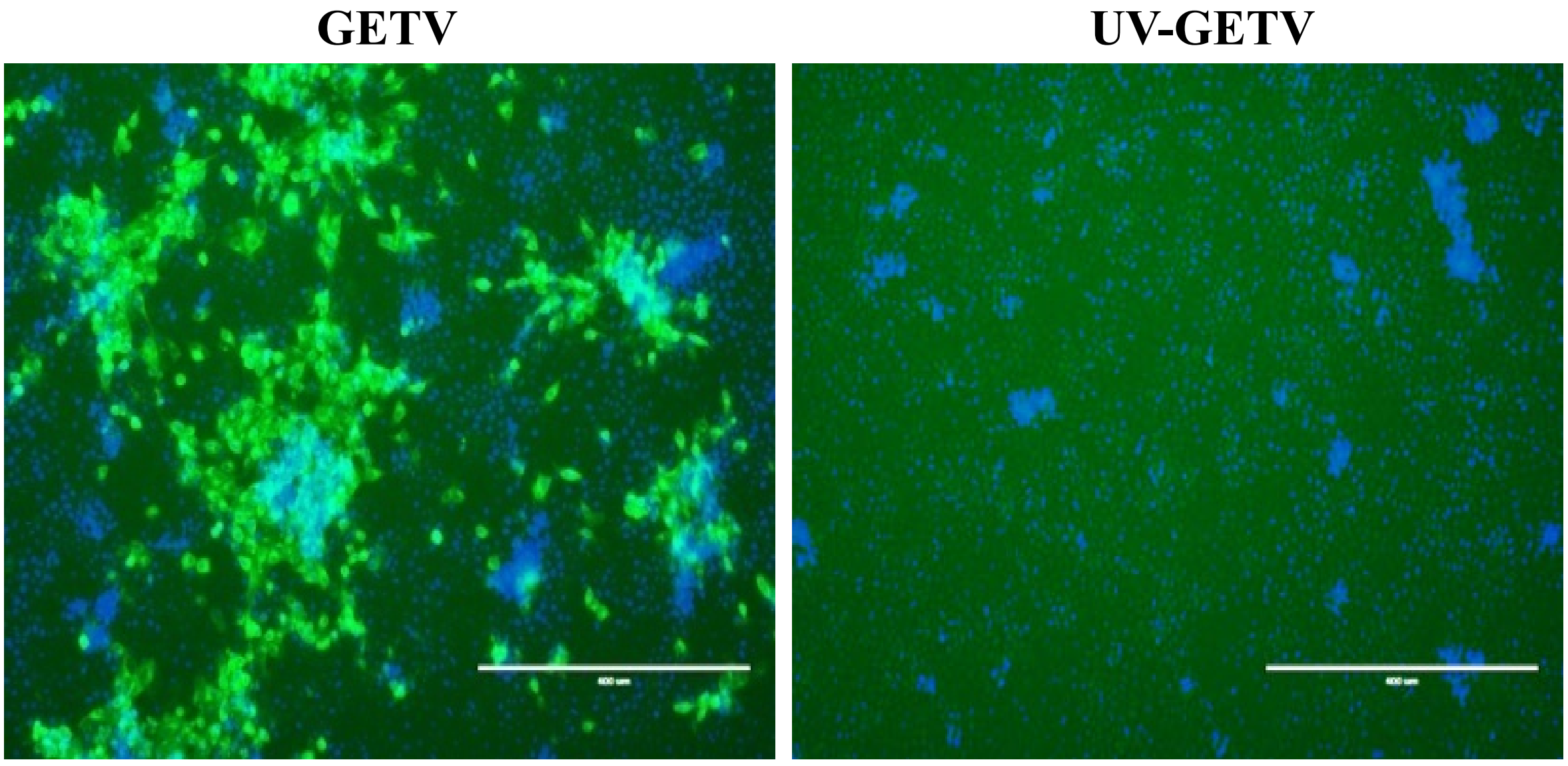

E

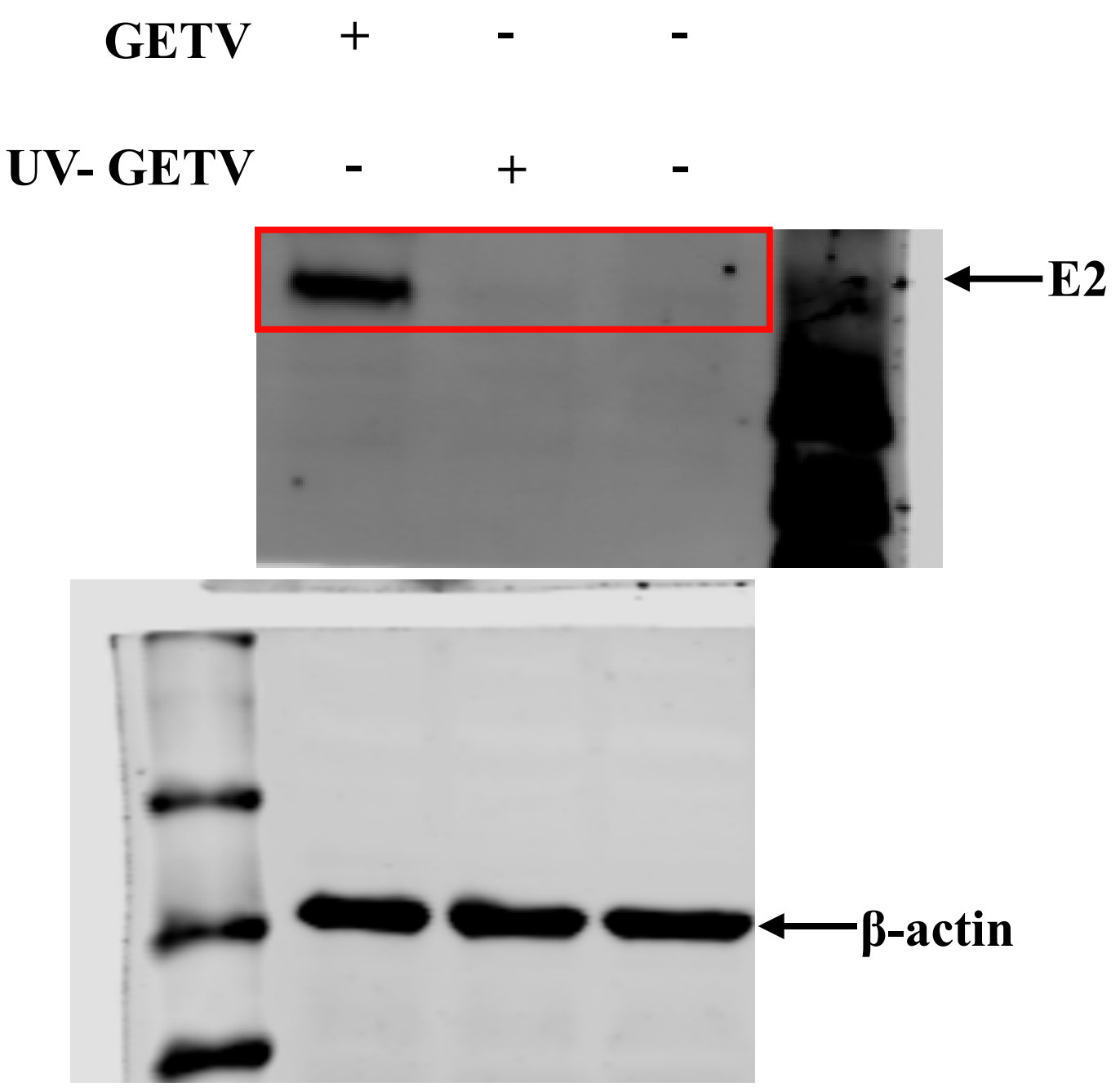

F

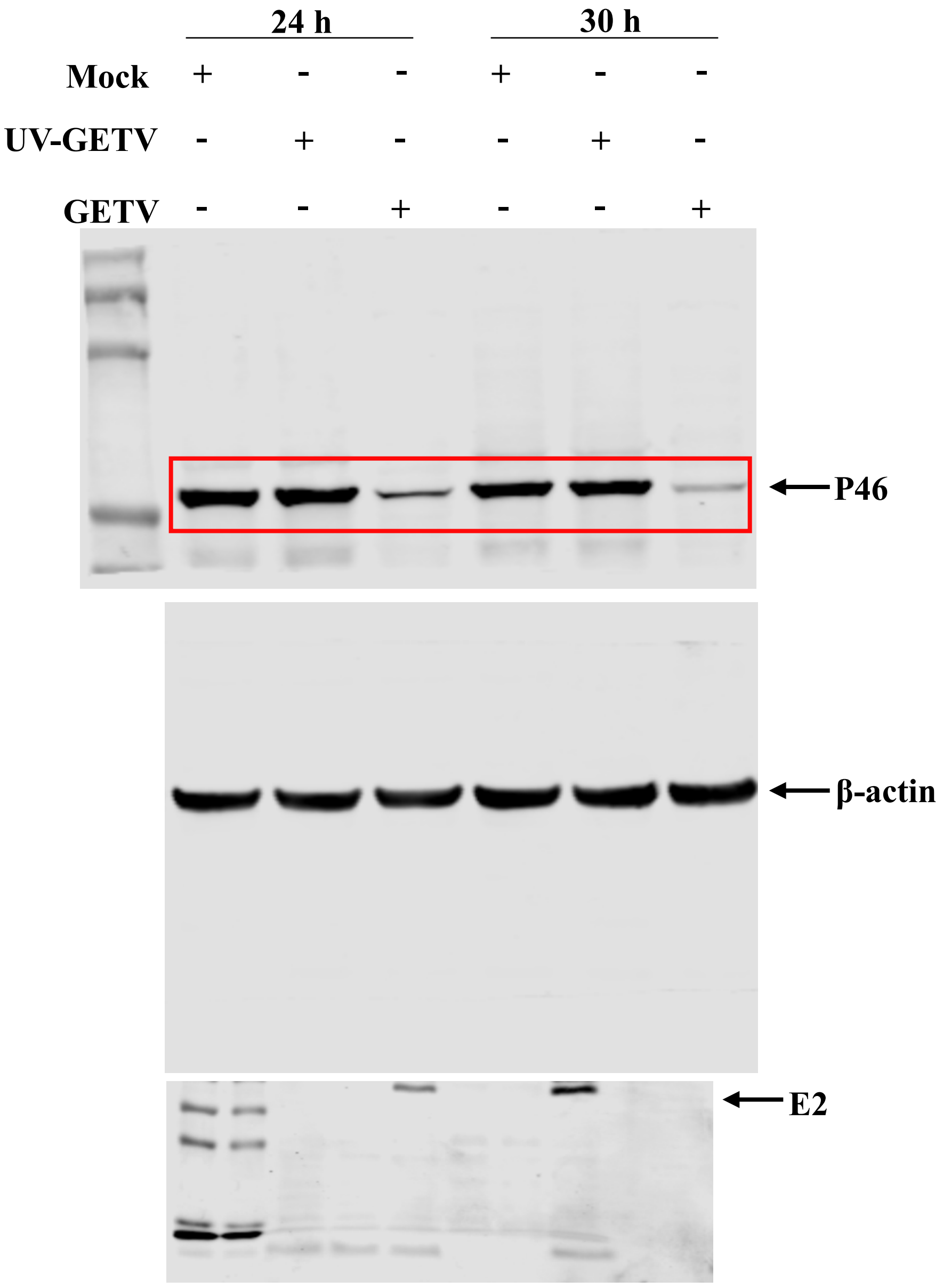

G

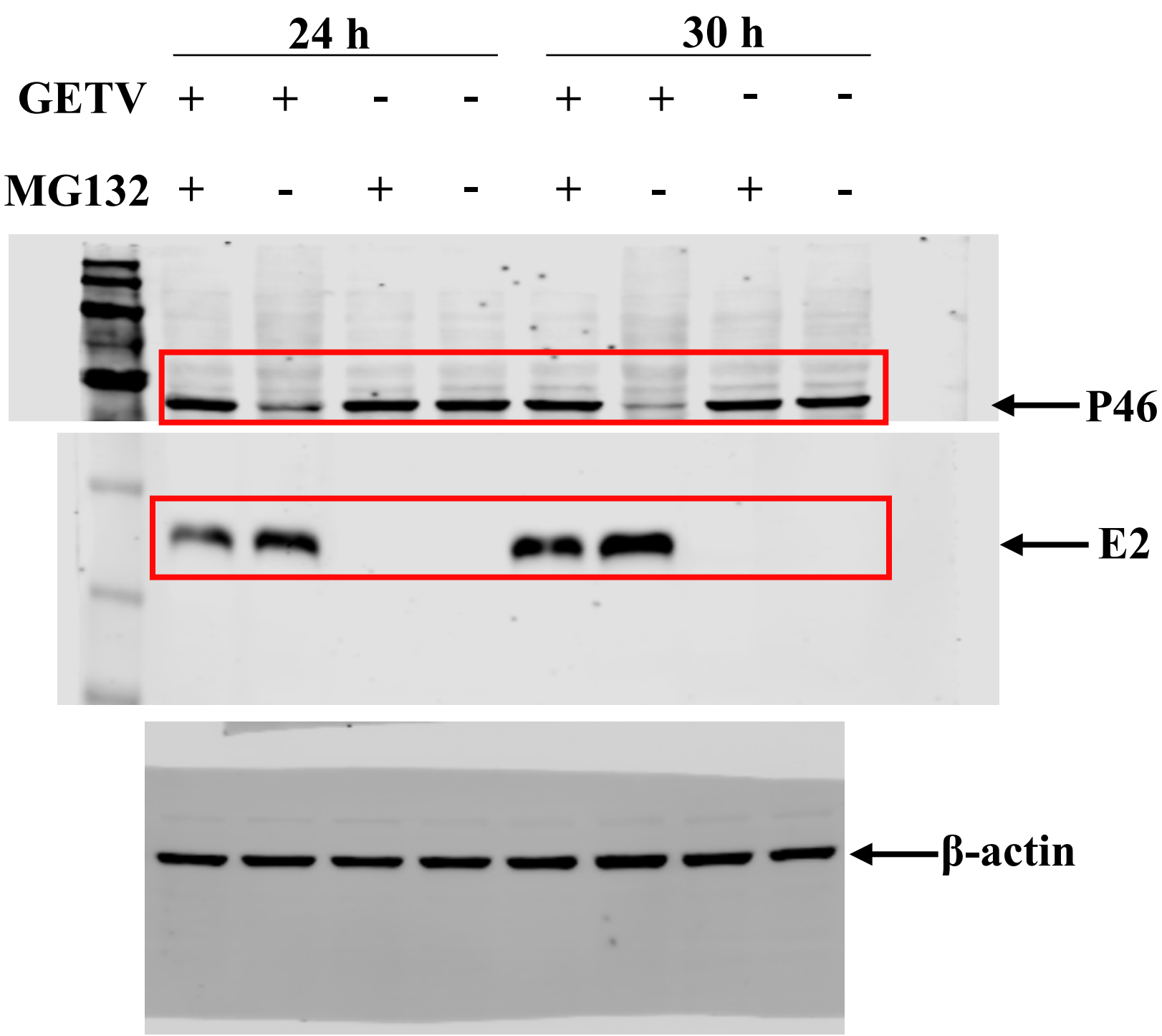
24 h
30 h
GETV + + - - + + - -
MG132 + - + - + - + -
P46
E2
β-actin

H

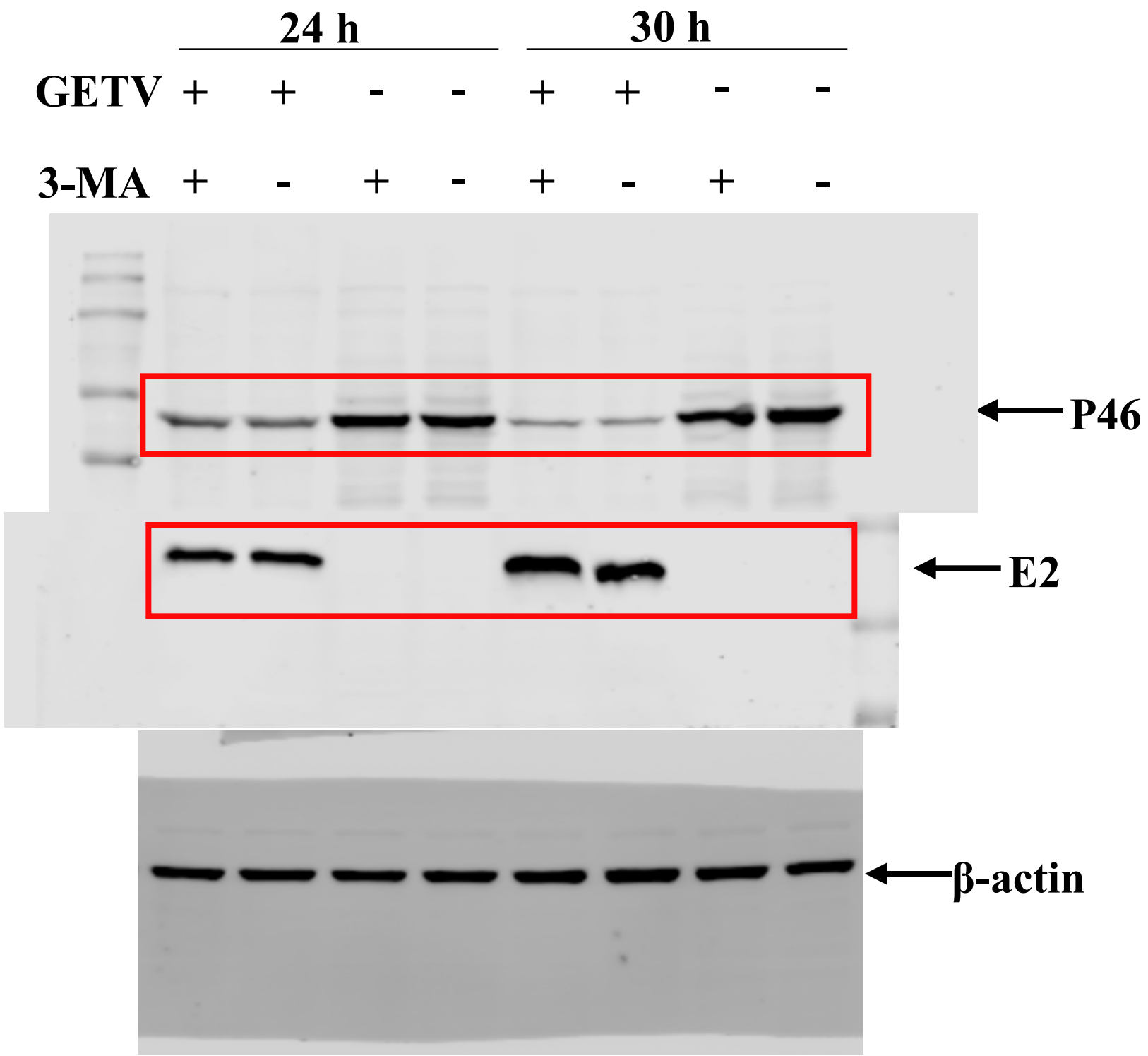
24 h
30 h
GETV + + - - + + - -
3-MA + - + - + - + -
P46
E2
β-actin

Fig. 6

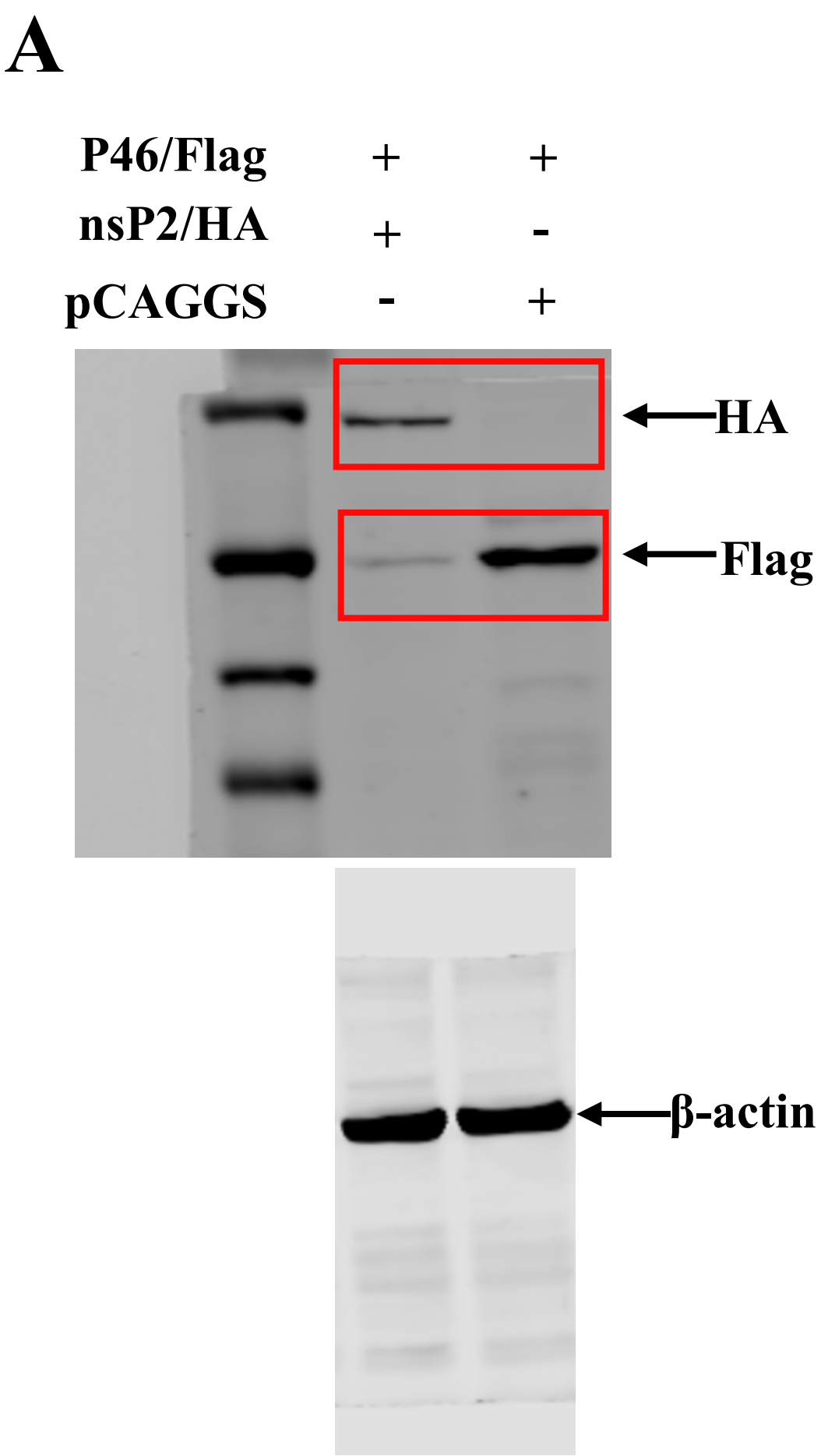
A
P46/Flag + +
nsP2/HA + -
pCAGGS - +
HA
Flag
β-actin

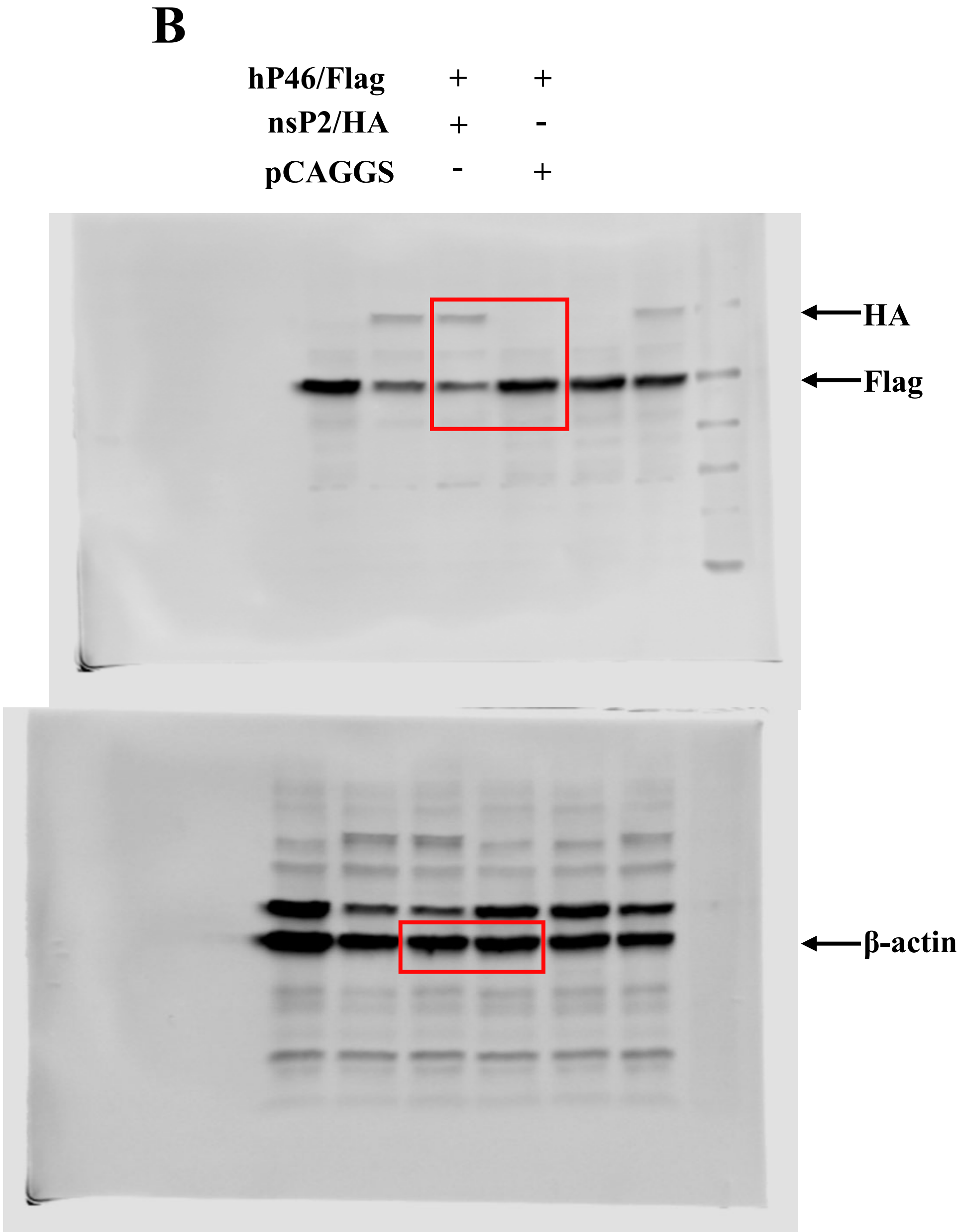
B
hP46/Flag + +
nsP2/HA + -
pCAGGS - +
HA
Flag
β-actin

C

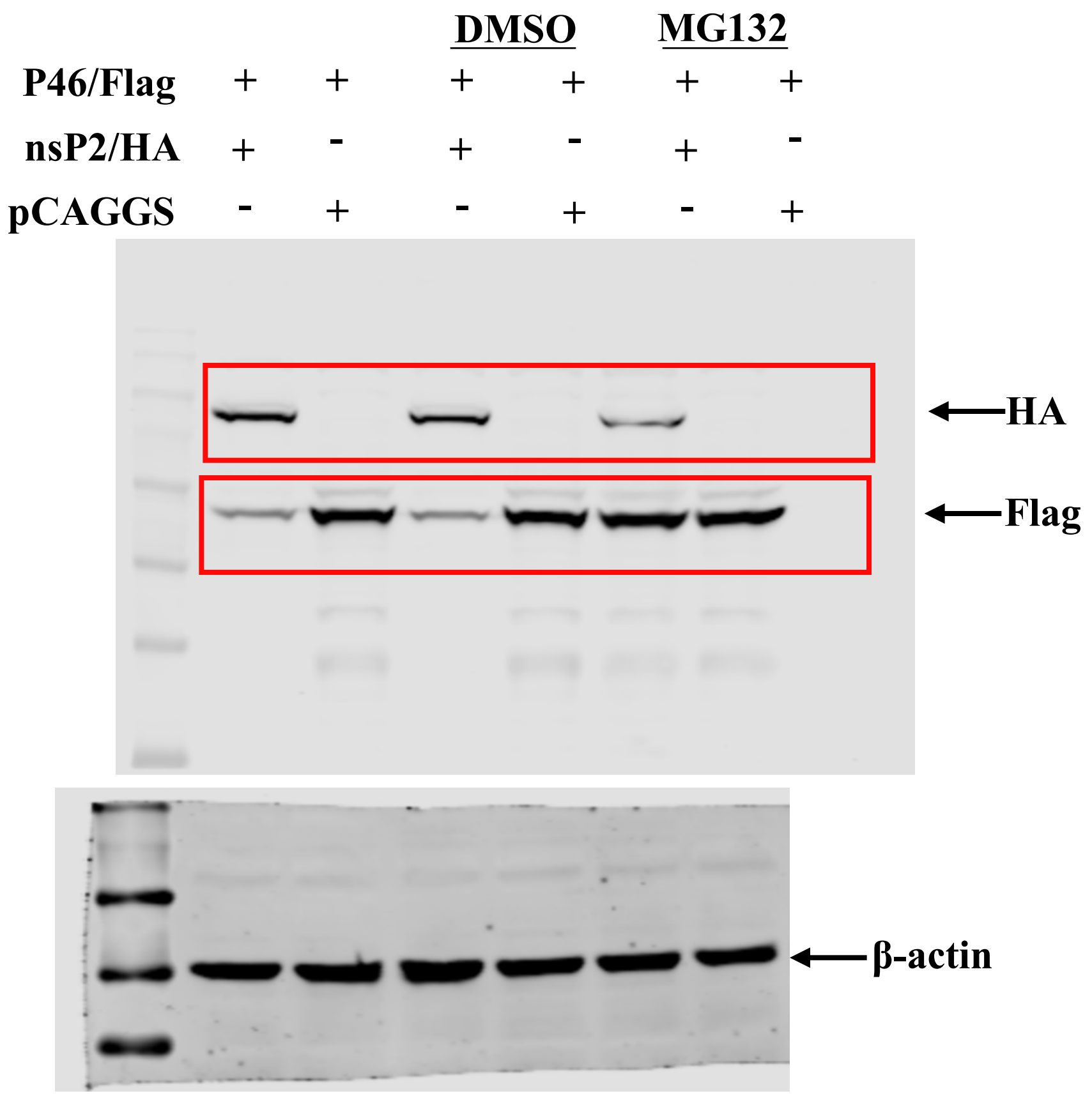
DMSO
MG132
P46/Flag + + + + + +
nsP2/HA + - + - + -
pCAGGS - + - + - +
HA
Flag
β-actin

D

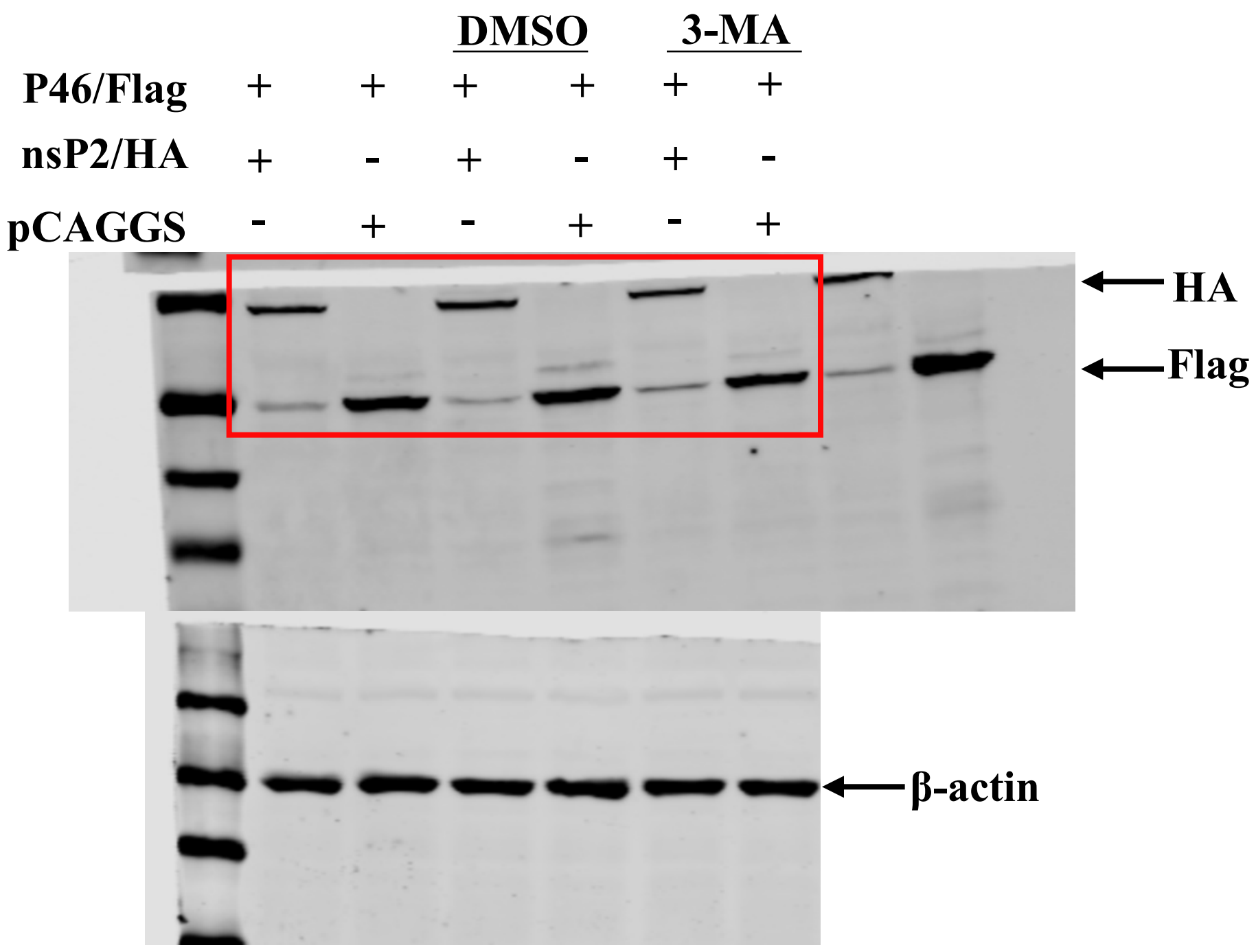
DMSO
3-MA
P46/Flag + + + + + +
nsP2/HA + - + - + -
pCAGGS - + - + - +
HA
Flag
β-actin

E F

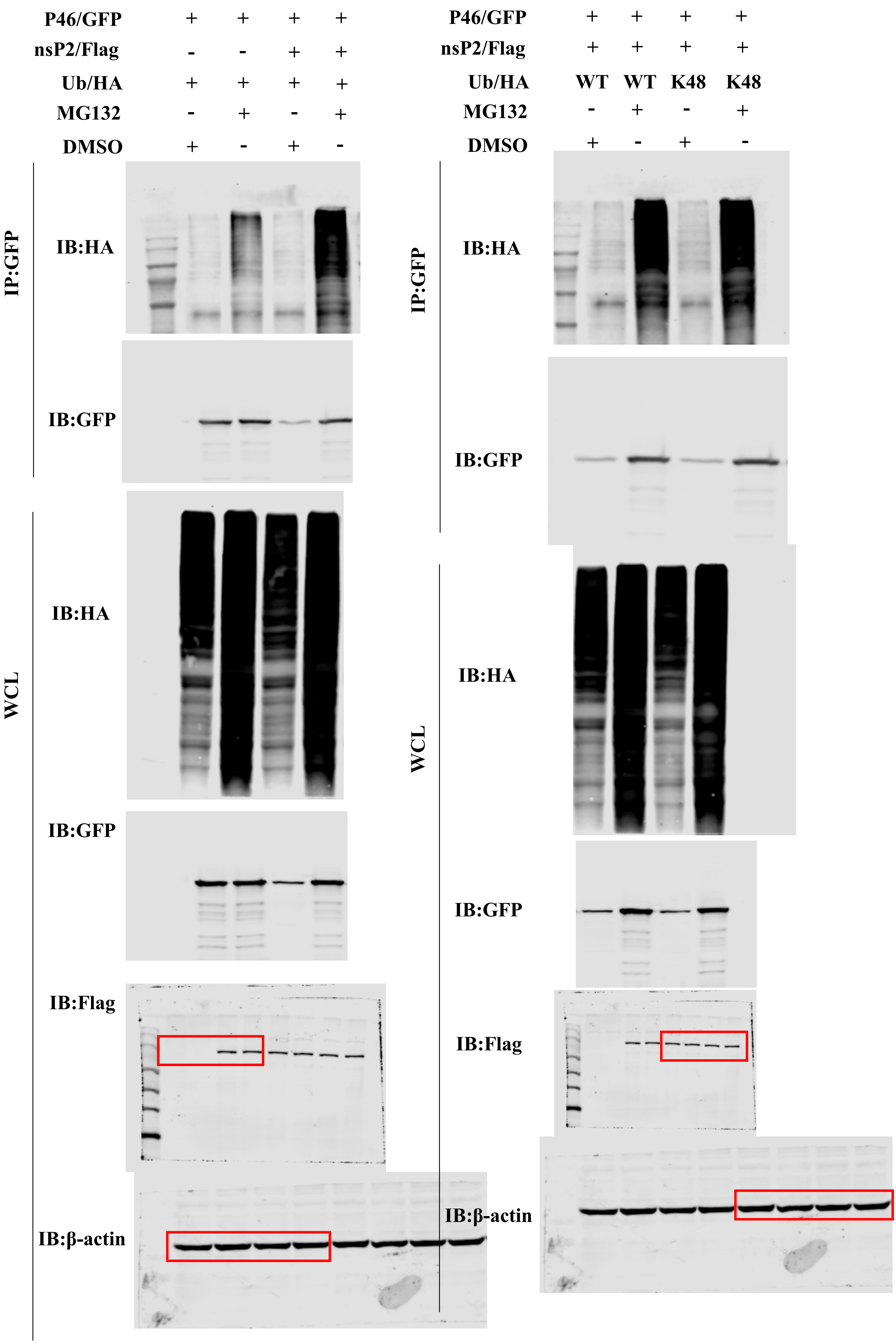

P46/GFP + + + +
nsP2/Flag - - + +
Ub/HA + + + +
MG132 - + - +
DMSO + - + -
IP:GFP
IB:HA
IB:GFP
WCL
IB:HA
IB:GFP
IB:Flag
IB:β-actin
P46/GFP + + + +
nsP2/Flag + + + +
Ub/HA WT WT K48 K48
MG132 - + - +
DMSO + - + -
IP:GFP
IB:HA
IB:GFP
WCL
IB:HA
IB:GFP
IB:Flag
IB:β-actin

Fig. 7

A

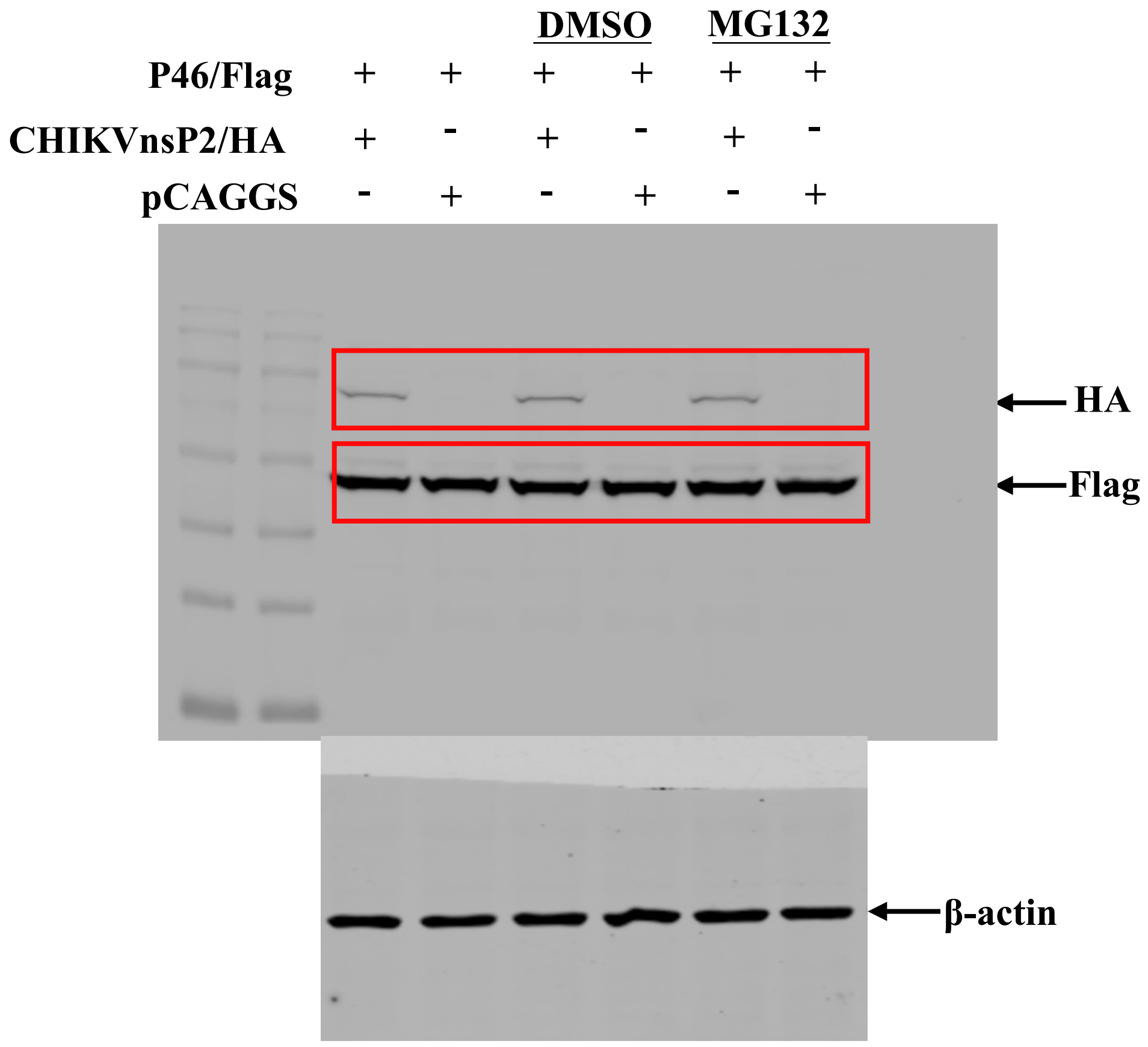

DMSO
MG132
P46/Flag + + + + + +
CHIKVnsP2/HA + - + - + -
pCAGGS - + - + - +
HA
Flag
β-actin

B

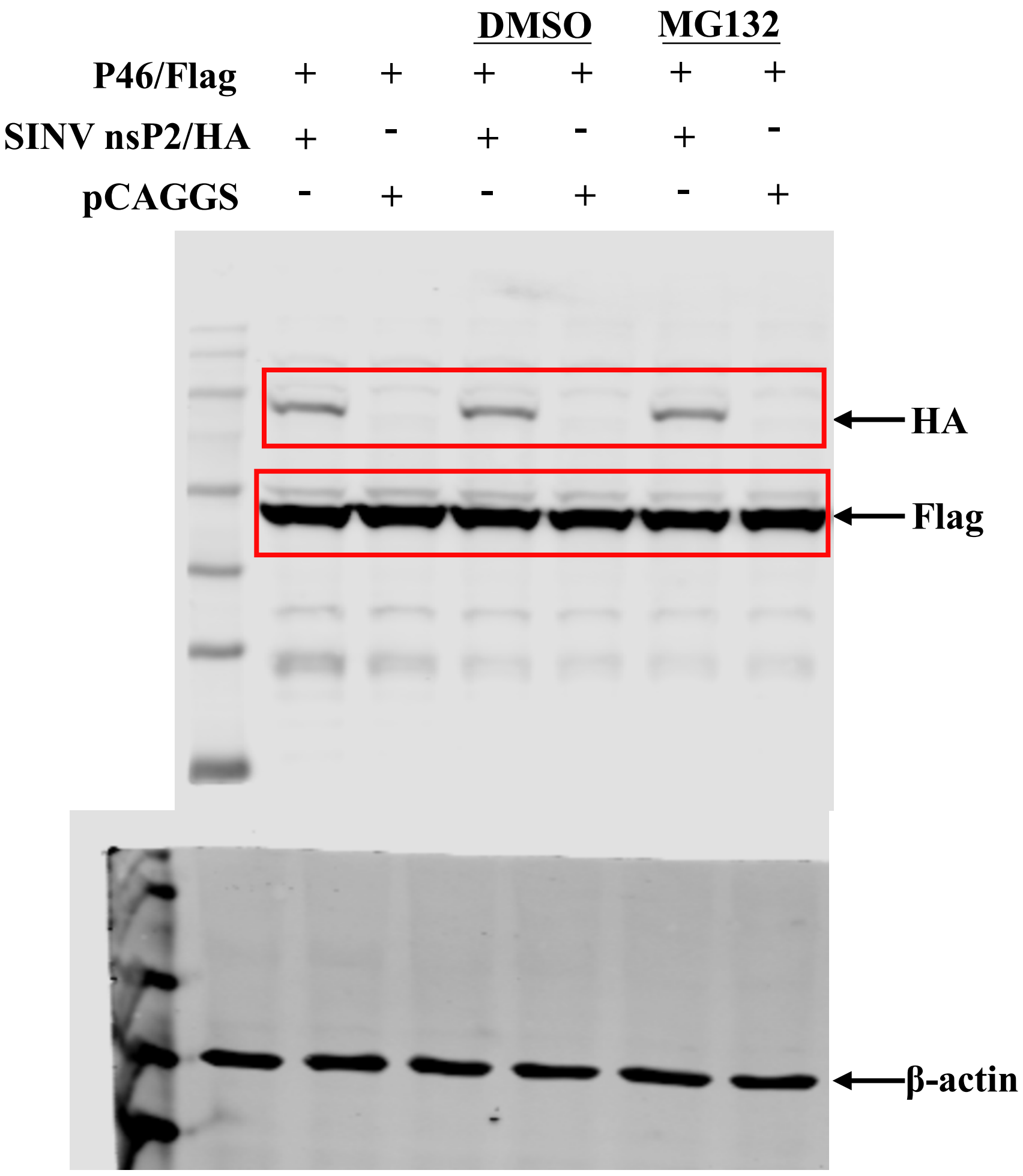

DMSO
MG132
P46/Flag + + + + + +
SINV nsP2/HA + - + - + -
pCAGGS - + - + - +
HA
Flag
β-actin

C

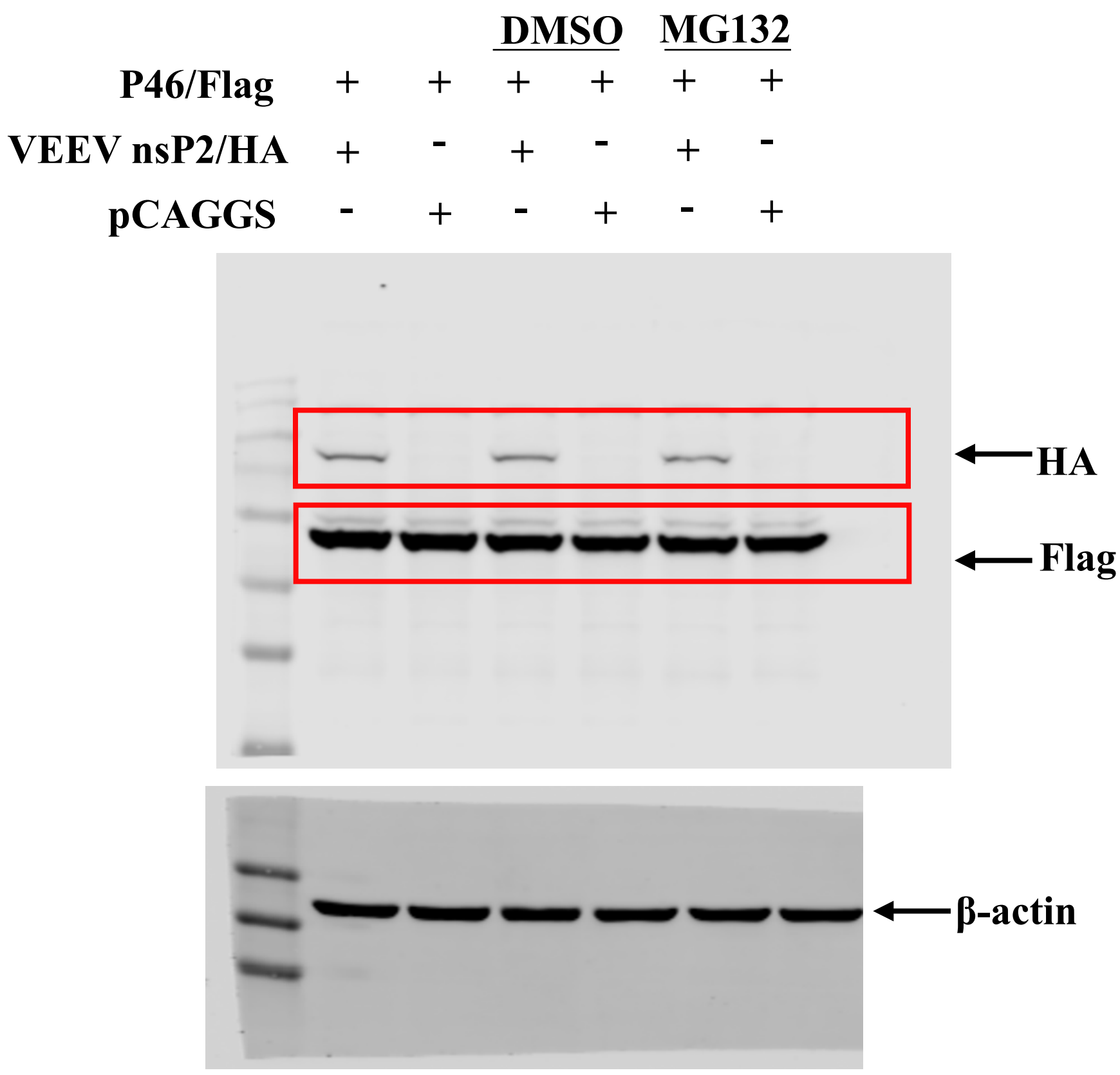
DMSO
MG132
P46/Flag + + + + + +
VEEV nsP2/HA + - + - + -
pCAGGS - + - + - +
HA
Flag
β-actin

D

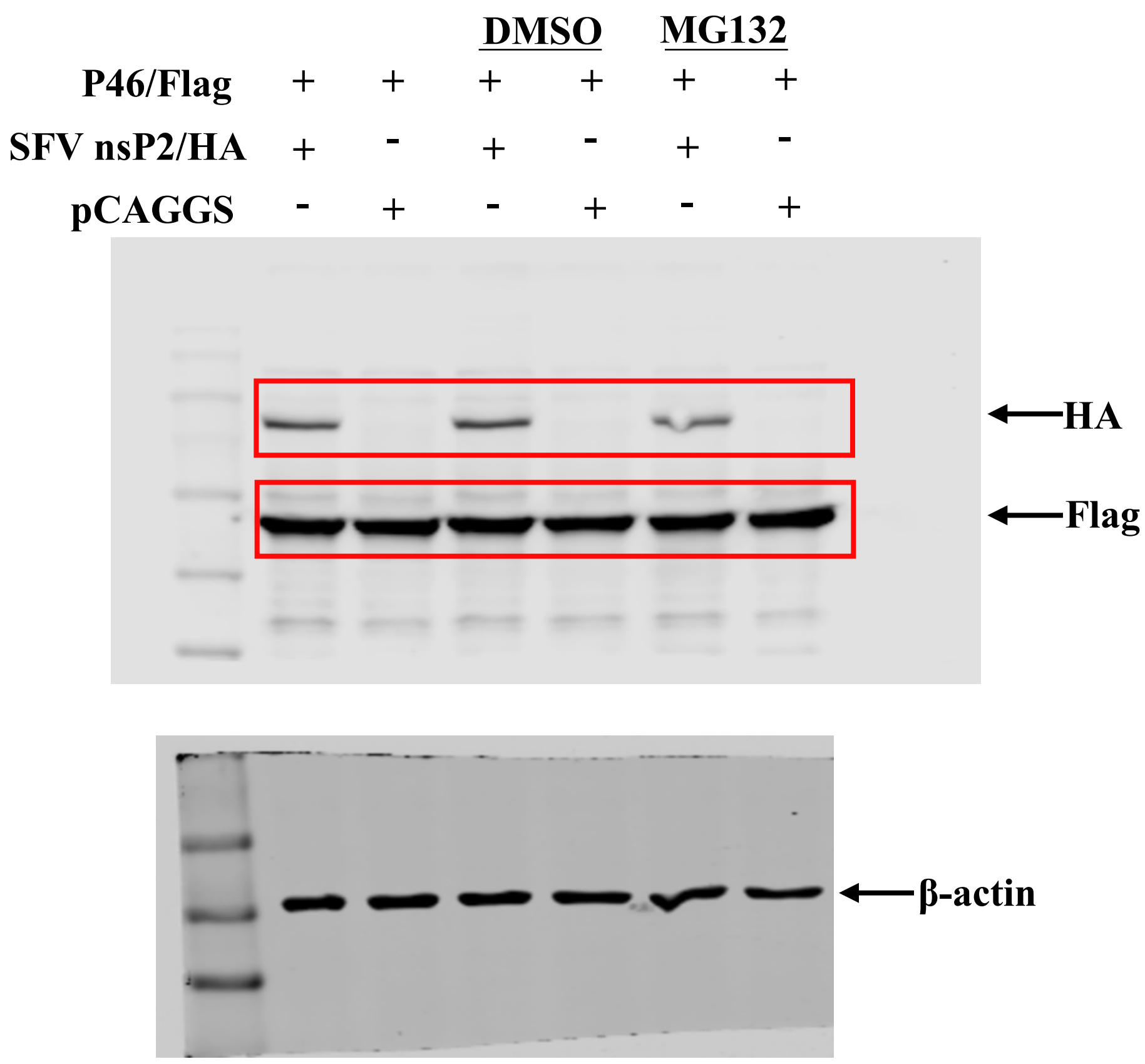
DMSO
MG132
P46/Flag + + + + + +
SFV nsP2/HA + - + - + -
pCAGGS - + - + - +
HA
Flag
β-actin

E

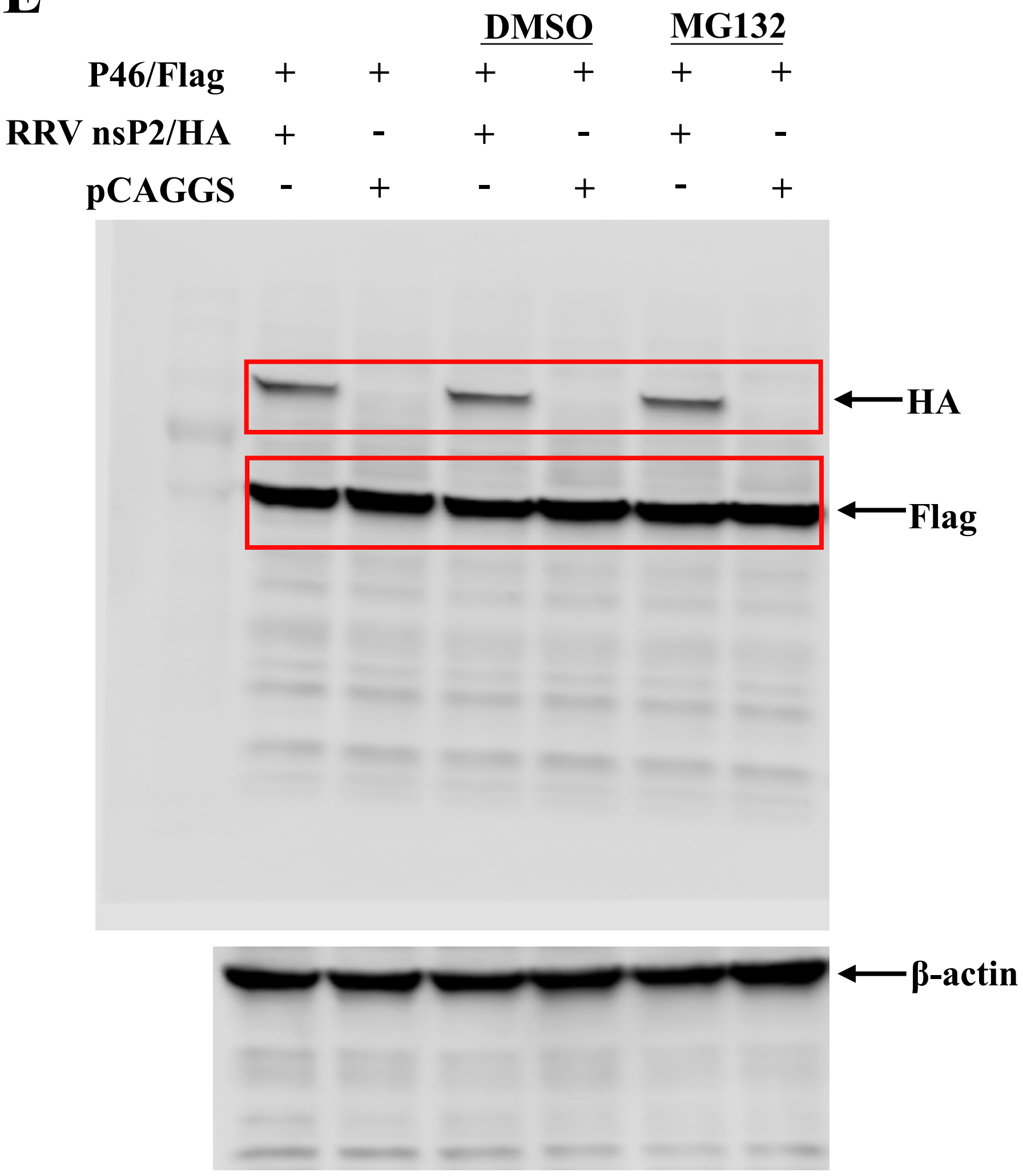
DMSO
MG132
P46/Flag + + + + + +
RRV nsP2/HA + - + - + -
pCAGGS - + - + - +
HA
Flag
β-actin

F

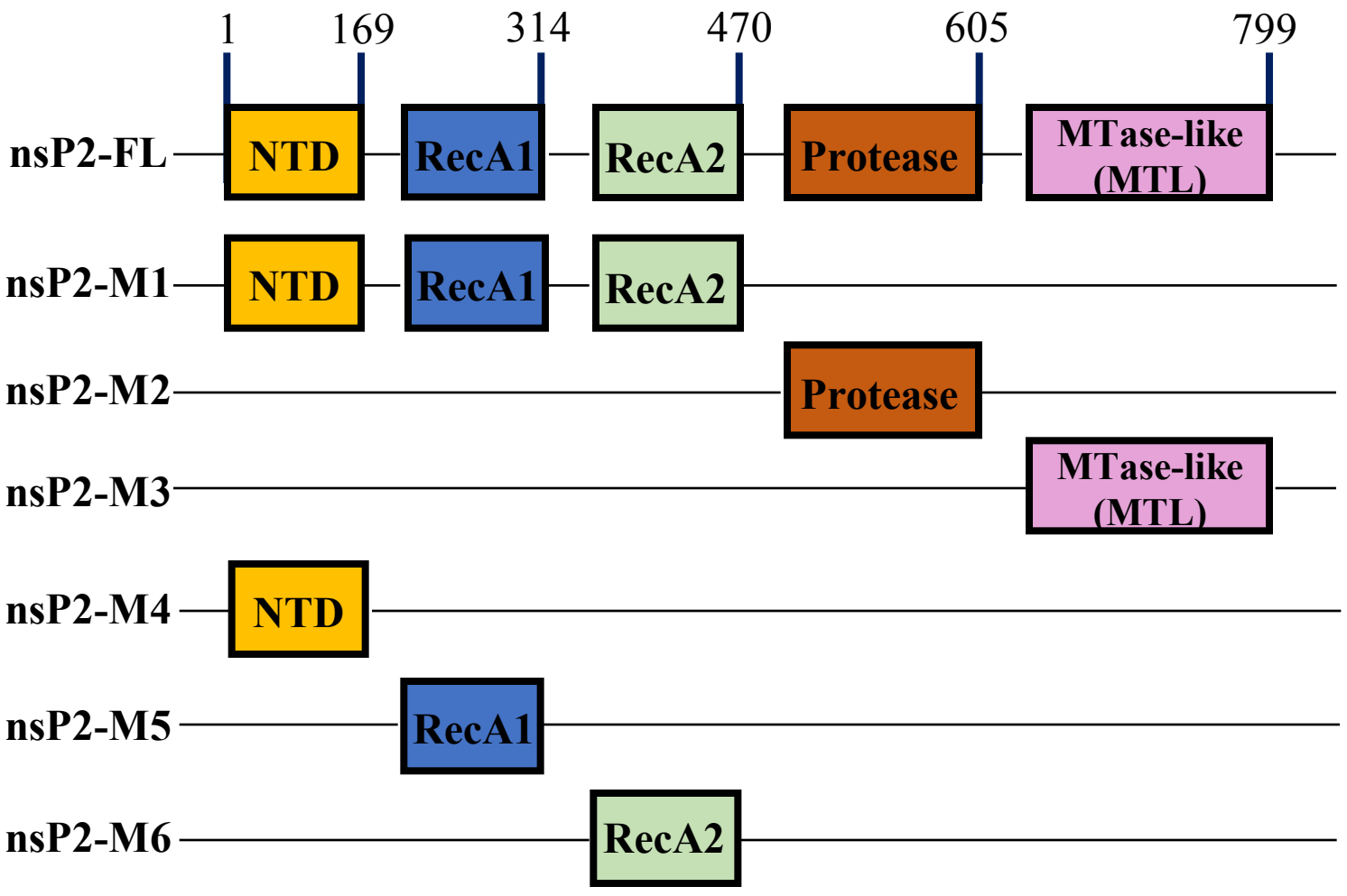
1
169
314
470
605
799
nsP2-FL
NTD
RecA1
RecA2
Protease
MTase-like (MTL)
nsP2-M1
nsP2-M2
nsP2-M3
nsP2-M4
nsP2-M5
nsP2-M6

G

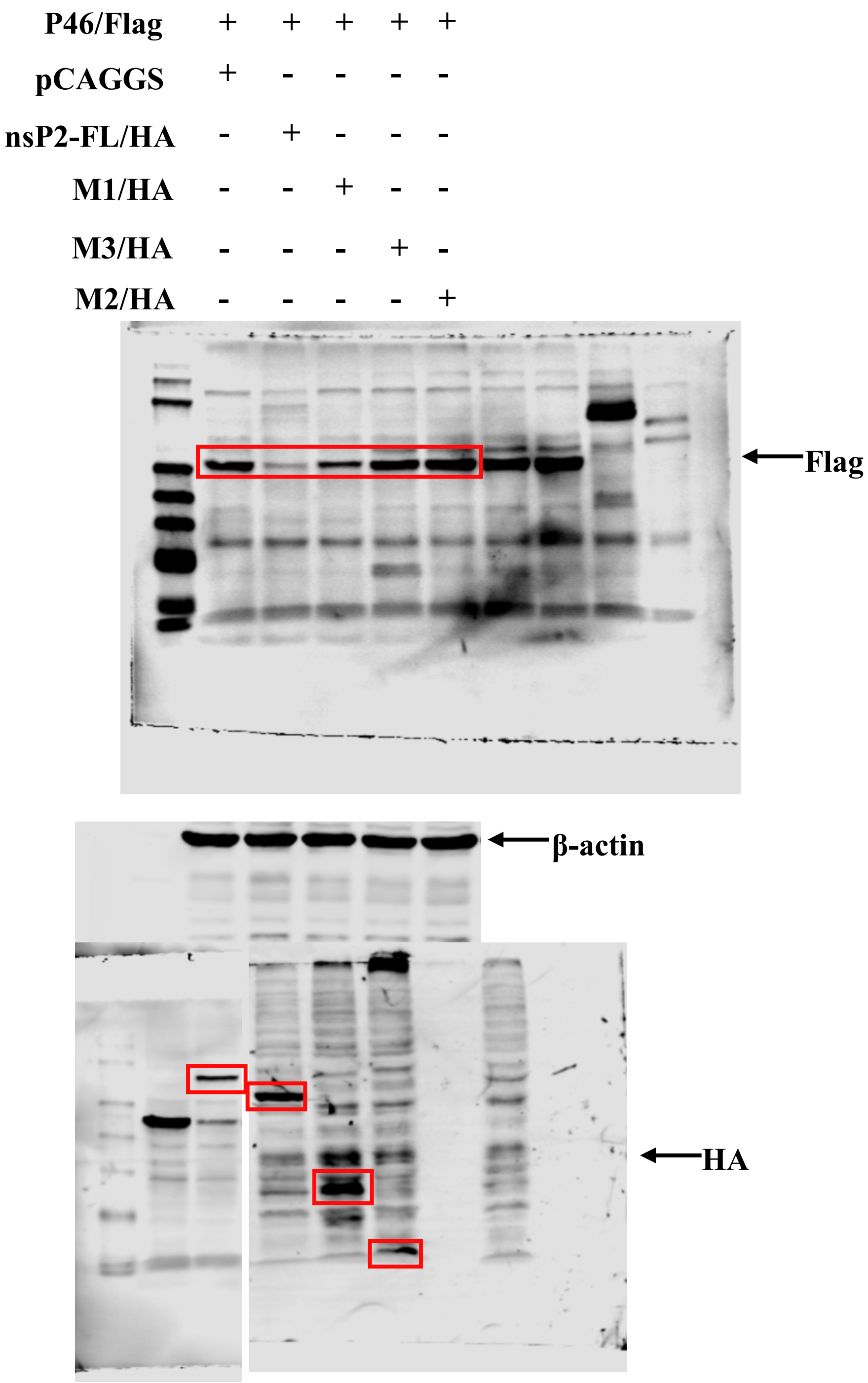

H

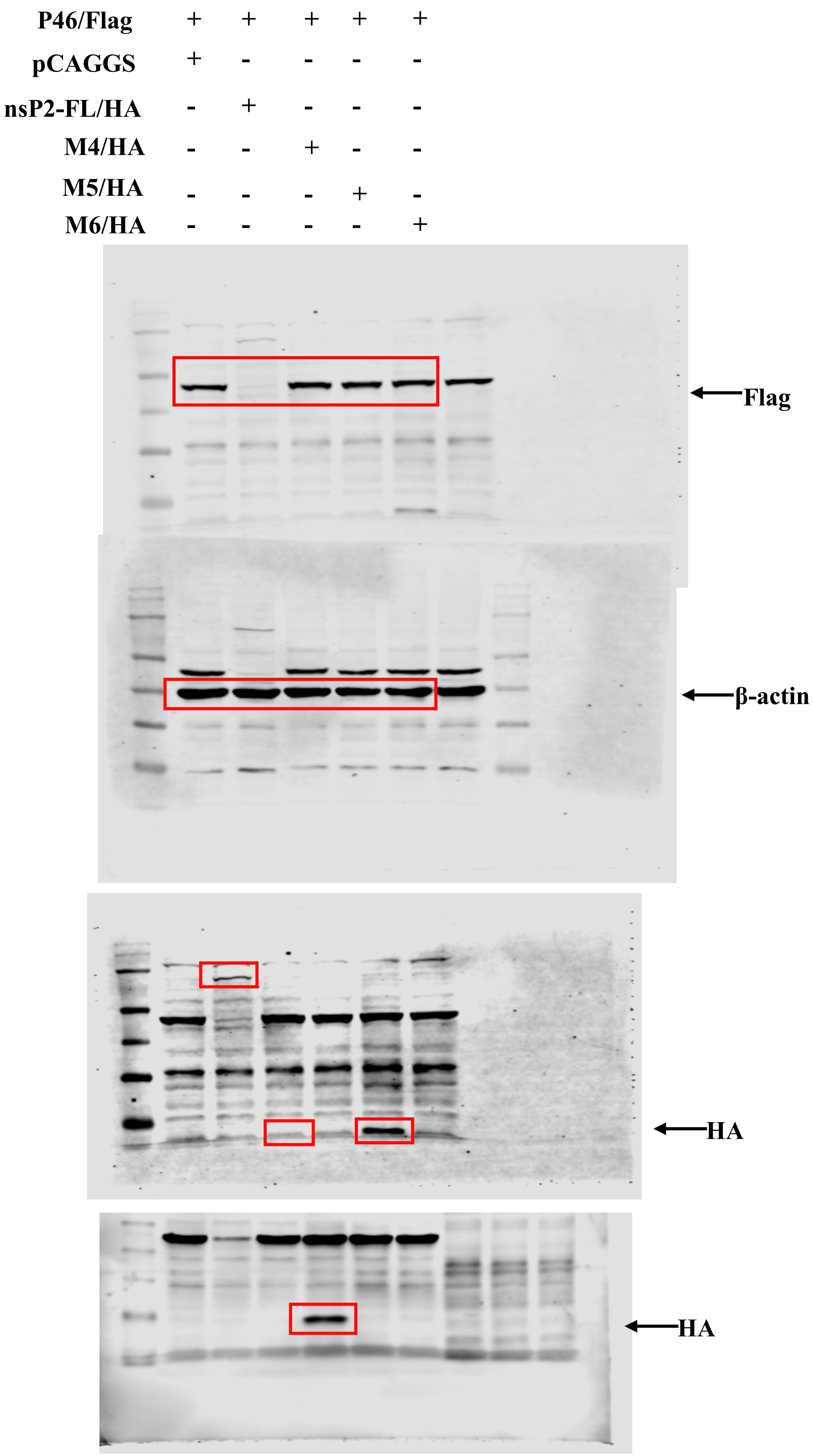

S3 Fig

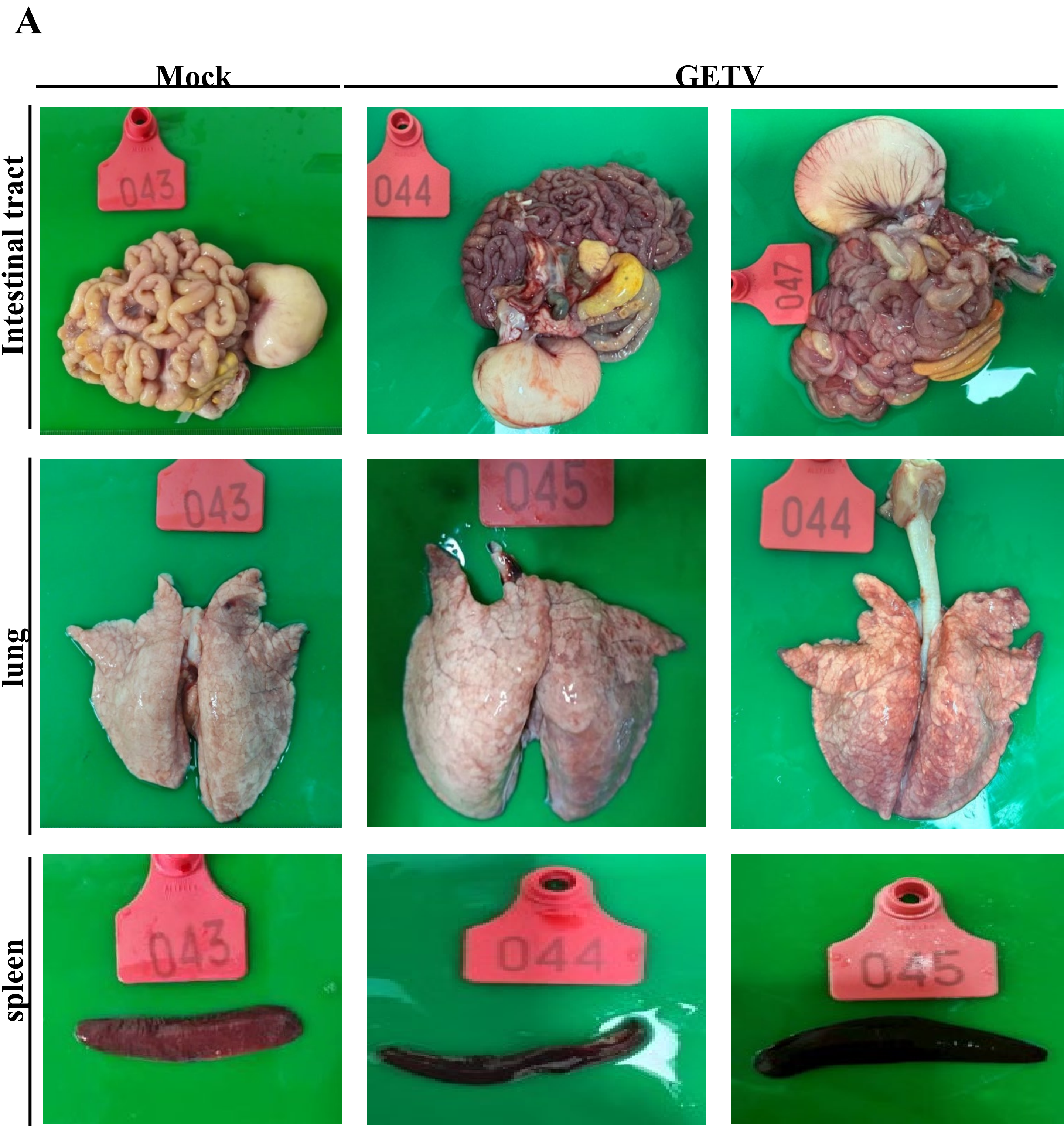
A
Mock
GETV
Intestinal tract
lung
spleen
043
044
047
043
045
044
043
044
045

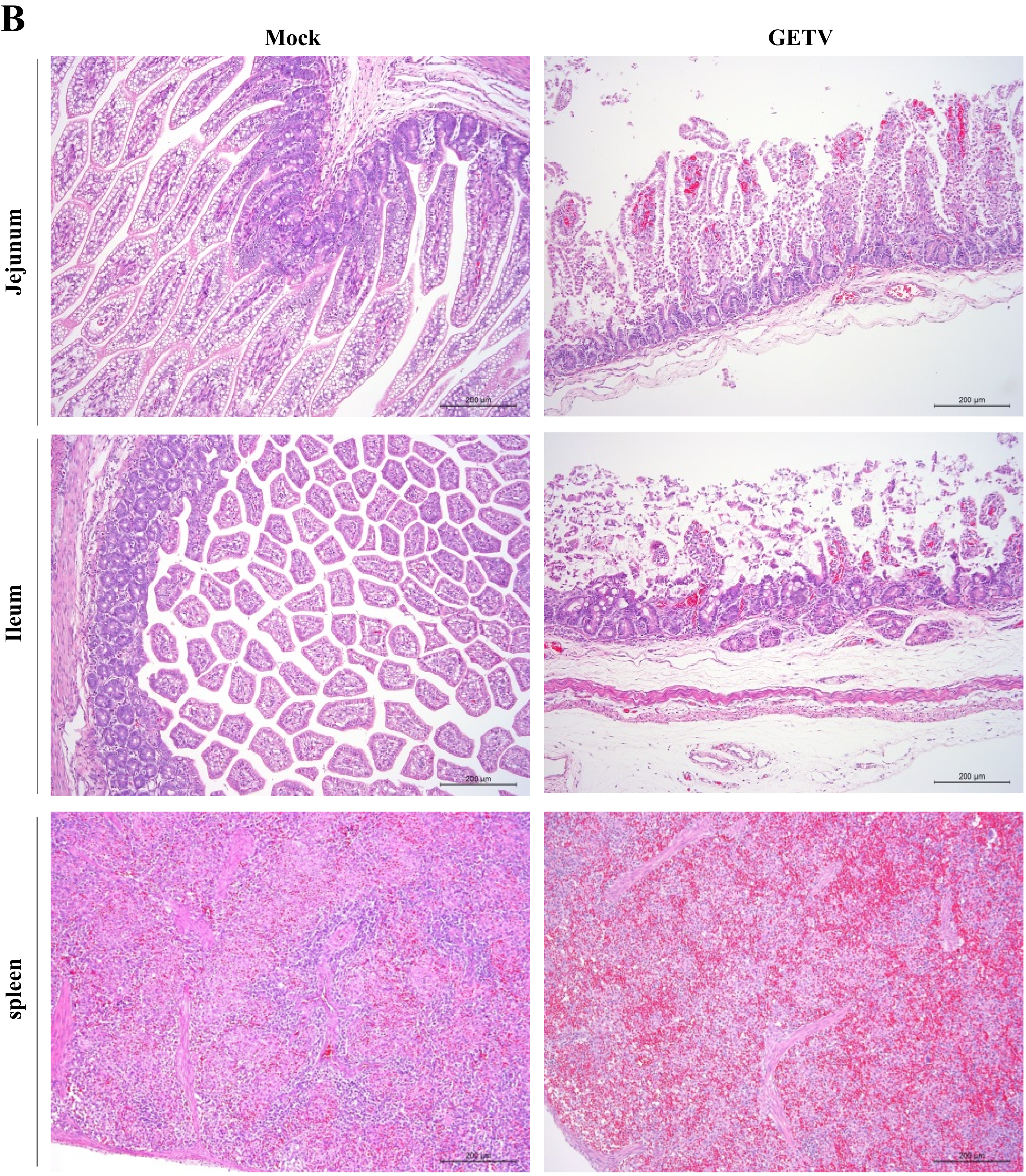
B
Mock
GETV
Jejunum
Ileum
spleen
200 μm

S4 Fig

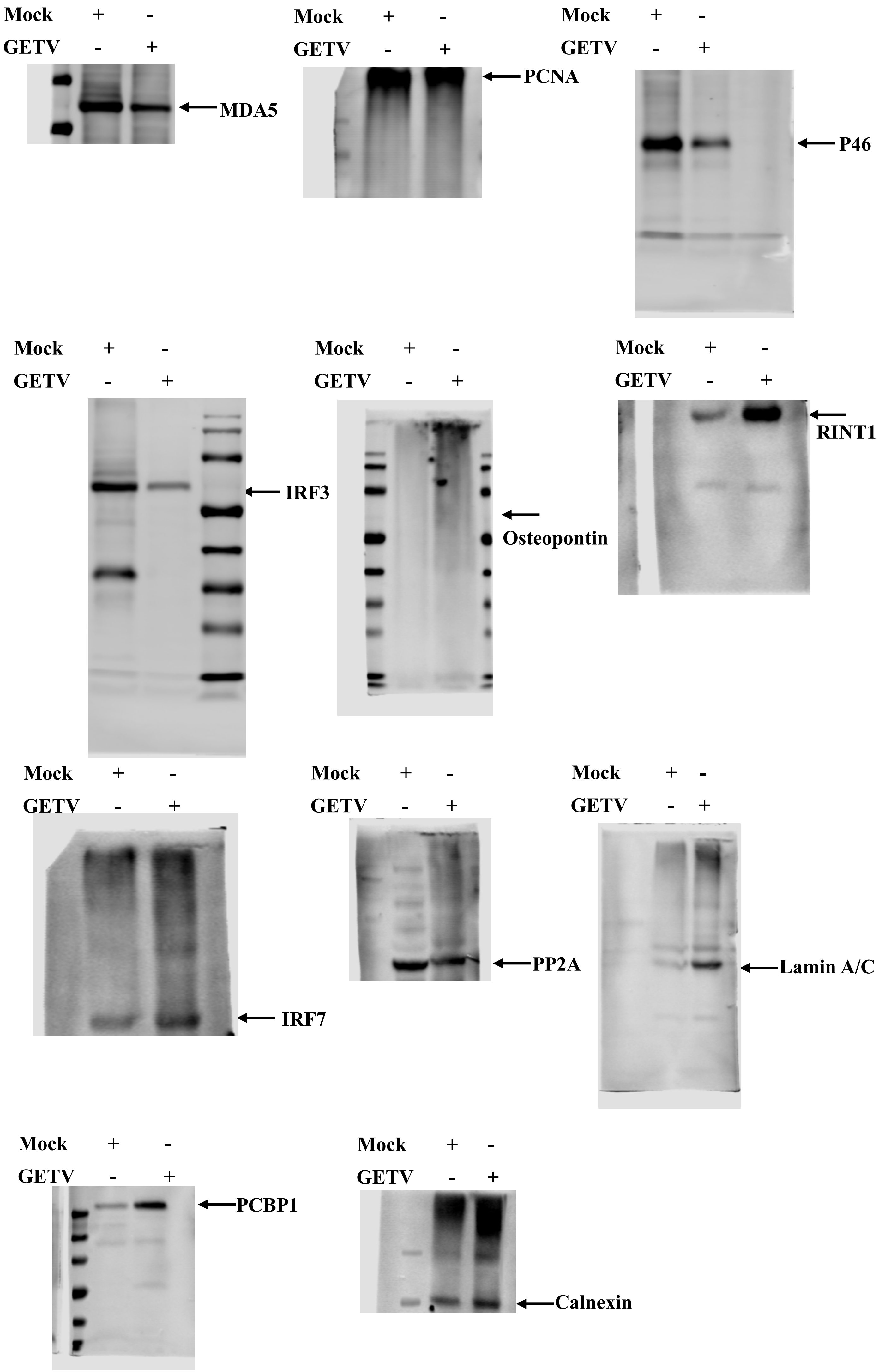
Mock + -
GETV - +
MDA5
PCNA
P46
IRF3
Osteopontin
RINT1
IRF7
PP2A
Lamin A/C
PCBP1
Calnexin

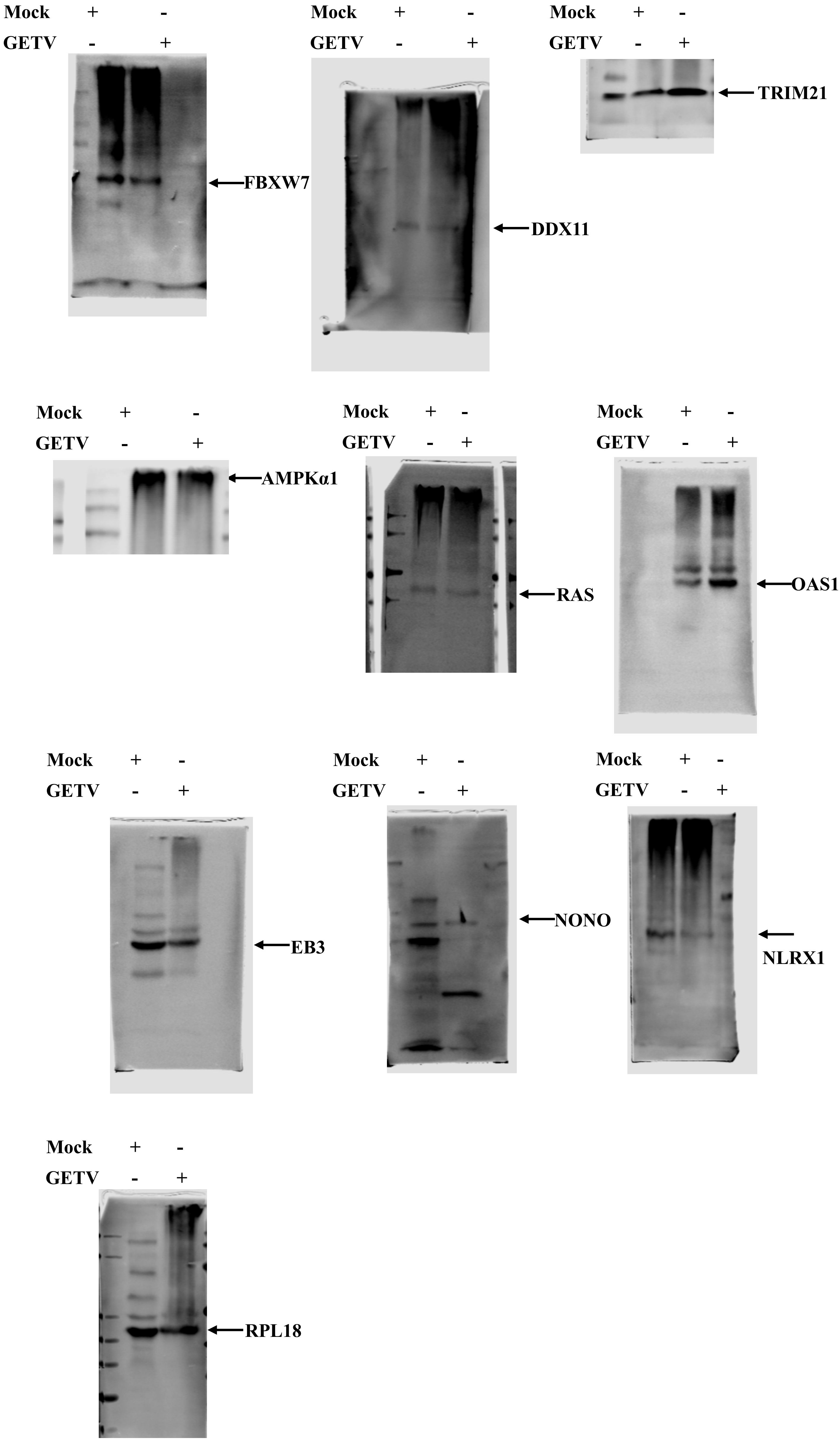
Mock + -
GETV - +
FBXW7
Mock + -
GETV - +
DDX11
Mock + -
GETV - +
TRIM21
Mock + -
GETV - +
AMPKα1
Mock + -
GETV - +
RAS
Mock + -
GETV - +
OAS1
Mock + -
GETV - +
EB3
Mock + -
GETV - +
NONO
Mock + -
GETV - +
NLRX1
Mock + -
GETV - +
RPL18

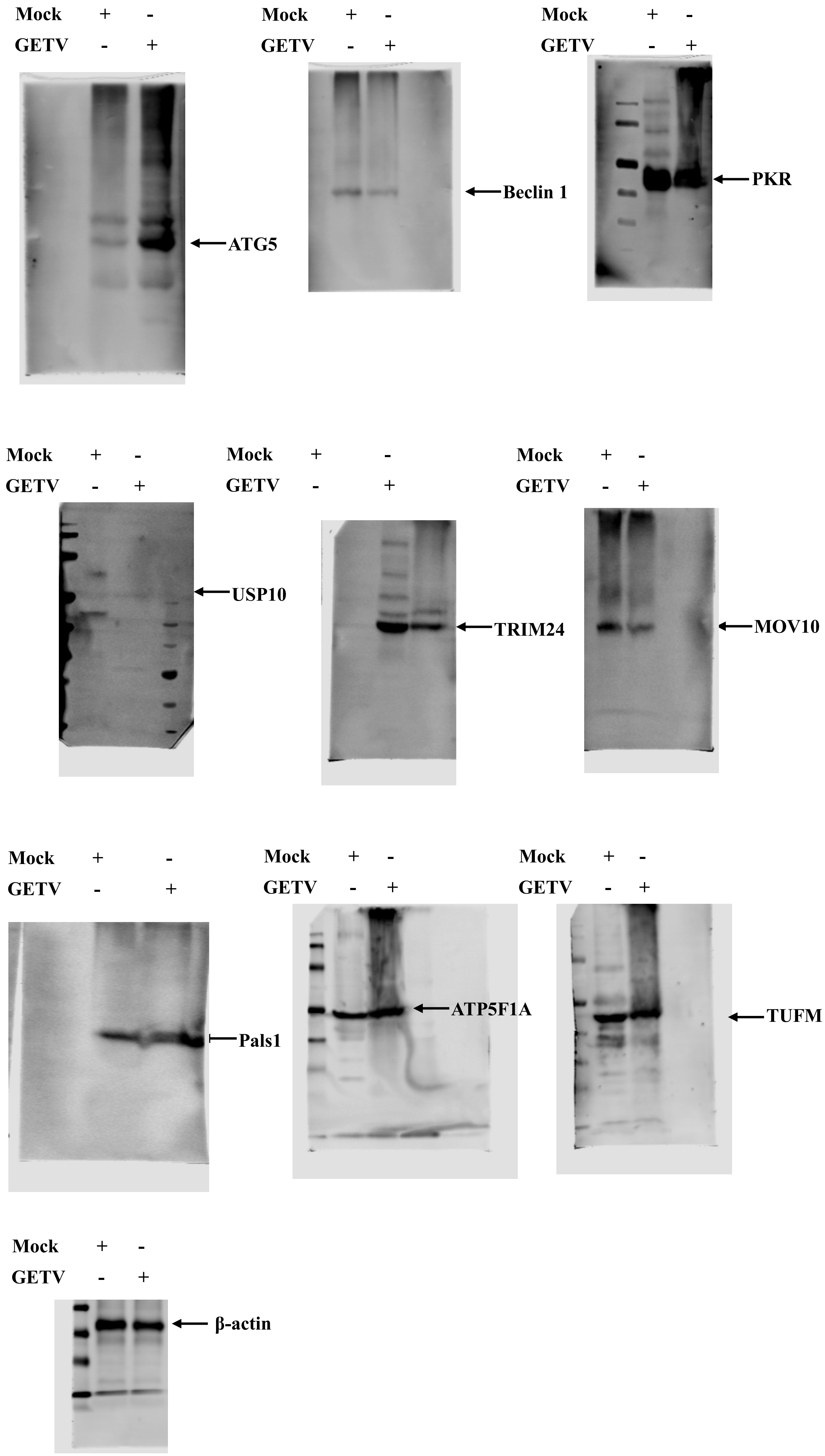

Mock + -
GETV - +
ATG5
Mock + -
GETV - +
Beclin 1
Mock + -
GETV - +
PKR
Mock + -
GETV - +
USP10
Mock + -
GETV - +
TRIM24
Mock + -
GETV - +
MOV10
Mock + -
GETV - +
Pals1
Mock + -
GETV - +
ATP5F1A
Mock + -
GETV - +
TUFM
Mock + -
GETV - +
β-actin

S5 Fig

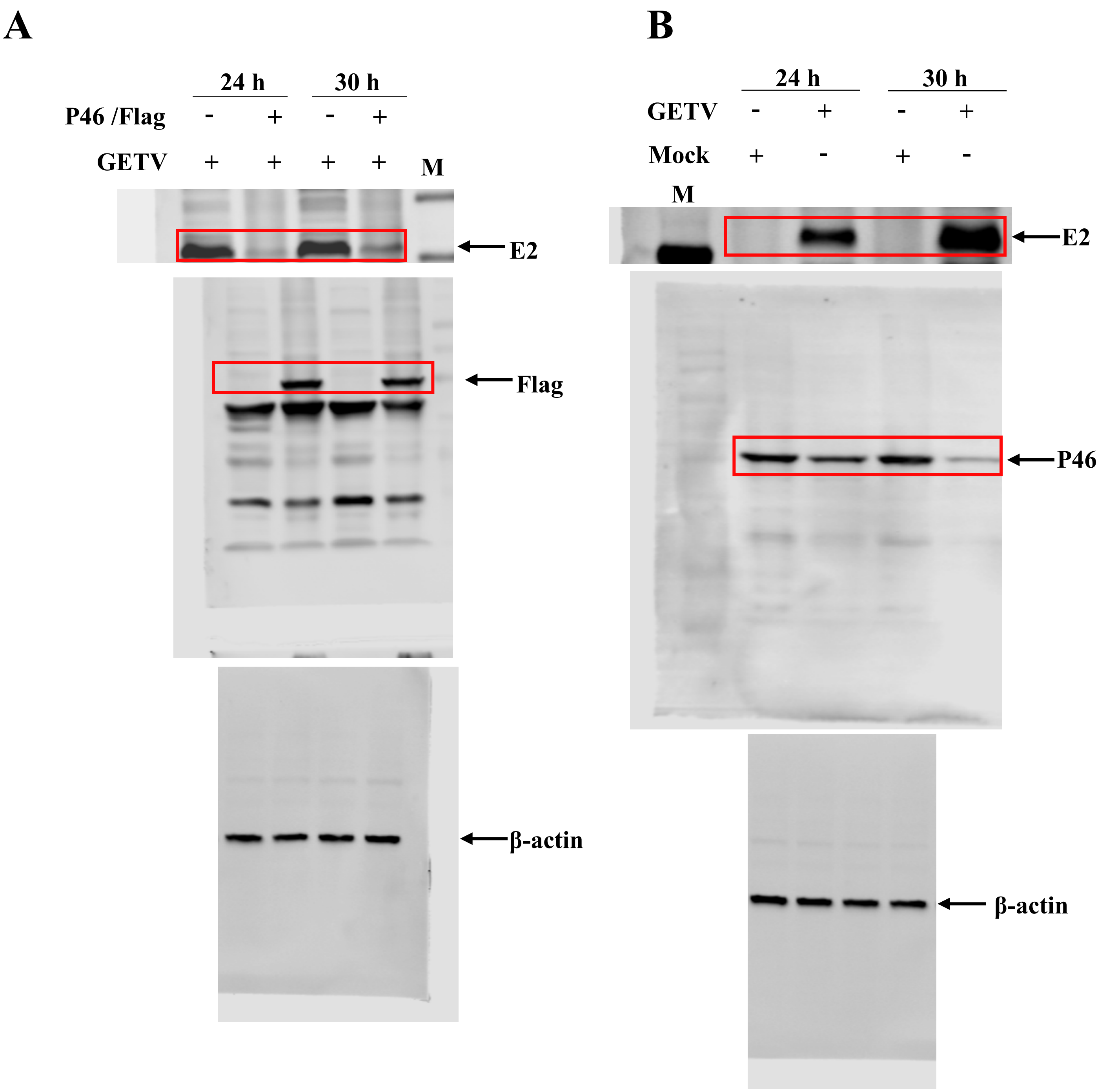
A
24 h
30 h
P46 /Flag
-
+
-
+
GETV
+
+
+
+
M
E2
Flag
β-actin
B
24 h
30 h
GETV
-
+
-
+
Mock
+
-
+
-
M
E2
P46
β-actin

C

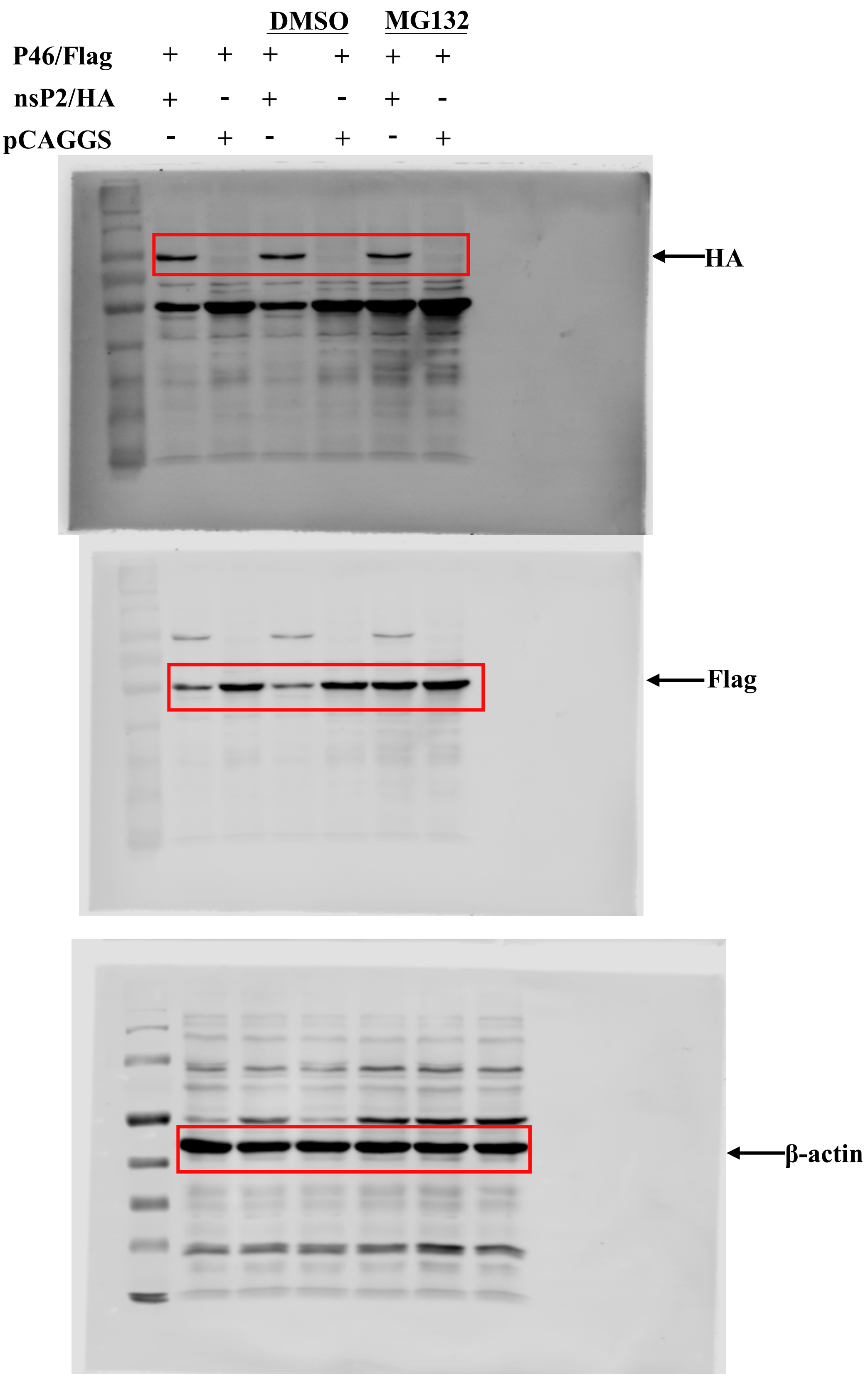

S7 Fig

A

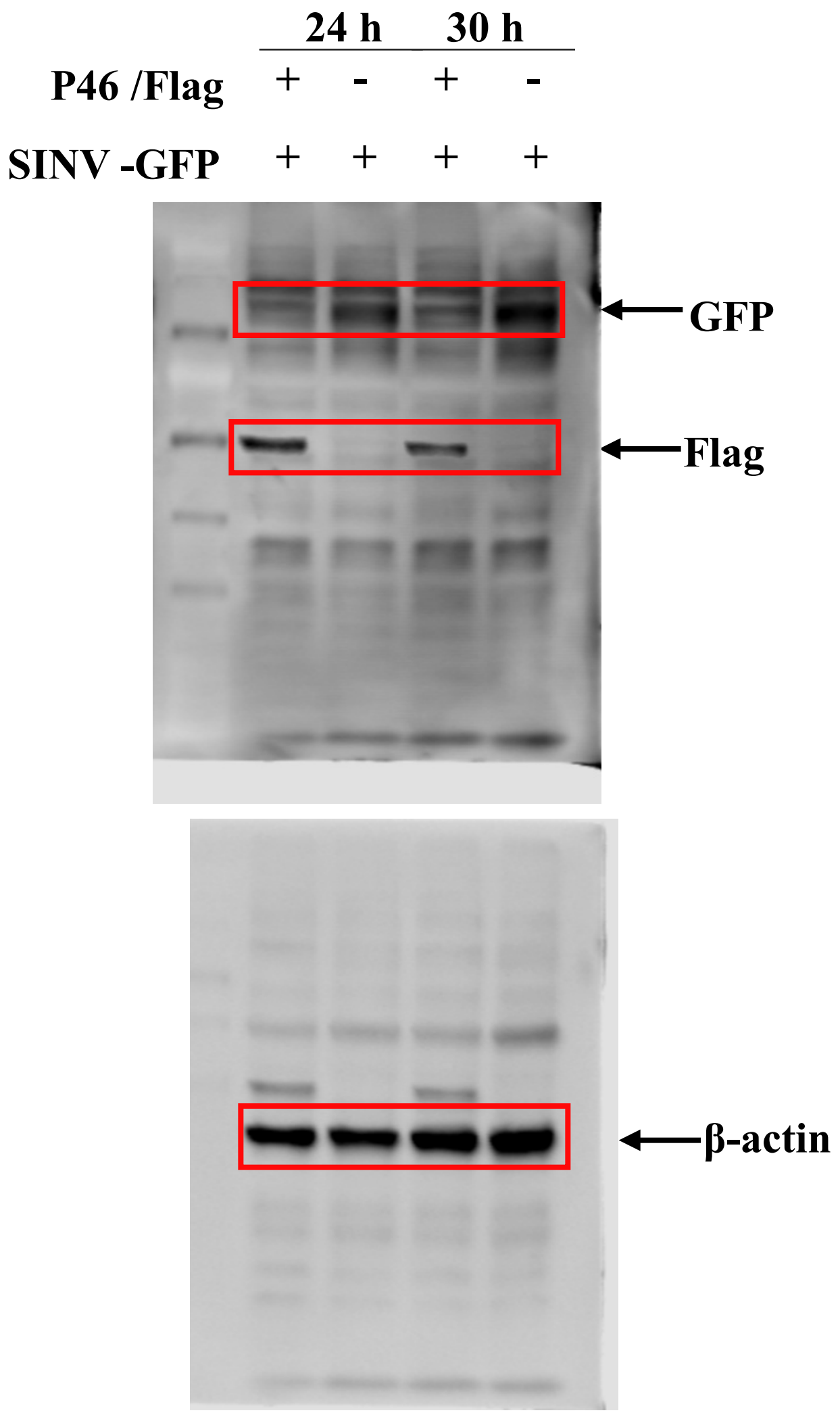

B

Vector control **P46**

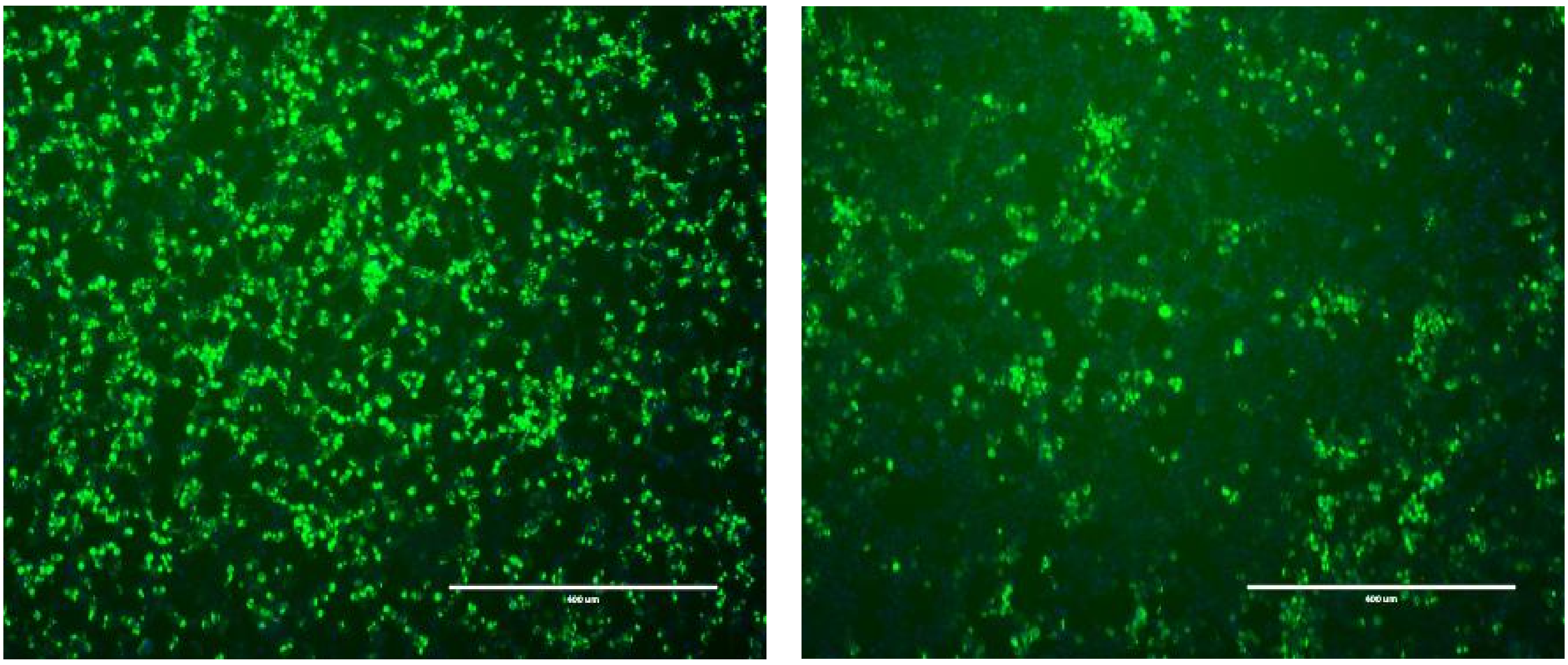

C

	repeat 1#	repeat 2#	repeat 3#	mean	SD
Vector control	75	85	60	73.333333	12.583057
P46	35	32	15	27.333333	10.785793

D

	repeat 1#	repeat 2#	repeat 3#	mean	SD
Vector control	6.9	6.3	6	6.4	0.4582576
P46	5.3	4.8	5.3	5.1333333	0.2886751

S9 Fig

A

	repeat 1#	repeat 2#	repeat 3#	mean	SD
WT	1	1	1	1	0
P46-/-	0.892103	1.135612	0.911231	0.9796487	0.1354064

B

	repeat 1#	repeat 2#	repeat 3#	mean	SD
WT	1	1	1	1	0
P46-/-	0.916573	0.994129	1.182936	1.0312127	0.1369989

D

	repeat 1#	repeat 2#	repeat 3#	mean	SD
WT	1	1	1	1	0
P46-/-	2.1356	1.6071	2.3241	2.0222667	0.3716929

S10 Fig

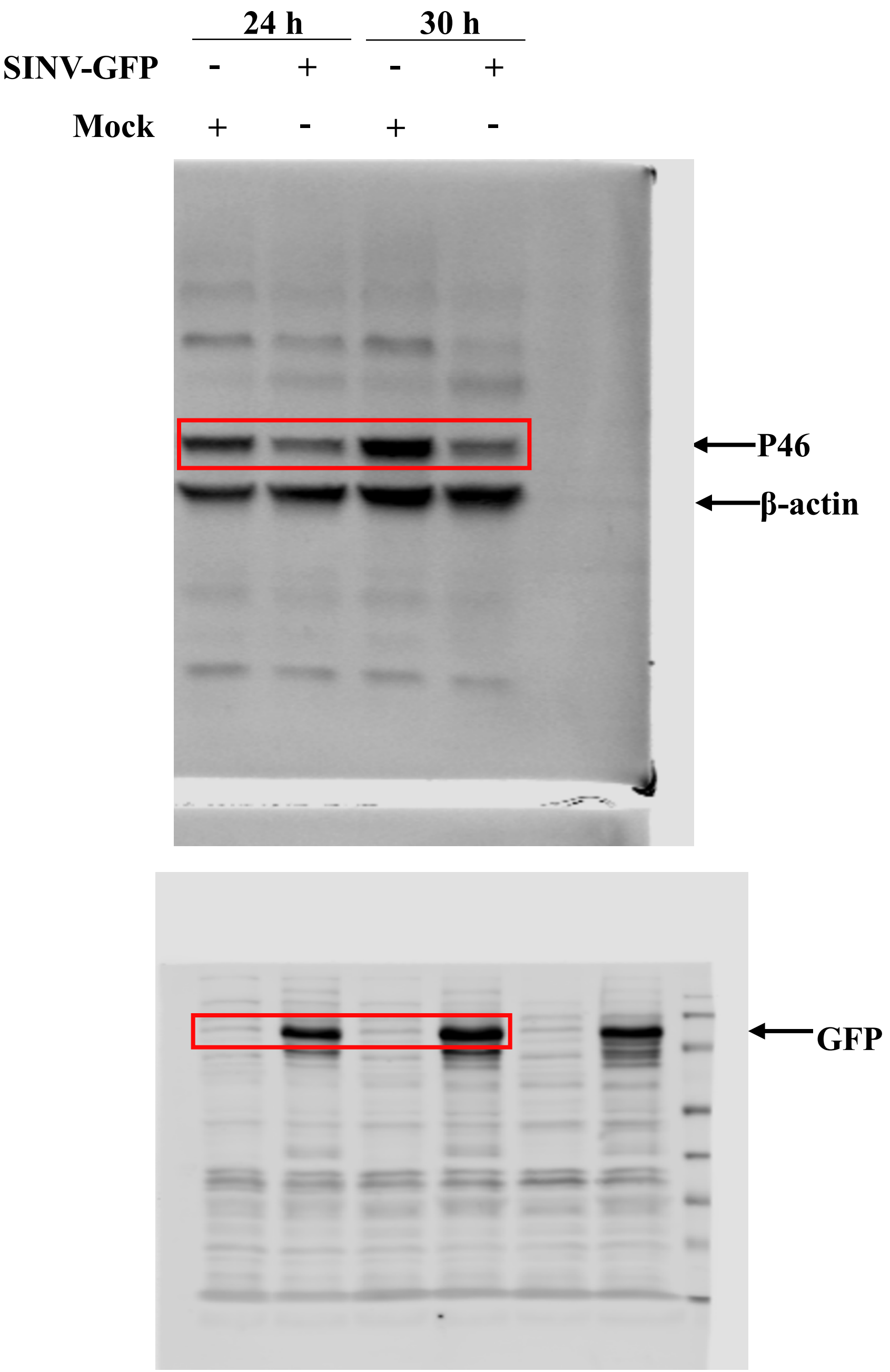

S11 Fig

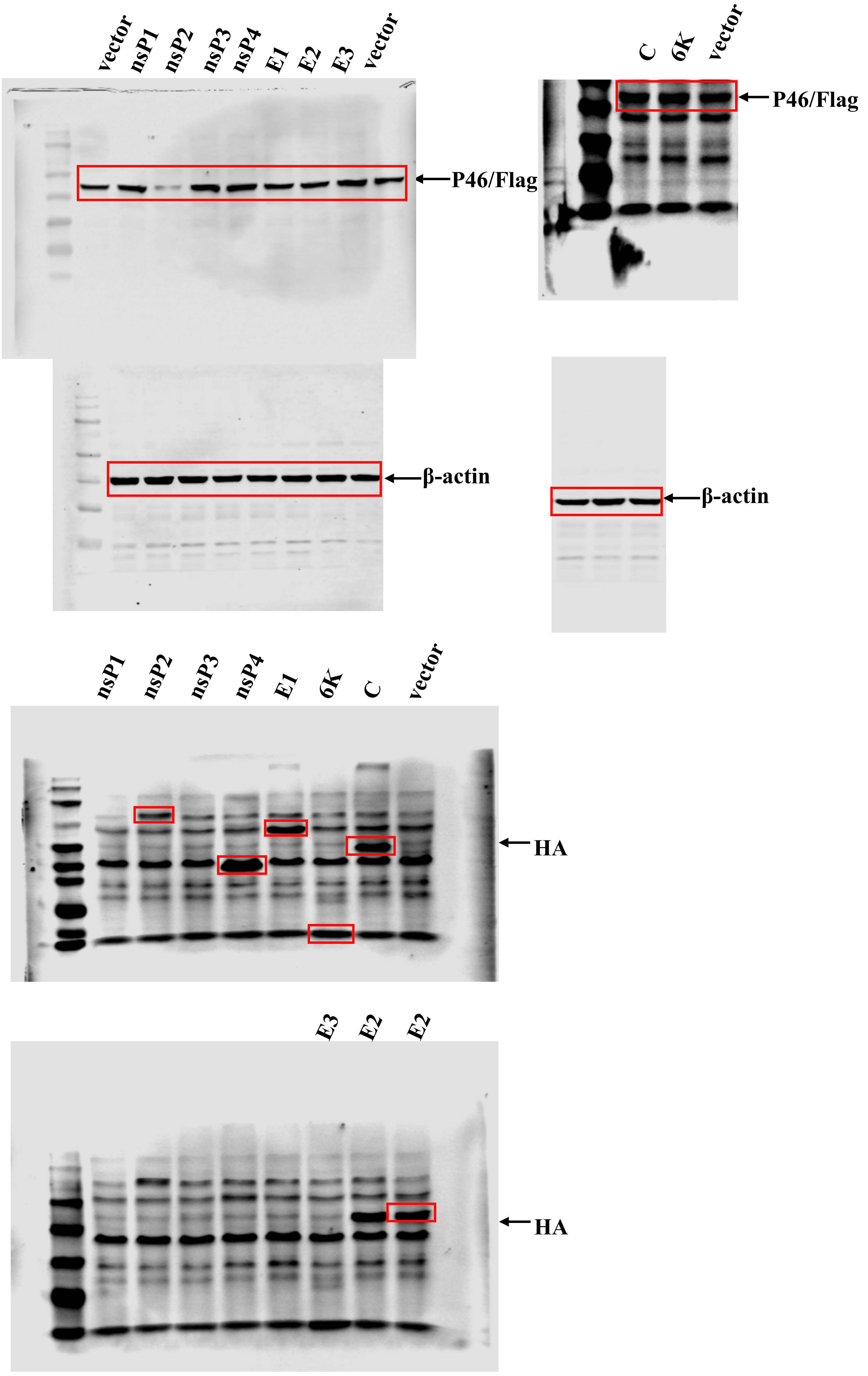
vector
nsP1
nsP2
nsP3
nsP4
E1
E2
E3
vector
P46/Flag
C
6K
vector
P46/Flag
β-actin
β-actin
nsP1
nsP2
nsP3
nsP4
E1
6K
C
vector
HA
E3
E2
E2
HA

S13 Fig

A

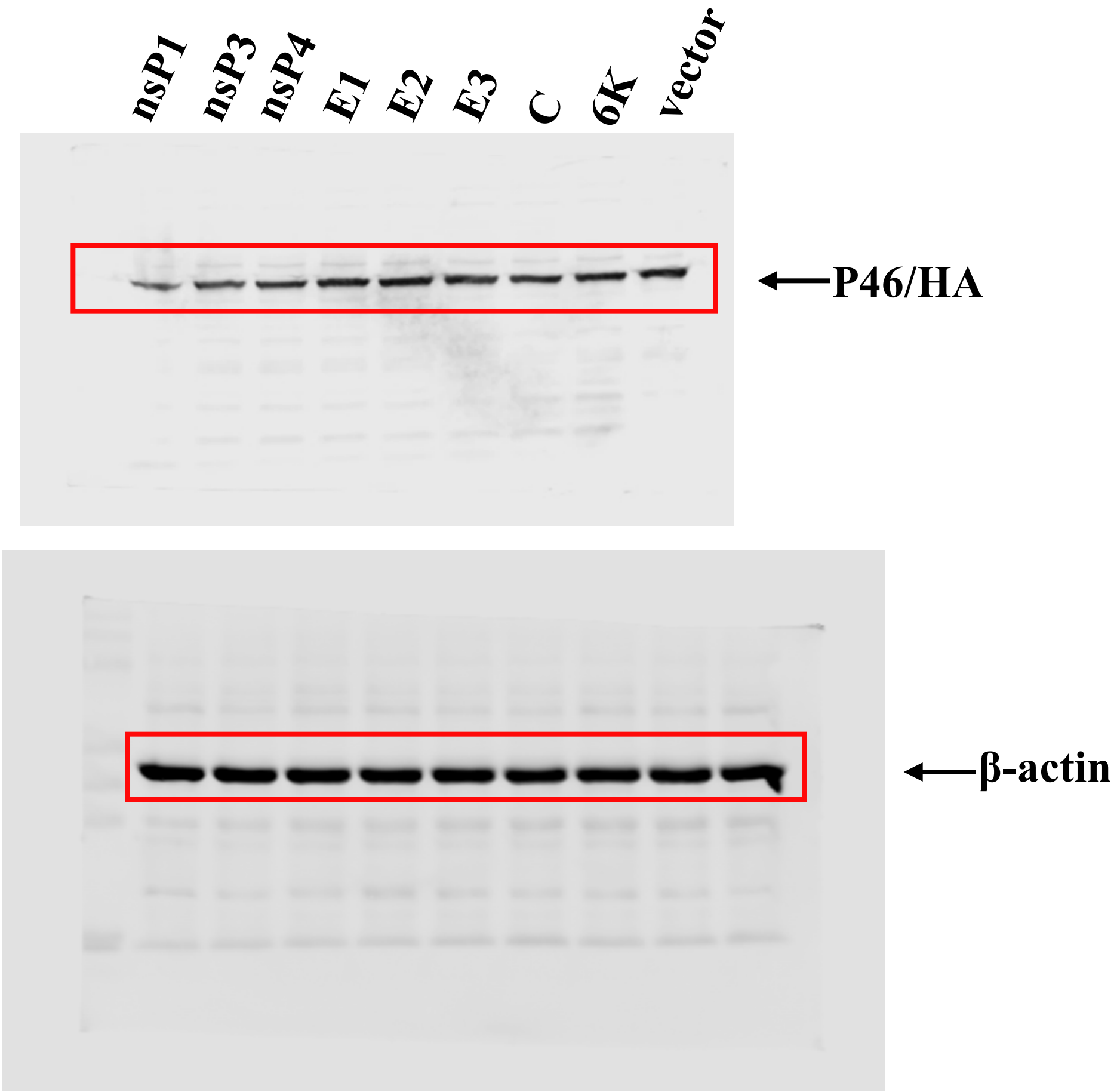
nsP1
nsP3
nsP4
E1
E2
E3
C
6K
vector
P46/HA
β-actin

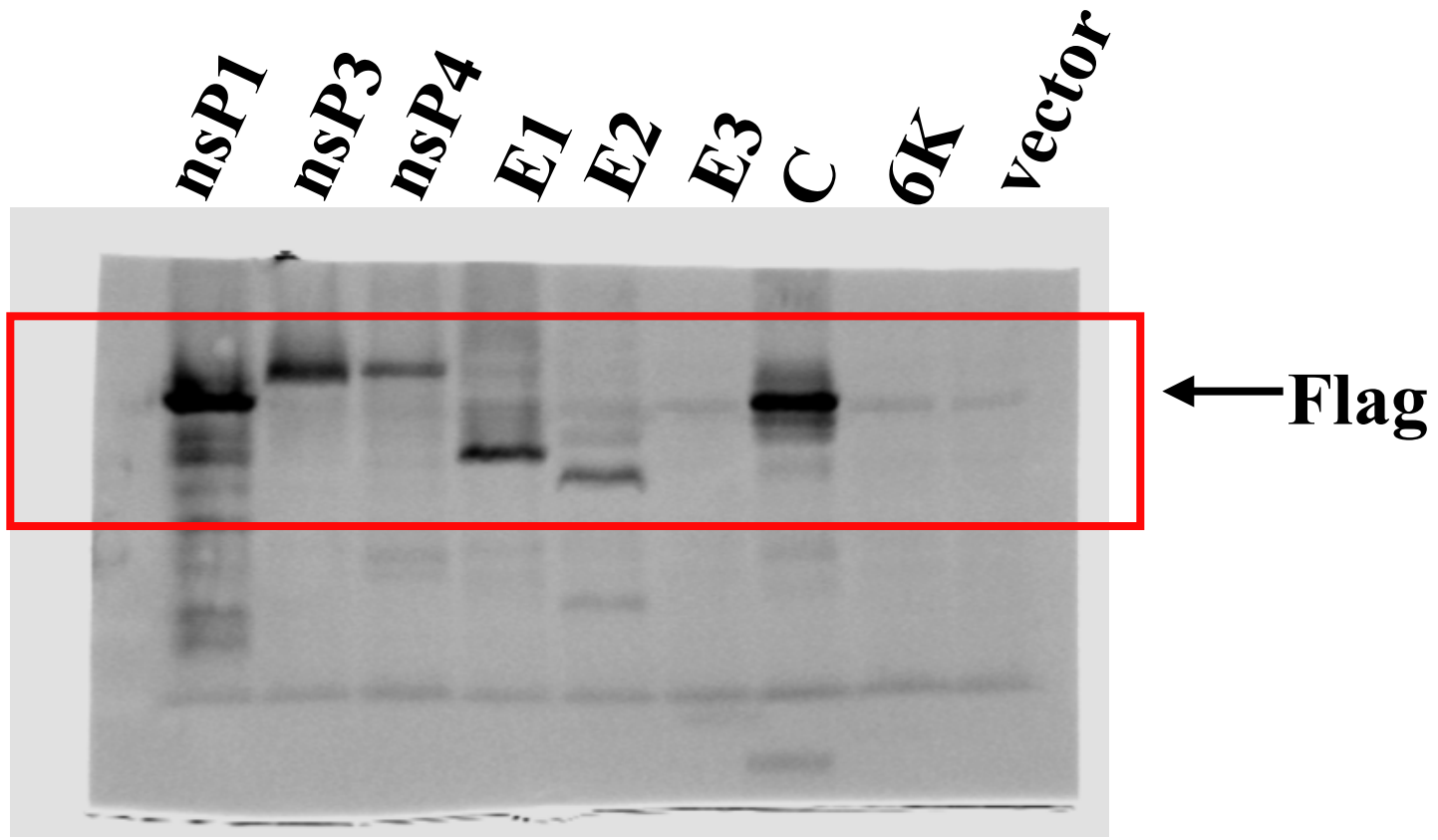
nsP1
nsP3
nsP4
E1
E2
E3
C
6K
vector
Flag

B

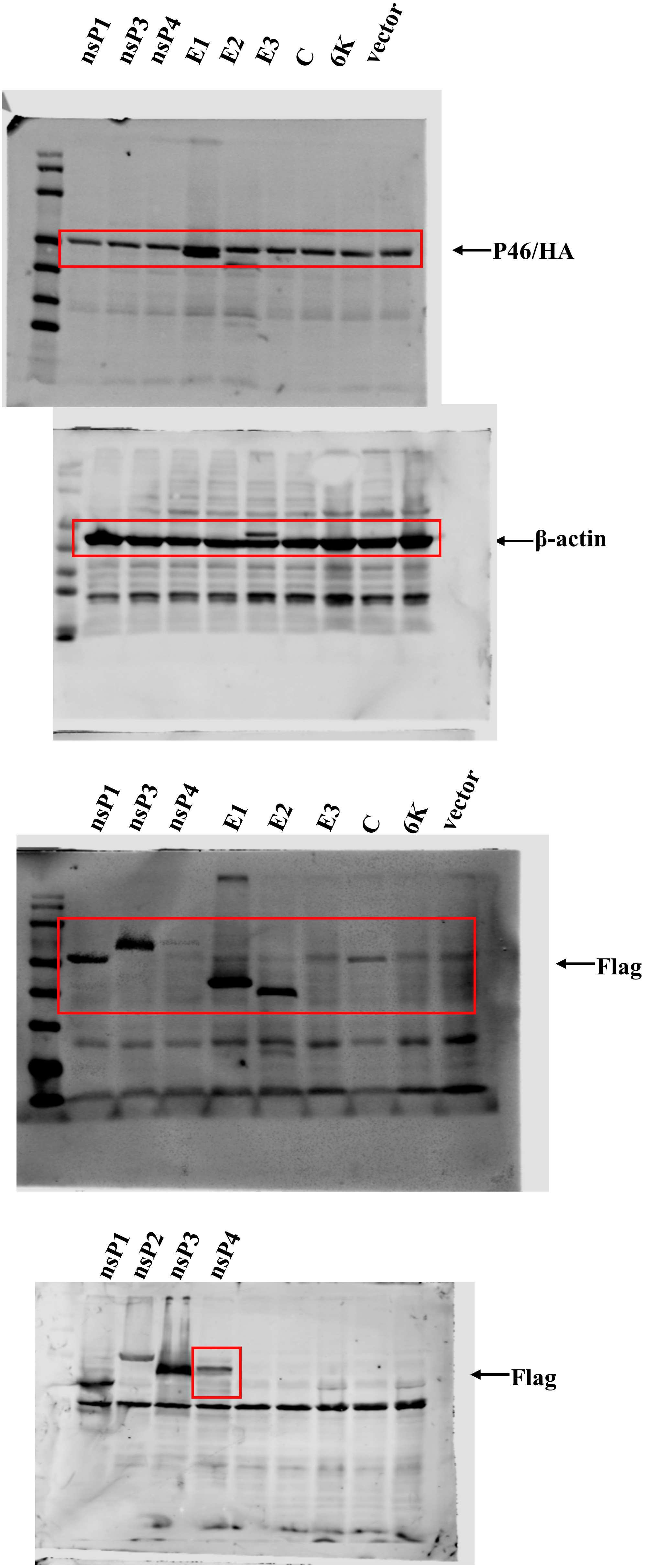

nsP1
nsP3
nsP4
E1
E2
E3
C
6K
vector
P46/HA
β-actin
nsP1
nsP3
nsP4
E1
E2
E3
C
6K
vector
Flag
nsP1
nsP2
nsP3
nsP4
Flag
