# Supplementary material for: Potential threats of porcine Getah virus outbreaks and its specific antagonism of host antiviral response through proteasome-dependent degradation of P46 by nsP2
Source: PLoS Pathog. 2026 Aug 26;22(8):e1014447. doi: 10.1371/journal.ppat.1014447 (PMC13515021; doi:10.1371/journal.ppat.1014447)
Supplement: Supplementary file 2 [file ppat.1014447.s014.docx]

Table S1. Primers used in this study

| Primers | Forward or reverse (5'→3') | Sequence |
| --- | --- | --- |
| **Construction of recombinant plasmids in pCAGGS：** | | |
| P46 (porcine) ^a^ | Forward | TTC**ATCGAT**ATGGCGGACATCTCCCTGGACGAGCTCAT |
|  | Reverse | GGA*AGATCT*TCACTTGTCGTCATCGTCTTTGTAGTCAAGTTTGATTTTGAATTCTGTGG |
| P46 (human)^a^ | Forward | TTC**ATCGAT**ATGGCGGACATCTCCCTGGACGAACTCAT |
|  | Reverse | GGA*AGATCT*TCACTTGTCGTCATCGTCTTTGTAGTCAAGCTTGATTTTGAATTCTGTGG |
| P46^b^ | Forward | GGA**AGATCT**ATGGCGGACATCTCCCTGGACGAAC |
|  | Reverse | CGG*GGTACC*TCAAAGTTTGATTTTGAATTCTGTG |
| nsP2-M1^c^ | Forward | CCG**GAATTC**ATGGGCGTGGTGGAGACACCCAGGA |
|  | Reverse | CCG*CTCGAG*CTAAGCGTAATCTGGAACATCGTATGGGTAATCCACCTCGGCAGGTCTCTCG |
| nsP2-M2^c^ | Forward | CCG**GAATTC**ATGCCTTTCCAGAATAAGGCCAAGG |
|  | Reverse | CCG*CTCGAG*CTAAGCGTAATCTGGAACATCGTATGGGTAATTGGAAGGCACGATATTGCAC |
| nsP2-M3^c^ | Forward | CCG**GAATTC**ATGAGGAGACTGCCTCACGCCCTGG |
|  | Reverse | CCG*CTCGAG*CTAAGCGTAATCTGGAACATCGTATGGGTAGCAGCCGGCGGTGTGCAGGCCA |
| nsP2-M4^c^ | Forward | CCG**GAATTC**ATGGGCGTGGTGGAGACACCCAGGA |
|  | Reverse | CCG*CTCGAG*CTAAGCGTAATCTGGAACATCGTATGGGTAGCCCTCGTAGGCGAACTCGTGA |
| nsP2-M5^c^ | Forward | CCG**GAATTC**ATGCTGAAGATCAGACCTGCCGCCC |
|  | Reverse | CCG*CTCGAG*CTAAGCGTAATCTGGAACATCGTATGGGTAACATCTTCTGGAGATGCTCTTG |
| nsP2-M6^c^ | Forward | CCG**GAATTC**ATGACACTGCCTGTGACAGCCATCG |
|  | Reverse | CCG*CTCGAG*CTAAGCGTAATCTGGAACATCGTATGGGTAATCCACCTCGGCAGGTCTCTCG |
| **Construction of recombinant plasmids in pLVX-IRES-ZsGreen1^d^：** | | |
| pLVX-P46 | Forward | AG**ACTAGT**ATGGCGGACATCTCCCTGGACGAGCTCAT |
|  | Reverse | GA*GCGGCCGC*TCACTTGTCGTCATCGTCTTTGTAGTCAAGTTTGATTTTGAATTCTGTGG |
| **Real-time PCR (qRT-PCR)^e^：** | | |
| qGETV-E2 | Forward | ACCACCAGTAGCGACAAGAC |
|  | Reverse | TTGCCTTCAGTTGTCAGCTG |
| qP46 | Forward | CAAAACCATCCAGGTTCCAC |
|  | Reverse | TCCGGGACATGTTGGTCAAG |
| qβ-actin | Forward | CTTCCTGGGCATGGAGTCC |
|  | Reverse | GGCGCGATGATCTTGATCTTC |
| **Primers for detecting samples^f^：** | | |
| PEDV | Forward | AGGGCGTTTGTATAGAGT |
|  | Reverse | CTTTGGCACAGTCATTTT |
| TGEV | Forward | GGAAGAGAACTGCAGGTAAA |
|  | Reverse | ATCTCAACCTGTGTGTCATC |
| PDCoV | Forward | GGCTACTGGCTGCGTTAC |
|  | Reverse | GCTTGATGCTGGGTCTTA |
| PoRV | Forward | AAAAGGGATTTCCGTTGG |
|  | Reverse | CAATAAAGTTGTGGGTCA |
| GETV | Forward | GTTTCTGCCCTGACGACC |
|  | Reverse | AACCTGCCACCGCTGTAC |
| P46 KO identification | Forward | TCCAGCAGAGATTTGATGCC |
|  | Reverse | CTGGATGGTTTTAGTGAGCTT |

^a^A Flag tag (underlined) was fused at the carboxyl terminus of P46 by PCR and was cloned into the Cla I (bold) and BglⅡ(italic) sites in the pCAGGS vector.

^b^A P46 gene was cloned into the BglⅡ(bold) and Kpn I (italic) and sites in the pAcGFP1-C1 vector.

^C^A HA tag (underlined) was fused at the carboxyl terminus of nsP2 mutant by PCR and was cloned into the EcoR I (bold) and Xho I (italic) sites in the pCAGGS vector.

^d^A Flag tag (underlined) was fused at the carboxyl terminus of P46 by PCR and was cloned into the SpeⅠ(bold) and NotⅠ(italic) sites in the pLVX-IRES-ZsGreen1

^e^Used for relative quantitative PCR.

^f^Used for detecting samples.
